# Mechanochemical halogenation of unsymmetrically substituted azobenzenes

**DOI:** 10.3762/bjoc.18.69

**Published:** 2022-06-15

**Authors:** Dajana Barišić, Mario Pajić, Ivan Halasz, Darko Babić, Manda Ćurić

**Affiliations:** 1 Division of Physical Chemistry, Ruđer Bošković Institute, Bijenička cesta 54, Zagreb, Croatia

**Keywords:** azo compounds, halogenation, mechanochemistry, *N*-halosuccinimide, palladium(II)

## Abstract

The direct and selective mechanochemical halogenation of C–H bonds in unsymmetrically substituted azobenzenes using *N*-halosuccinimides as the halogen source under neat grinding or liquid-assisted grinding conditions in a ball mill has been described. Depending on the azobenzene substrate used, halogenation of the C–H bonds occurs in the absence or only in the presence of Pd^II^ catalysts. Insight into the reaction dynamics and characterization of the products was achieved by in situ Raman and ex situ NMR spectroscopy and PXRD analysis. A strong influence of the different 4,4’-substituents of azobenzene on the halogenation time and mechanism was found.

## Introduction

Electrophilic aromatic substitution [1–3] and ligand-directed transition-metal-catalyzed reactions [4–8] are among the most widely used synthetic approaches for the preparation of halogenated arenes. They are important precursors in cross-coupling reactions [9–19] or components of pharmaceuticals and biologically active molecules [20–21]. The synthetic aspects of both approaches in solution are well established.

The need for green and sustainable chemistry [22] has led to the development and synthetic application of solid-state methods [23–46], particularly ball milling [26–46], which has proven to be an environmentally friendly and powerful alternative to conventional solvent-based protocols, offering unique advantages in terms of sustainability, reaction times, yields, reactant solubility, selectivity, and chemical reactivity. Although ball milling methods are widely used for the synthesis of various classes of compounds [26–46], their application in the synthesis of halogenated arenes is still scarce.

In 2015, Bolm and Hernandez reported the halogenation of 2-phenylpyridine in a ball mill using [Cp*RhCl_2_]_2_ in combination with AgSbF_6_ as catalyst and *N*-halosuccinimide (NXS, X = Br, I) as halogen source [47]. Two years later in 2017, Eslami's group applied a ball-milling method to synthesize aryl bromides and α-bromoketones with *N*-bromosuccinimide (NBS) and MCM-41-SO_3_H catalyst and no liquid additives [48]. In 2018, Wang and co-workers developed the ball-milling protocol for the *ortho*-halogenation of acetanilide with NXS (X = Cl, Br, I) using Pd(OAc)_2_ as precatalyst in the presence of *p*-toluenesulfonic acid (TsOH) as an additive under solvent-free conditions [49]. Recently, Mal and Bera reported the utilization of NXS (X = Br, Cl) as bifunctional reagents for the solvent-free synthesis of phenanthridinones via a cascaded oxidative C–N coupling followed by a halogenation reaction [50].

**Figure 1:**
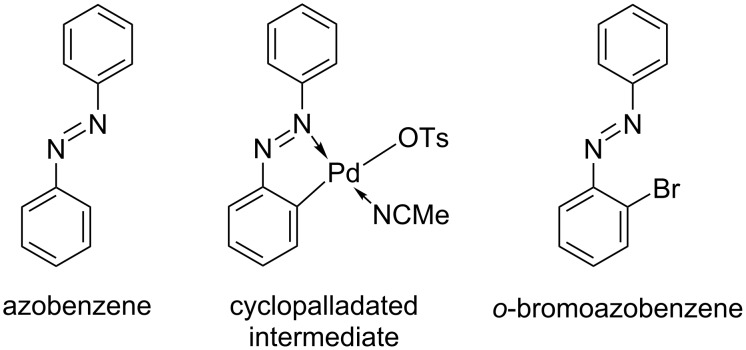
Molecular structures of the monomeric cyclopalladated intermediate and brominated product observed during the bromination of **L1** under LAG conditions [51].

Only recently, our group carried out a detailed synthetic and mechanistic study of the mechanochemical Pd^II^-catalyzed bromination of unsubstituted azobenzene (**L1**) by *N*-bromosuccinimide (NBS) under neat grinding (NG) and liquid-assisted grinding (LAG) conditions in a ball mill [51]. Insight into the dynamics of the formation of reaction intermediates and products was obtained by in situ Raman monitoring that provided information on the nature of the catalytically active Pd^II^ species, cyclopalladated intermediates, and products (Figure 1). The monitoring results confirmed the crucial role of TsOH and acetonitrile (MeCN) as additives in the catalytic bromination of the C–H bond in **L1**. The experimental results were supported by quantum-chemical calculations, which showed that four mechanistic pathways could be involved in this reaction [51]. Three of them involve oxidative addition followed by reductive elimination. Neutral NBS or the hydrogen bond complex NBS∙∙∙TsOH are bromine donors in two of them, while protonated NBS is engaged in the third. The fourth mechanism proceeds by electrophilic cleavage with neutral NBS or the hydrogen bond complex NBS∙∙∙TsOH as a bromine source.

Here we present the mechanochemical selective halogenation of unsymmetrically substituted azobenzenes by NXS (X = Cl, Br, or I). The liquid-assisted grinding of *para*-halogenated derivatives of azobenzene with NXS and Pd(OAc)_2_ as precatalyst in the presence of TsOH and MeCN as solid and liquid additives, respectively, led to the *ortho*-halogenated products relative to the azo group of the azobenzenes. In situ Raman monitoring of these reactions confirmed that the most favorable reaction pathway is via the monomeric cyclopalladated intermediate, as in the halogenation reaction of unsubstituted **L1** [51]. While the reactions of **L1** and its *para*-halogenated derivatives were unsuccessful without the Pd^II^ catalyst and TsOH, the halogenation of azobenzenes with the strong electron-donating substituents in the *para* position occurred in the absence of the added Pd^II^ catalyst and additives, in the *ortho* position to the substituent, which is typical for the products of electrophilic aromatic substitution.

In addition, an additive- and solvent-free protocol without the added Pd^II^ catalyst was developed for the selective imidation of azobenzenes containing a dimethylamino group as substituent in the *para* position to the azo group.

## Results and Discussion

Inspired by our findings on the mechanochemical halogenation of **L1** [51] and the report of Ma and Tian on the bromination of symmetric and unsymmetric azobenzenes in MeCN [52], we investigated how substituents of different donor strength affect the selectivity, reactivity, and reaction pathways of halogenation of azobenzene substrates under mechanochemical conditions.

### Halogenation of azobenzenes with strong electron-donating substituents

**Scheme 1:**
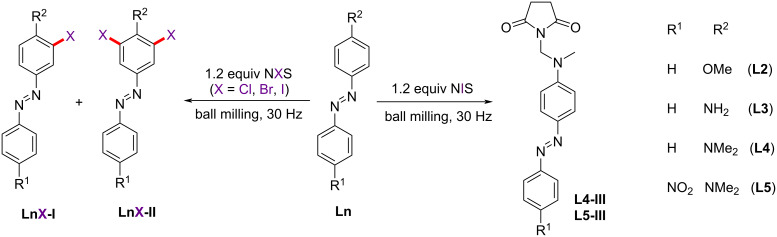
Halogenation of azobenzenes with strong electron-donating substituents.

In contrast to the reactions of **L1** [51], the halogenation of azobenzene substrates containing strong electron-donating substituents (4-methoxyazobenzene (**L2**), 4-aminoazobenzene (**L3**), 4-dimethylaminoazobenzene (**L4**), and 4-(dimethylamino)-4'-nitroazobenzene (**L5**)) with NXS (X = Cl and Br) occurred in the absence of the added Pd^II^ catalyst and additives. These reactions in most cases produced electrophilic substitution products that were halogenated in the *ortho* position(s) relative to the electron-donating substituent (Scheme 1 and Table 1), as confirmed by Raman (Figures S14–S22 in Supporting Information File 1) and NMR spectroscopy (Figures S40–S76 in Supporting Information File 1). All experiments were performed without additional oxidants and solid or liquid additives. The presence of the Pd^II^-catalyst in the reactions of **L2**–**5** with NXS resulted predominantly in **LnX**-**I** products or a mixture of products that were mono- or dihalogenated at the *ortho* and *meta*-position(s) relative to the electron-donating substituent, which may be attributed to competition between Pd^II^-catalyzed reactions and uncatalyzed electrophilic substitution.

**Table 1:** Halogenation of azobenzenes with strong electron-donating substituents.^a^

Entry	Reactant	NXS	Product	*t* [h]	Yield [%]^b^

1	**L2**	NCS	–	7	–
2	**L3**	NCS	**L3Cl-I**	1	46 (34)
3	**L4**	NCS	**L4Cl-I**	1	85 (73)
4	**L5**	NCS	**L5Cl-I** **L5Cl-II**	15	83 (72) 16 (8)^c^
5	**L2**	NBS	**L2Br-I**	15	96 (90)
6	**L3**	NBS	**L3Br-I**	1	72 (54)
7	**L4**	NBS	**L4Br-I**	1	79 (67)
8	**L5**	NBS	**L5Br-I**	7	53 (37)
9	**L2**	NIS	**–**	7	–
10	**L3**	NIS	**L3I-I**	1	30 (21)
11	**L4**	NIS	**L4-III**	1	39 (29)
12	**L5**	NIS	**L5-III**	5	38 (31)

^a^Reaction conditions: 14 mL PMMA jar, mixer mill, one nickel bound tungsten carbide milling ball (7 mm in diameter, 3.9 g), 30 Hz, **L2**–**5** (0.50 mmol), NXS (0.60 mmol), SiO_2_ (250 mg); ^b^determined by ^1^H NMR spectroscopy using 1,4-dinitrobenzene as the internal standard, with isolated yield given in parentheses; ^c^yield calculated with respect to **L5**.

Our results are consistent with those reported by Sanford's group for the halogenation of various substrates by NXS with and without Pd^II^ catalyst [53]. Most of these reactions were carried out in MeCN and AcOH solutions at 100–120 °C. In contrast, the bromination of 4-methoxyazobenzene by NBS in MeCN at ambient temperature, reported by Tian and Ma [52], resulted in an electrophilic monobrominated product as a single isomer in both the catalyzed and uncatalyzed reactions.

Neat grinding of **L3** and **L4** with NCS produced the monochlorinated products **L3Cl-I** and **L4Cl-I** as single isomers within one hour (Table 1, entries 2 and 3). In contrast, the reaction of **L5** with NCS resulted in a mixture of mono- and dichlorinated regioisomers (**L5Cl-I** and **L5Cl-II**) (Scheme 1 and Table 1, entry 4). The chlorination of **L3** substrate with a primary amine as substituent gave the monochlorinated product **L3Cl-I** in 46% yield, while the yields of **L4Cl-I** and **L5Cl-I** were 85% and 83%, respectively (Table 1, entries 2–4). Although both substrates **L4** and **L5** contain a tertiary amine as a substituent (NMe_2_), the chlorination of **L5** proceeded much more slowly (Table 1, entries 3 and 4).

**Figure 2:**
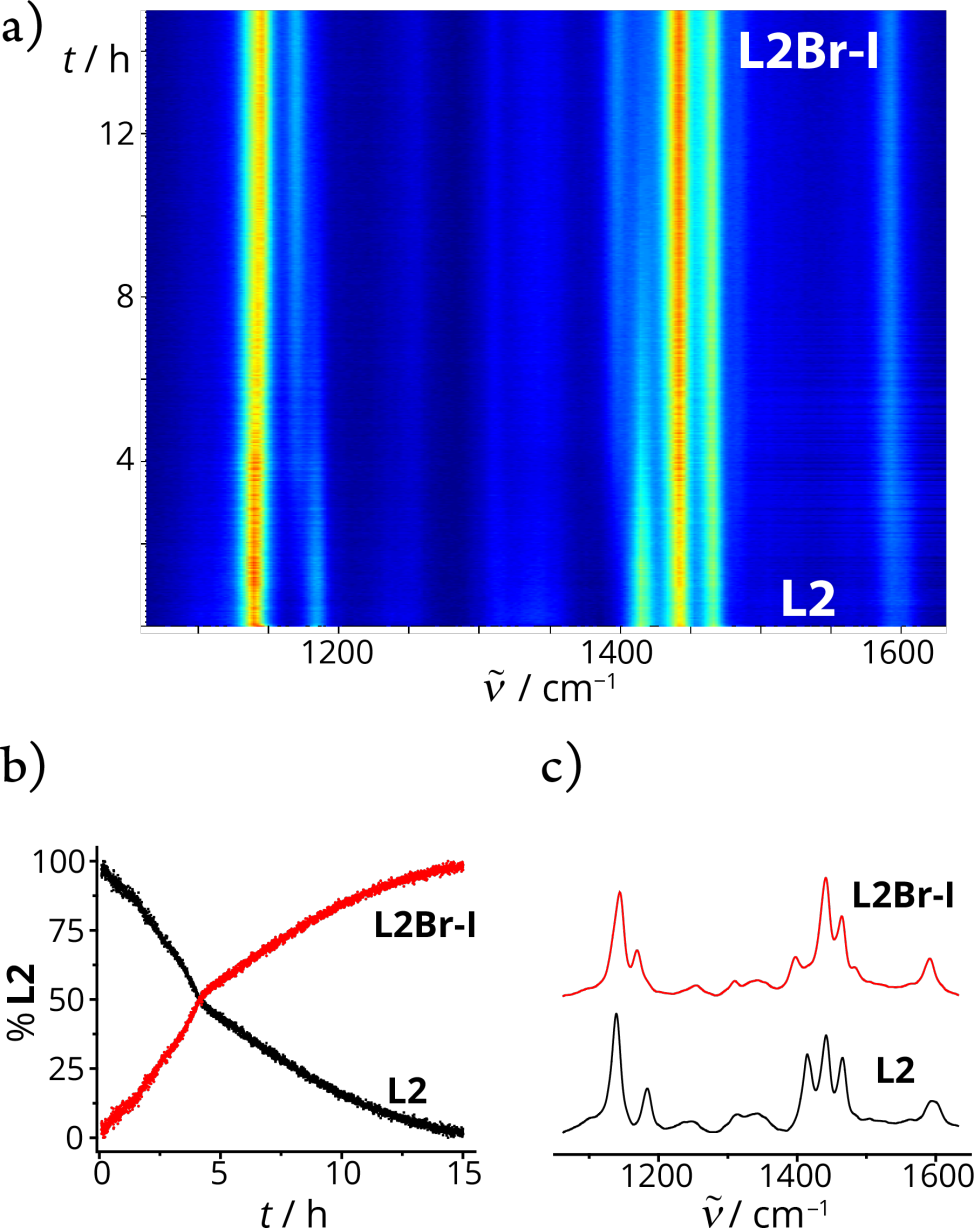
a) Two-dimensional (2D) plot of the time-resolved Raman monitoring of NG of **L2** (0.50 mmol) with NBS (0.60 mmol) and SiO_2_ (250 mg). b) Reaction profile derived from multivariate curve analysis - alternating least squares fitting (MCR-ALS analysis). c) Extracted Raman spectra of species observed during Raman monitoring.

Neither NCS nor NIS yielded halogenated products with substrate **L2** (Table 1, entries 1 and 9). However, the reaction of **L2** with NBS gave the monobrominated product **L2Br-I** in 96% yield after 15 hours of milling (Figure 2, Table 1, entry 5). The yield of this reaction was higher than the analogous reaction in MeCN solution (90% isolated yield under the mechanochemical conditions compared to 72% in MeCN solution) [52]. In situ Raman monitoring of the bromination of **L2** confirmed its conversion to the product **L2Br-I** (Figure 2). The formation of **L2Br-I** was accompanied by the intensity decrease of **L2** *ν*(N=N) and *ν*(C–N) bands in the range 1400–1450 and 1080–1200 cm^−1^, respectively. Compounds **L2** and **L2Br-I** were identified in the reaction mixture by the Raman spectra of isolated **L2** and **L2Br-I** (Figure 2c).

Neat grinding of **L3**, **L4**, and **L5** with NBS also produced the monobrominated products in yields ranging from 53 to 79% within one and seven hours, respectively (Table 1, entries 6–8).

The reaction of **L3** with NIS resulted in the monoiodinated product in a low yield of 30% after one hour of milling (Table 1, entry 10 and Figures S77–S81 in Supporting Information File 1). Interestingly, in the reactions of **L4** and **L5** with NIS, instead of the halogenation of the aromatic C–H bond, imidation of the aliphatic C–H bond was observed. Imides are among the most studied functional groups, and new methods for their preparation, especially by environmentally friendly protocols, are of great synthetic importance in organic chemistry [54].

**Figure 3:**
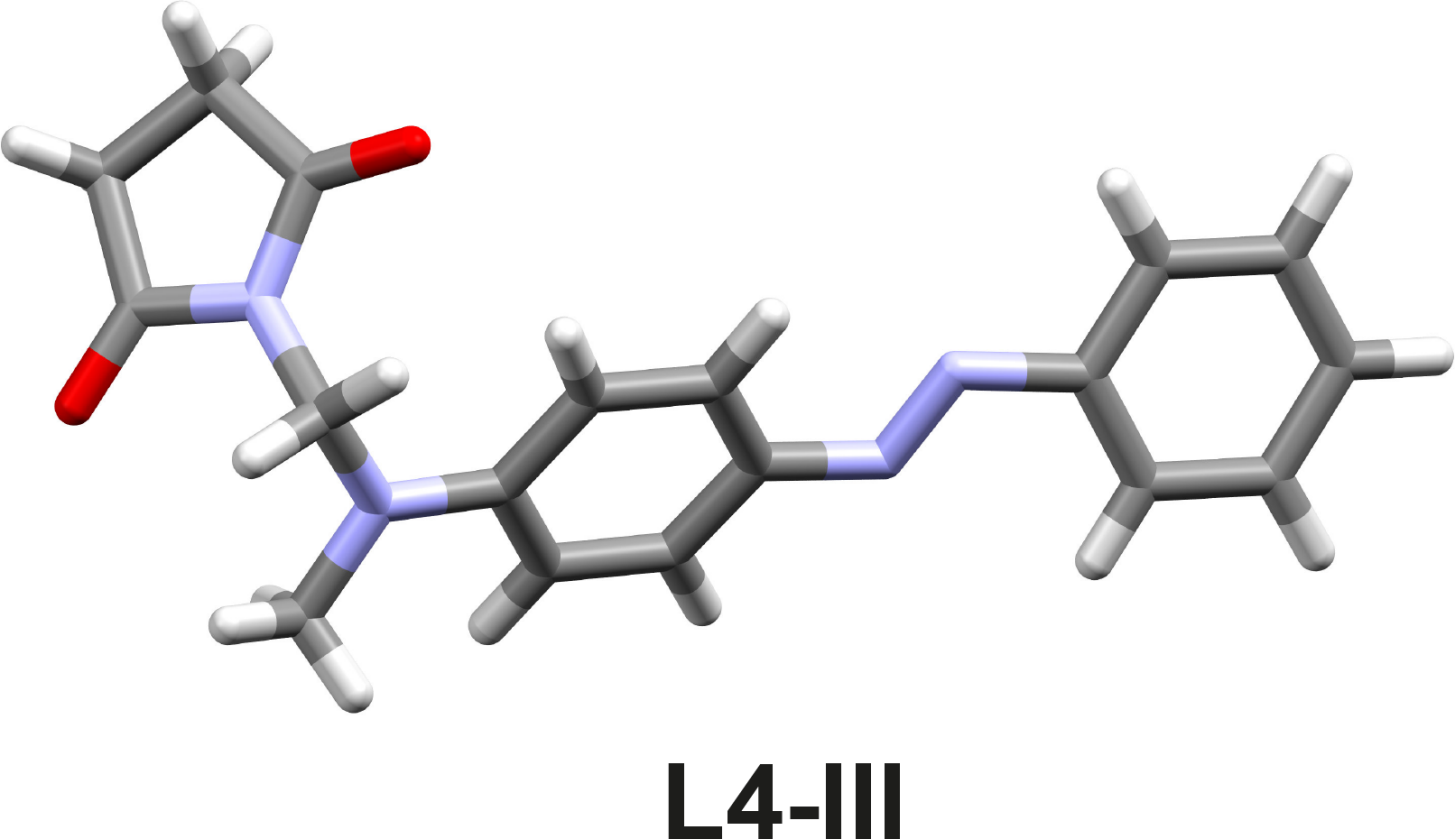
Experimental X-ray molecular structure of succinimide product **L4-III**.

The succinimide products **L4-III** and **L5-III** (Table 1, entries 11 and 12) were obtained within one and five hours in 39 and 38% yields, respectively, as confirmed by NMR spectroscopy (Figures S82–S91 in Supporting Information File 1). Additional support for the formation of succinimide products was provided by the molecular structure of **L4-III**, resolved by single-crystal X-ray analysis (Figure 3 and Figure S33 and Table S1 in Supporting Information File 1). The molecular structure of **L4-III** showed that imidation occurred at the methyl group of the NMe_2_ substituent. Analogous succinimide species were also observed in the reaction of *N,N*-dimethyl-*p*-toluidine with NIS in ethyl acetate [55] or *N,N*-dimethylamides and *N,N*-dimethylamines with NBS in carbon tetrachloride [56].

The reactivity trend of electron-rich azobenzenes **L2**–**5** toward NXS was also investigated. Results of competition experiments clearly demonstrated that in the case of NCS their reactivity decreases in the order: **L4** > **L3** > **L5** (Figures S1 and S2 in Supporting Information File 1), in the case of NBS in the order: **L3** > **L4** > **L5** >> **L2** (Figures S3–S6 in Supporting Information File 1), and in the case of NIS in the order: **L4** >> **L5** for the succinimide products (Figure S7 in Supporting Information File 1). The presence of an electron-accepting substituent (NO_2_) at the *para* position of the second phenyl ring in **L5** significantly slowed the halogenation reaction and impaired the reactivity of the azobenzene. This result suggests that NO_2_ has a long-range effect that spreads through the azobenzene skeleton. Compared to the bromination of substrates **L3**–**5** containing amino substituents, the analogous reaction of **L2** is slower because the methoxy substituent has weaker donor strength than amino substituents [57].

The protocols described above provide a solvent- and additive-free approach without added Pd^II^ catalysts for the halogenation of Csp^2^–H and imidation of Csp^3^–H bonds of azobenzenes with electron-donating substituents by electrophilic activation with NXS.

### Halogenation of azobenzenes with electron-accepting substituents

**Scheme 2:**
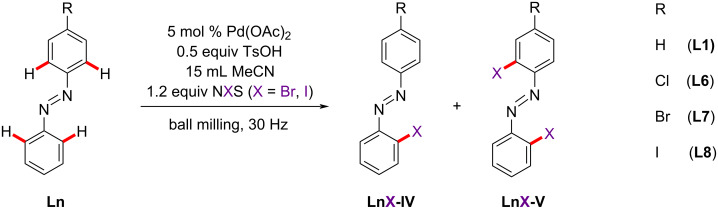
Pd^II^-catalyzed halogenation of azobenzene and its *para*-halogenated derivatives.

Using the optimal parameters for the mechanochemical bromination and iodination of **L1** [51], we investigated the halogenation of azobenzene substrates with electron-accepting substituents at the *para* position relative to the azo group: 4-chloroazobenzene (**L6**), 4-bromoazobenzene (**L7**), and 4-iodoazobenzene (**L8**) (Scheme 2 and Table 2).

**Table 2:** Pd^II^-catalyzed halogenation of **L1** [51] and its *para*-halogenated derivatives (**L6**–**8**).^a^

Entry	Reactant	NXS	Product	*t* [h]	Yield [%]^b^

1	**L1** [51]	NCS	–	17	–
2	**L6**	NCS	–	17	–
3	**L7**	NCS	–	17	–
4	**L8**	NCS	–	17	–
5	**L1** [51]	NBS	**L1Br-IV**	4	83 (74)
6	**L6**	NBS	**L6Br-IV**	6	73 (59)
7	**L7**	NBS	**L7Br-IV**	8	72 (60)
8	**L8**	NBS	**L8Br-IV**	8	62 (55)
9	**L1** [51]	NIS	**L1I-IV** **L1I-V**	4	38 (35) 43 (28)^c^
10	**L6**	NIS	**L6I-IV** **L6I-V**	6	66 (63) 17 (11)^c^
11	**L7**	NIS	**L7I-IV** **L7I-V**	5	69 (59) 19 (8)^c^
12	**L8**	NIS	**L8I-IV** **L8I-V**	7	52 (45) 19 (17)^c^

^a^Reaction conditions: 14 mL PMMA jar, mixer mill, one nickel bound tungsten carbide milling ball (7 mm in diameter, 3.9 g), 30 Hz, **L1** and **L6**–**8** (0.50 mmol), Pd(OAc)_2_ (5 mol %), NXS (0.60 mmol), TsOH (0.25 mmol), MeCN (15 µL), SiO_2_ (250 mg); ^b^determined by ^1^H NMR spectroscopy using 1,4-dinitrobenzene as the internal standard, with isolated yield given in parentheses; ^c^yield calculated with respect to **L1** or **L6**–**8**.

The synthetic protocols included milling the mixture of **Ln**/NXS/TsOH 1:1.2:0.5 equiv, 5 mol % Pd(OAc)_2_ precatalyst, and 15 µL MeCN as liquid additive. Under these conditions, the reactions of **L6**–**8** with NBS resulted in moderate to good yields of monobrominated products **LnBr-IV** (Table 2, entries 6–8).

The bromination of **L6**–**8** occurred regioselectively at the *ortho* position of the unsubstituted phenyl ring, as shown by NMR spectroscopy (Figures S92–S103 in Supporting Information File 1). The reaction times required for bromination increased in the order **L1** < **L6** < **L7** = **L8** (Table 2, entries 5–8). Dibrominated products **LnBr-V** were not detected in any of these reactions. Since the complex Pd(OTs)_2_(MeCN)_2_ was identified as the active catalyst, formed in situ, in the bromination reaction of **L1** [51], the analogous reactions of **L6**–**8** were carried out using Pd(OTs)_2_(MeCN)_2_ as the catalyst instead of Pd(OAc)_2_. As expected, the yields of **LnBr-IV** products were comparable to those obtained in the reactions with the Pd(OAc)_2_ precatalyst.

The Pd^II^-catalyzed iodination of **L6**–**8** was conducted with *N*-iodosuccinimide (NIS) as the iodine source. The reaction time for the iodination of **L6** was the same as for the analogous bromination reaction (Table 2, entry 10). Iodination of **L7** and **L8** was completed within five and seven hours, respectively (Table 2, entries 11 and 12). Unlike bromination, iodination of **L6–8** with NIS resulted in a mixture of the mono- and diiodinated products at the *ortho* positions of one or both phenyl rings (**LnI-IV** and **LnI-V**, Scheme 2 and Table 2, entries 10–12), as confirmed by NMR spectroscopy (Figures S104–S130 in Supporting Information File 1). Compared to **L1** [51], iodination of its *para*-halogenated derivatives resulted in lower yields of diiodinated products (Table 2, entries 9–12) since the activation/halogenation of the C–H bond occurs preferentially at the unsubstituted azobenzene phenyl ring [57].

The reactions of **L6**–**8** with *N*-chlorosuccinimide (NCS) gave no chlorinated product, which was also observed in the reaction of **L1** with NCS (Table 2, entries 1–4) [51].

**Figure 4:**
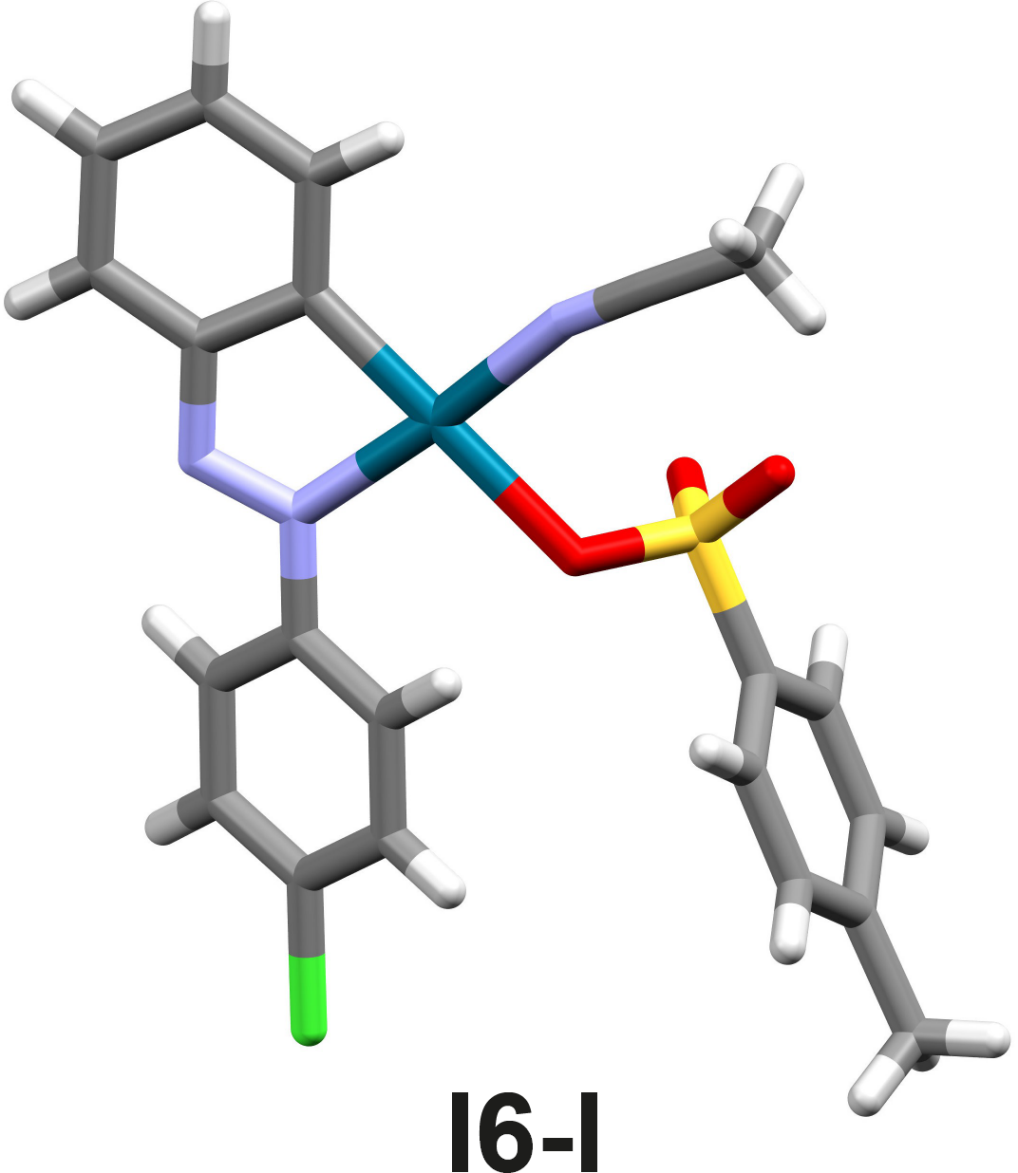
Experimental X-ray molecular structure of the intermediate **I6-I**.

Since the monomeric monopalladated tosylate complex of azobenzene **I1**-**I** was identified as an intermediate in the solid-state bromination of **L1** (Figure 1) [51], the analogous complexes of **L6** and **L7** were prepared to investigate whether the halogenation of the *para*-halogenated azobenzene derivatives follows the reaction pathway of the bromination of **L1**. The molecular structures of the isolated monopalladated tosylate complexes **I6**-**I** and **I7-I** solved from laboratory powder X-ray diffraction (PXRD) data (Figure 4 and Figures S31 and S32 in Supporting Information File 1), are similar to that of complex **I1-I** [51] in which the palladium center is bound to the MeCN and tosylate (OTs) via nitrogen and oxygen, respectively, and to the azobenzene via the azo nitrogen and a carbon atom of the unsubstituted phenyl ring. The tosylate ion is at the *trans* position to the carbon atom.

**Figure 5:**
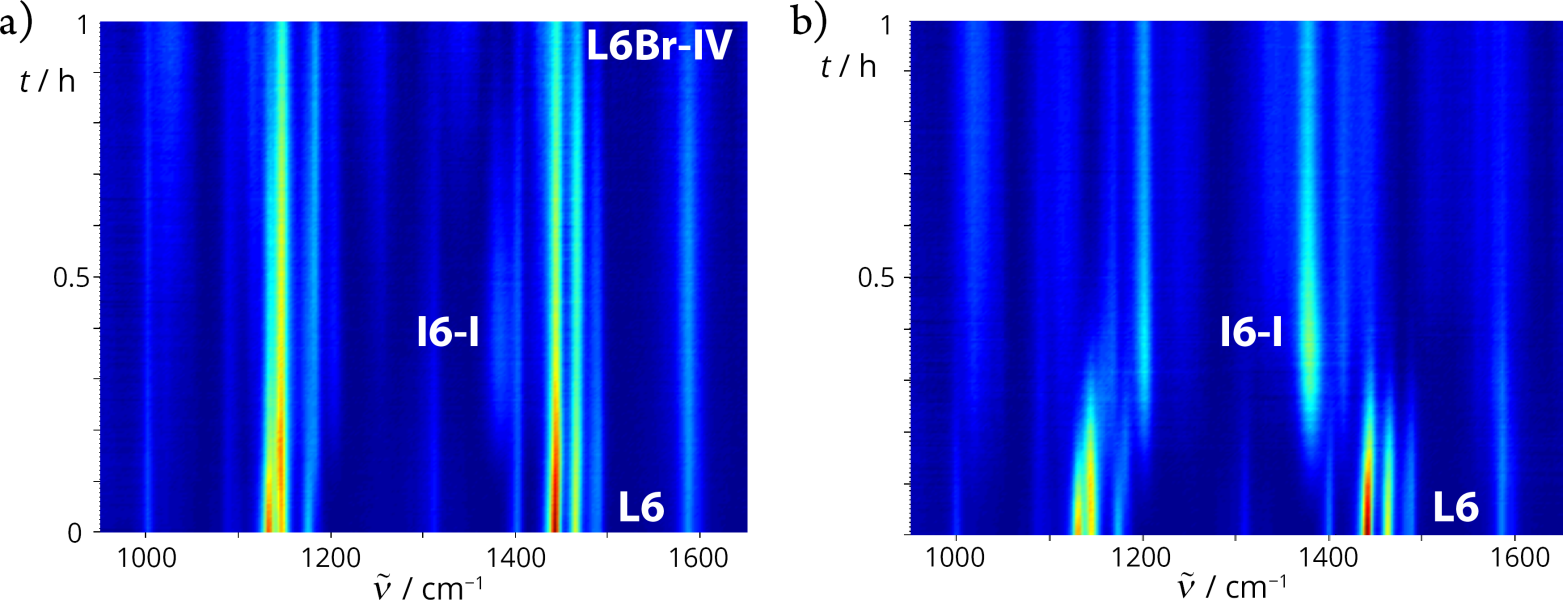
a) In situ observation of **I6**-**I** during the time-resolved Raman monitoring of LAG of **L6** (0.50 mmol) with NBS (0.6 mmol), TsOH (0.25 mmol), Pd(OAc)_2_ (30 mol %), MeCN (15 μL), and SiO_2_ (250 mg). b) Two-dimensional (2D) plot of the time-resolved Raman monitoring of LAG of **L6** (0.40 mmol) with Pd(OAc)_2_ (0.42 mmol), TsOH (0.42 mmol), MeCN (0.48 mmol, 25 µL), and SiO_2_ (250 mg). Only Raman spectra before fluorescence are shown in both parts.

In situ Raman monitoring of the bromination of **L6**–**8** in the presence of 30 mol % Pd^II^ catalyst revealed a new band around 1380 cm^−1^ (Figure 5a and Figures S23–S27 in Supporting Information File 1). It was assigned to the characteristic *ν*(N=N) bands of the **In-I** intermediates, confirming that the bromination of **L6**–**8** proceeds via monopalladated **In-I** intermediates as in the reactions of **L1** (Figure 5a and Figures S23–S27 in Supporting Information File 1) [51].

To gain insight into the dynamics of the formation of cyclopalladated intermediates **In-I**, LAG reactions of Pd(OAc)_2_ with **L6–8** and TsOH were performed using 25 µL of MeCN as a liquid additive in a molar ratio of 1:1:1 (**Ln**/Pd(OAc)_2_/TsOH). In situ Raman monitoring of C–H bond activation was possible for **L6** and **L7**, while in the case of **L8**, fluorescence prevented a more detailed insight into this reaction. The monitoring results confirmed the complete conversion of azobenzenes **L6** and **L7** to their monopalladated products after about one hour of milling, as well as a reaction course similar to that of the analogous reaction of **L1** [51] (Figure 5b and Figure S28 in Supporting Information File 1).

The isolated cyclopalladated intermediates **I6**-**I** and **I7**-**I** were tested as catalysts for the bromination of **L6** and **L7**. The yields of halogenated products in these reactions are 64% for **L6Br**-**I** and 70% for **L7Br**-**I**, which are close to those obtained in the analogous reactions with Pd(OAc)_2_ as precatalyst, as confirmed by NMR spectroscopy.

The results of in situ and ex situ spectroscopic monitoring along with the structural characterization of the intermediates have shown that the mechanism of the halogenation of azobenzenes with electron-accepting substituents is consistent with the proposed mechanistic schemes for the bromination of the unsubstituted azobenzene **L1** [51]. Thus, the halogenation of **L6**–**8** begins with the formation of the catalytically active Pd^II^ species, Pd(OTs)_2_(MeCN)_2_, from Pd(OAc)_2_, TsOH, and MeCN. It is followed by the formation of monomeric cyclopalladated intermediates **In-I**, at which halogenation occurs. Based on the similarities between the halogenation of **L6**–**8** and the bromination of **L1** [51], we assumed that the four mechanistic pathways considered for the bromination of **I1-I** are also possible for the halogenation of **I6-I**, **I7-I**, and **I8-I** [51].

In addition, we also investigated the reactivity trend of azobenzene **L1** and its *para*-halogenated derivatives **L6**–**8** toward NBS or NIS. The competition experiments showed that the azobenzenes with electron-withdrawing substituents are much less reactive to halogenation than **L1**. The reactivity of azobenzenes in the case of NBS decreases in this order: **L1** >> **L6** > **L7** ≈ **L8** (Figures S8–S10 in Supporting Information File 1), and in the case of NIS in the order: **L1** >> **L6** ≈ **L8** > **L7** (Figures S11–S13 in Supporting Information File 1), indicating that the Pd^II^-catalyzed halogenation of azobenzenes is strongly influenced by the nature of the azobenzene substituents.

## Conclusion

We have applied a mechanochemical protocol for the halogenation of 4,4’-functionalized azobenzenes in a ball mill under NG or LAG conditions, using NXS (X = Cl, Br, and I) as the halogen source.

Halogenation of azobenzenes with strong electron-donating groups was carried out without an added Pd^II^ catalyst. These transformations, which take place via electrophilic aromatic substitution, resulted in products halogenated in the *ortho* position to the electron-donating groups. The reactions of azobenzenes containing a dimethylamino group as substituent with NIS led to imidation products. A different reactivity of the dimethylamino group compared to the other substituents was also observed in these reactions.

On the other hand, the halogenation of *para*-halogenated azobenzenes required the presence of the Pd^II^ catalyst and TsOH as additive. In situ spectroscopic monitoring of these reactions revealed that the Pd^II^-catalyzed halogenation proceeds via monomeric cyclopalladated intermediates formed by activation of the C–H bond in azobenzenes with the in situ generated Pd(OTs)_2_(MeCN)_2_ catalyst. The described results indicate a strong dependence of the halogenation outcome of C–H bonds in 4,4’-functionalized azobenzenes on the nature of their substituents.

## Supporting Information

File 1 Detailed experimental procedures, complete characterization data for new compounds, X-ray structures of compounds, and the results of in situ Raman monitoring.

File 2 X-ray crystallographic data.
