# Supplementary material for: Mechanochemical halogenation of unsymmetrically substituted azobenzenes
Source: Beilstein J Org Chem. 2022 Jun 15;18:680–7. doi: 10.3762/bjoc.18.69 (PMC9235908; doi:10.3762/bjoc.18.69)
Supplement: File 1 — Detailed experimental procedures, complete characterization data for new compounds, X-ray structures of compounds, and the results of in situ Raman monitoring. [file Beilstein_J_Org_Chem-18-680-s001.pdf]

BEILSTEIN JOURNAL OF ORGANIC CHEMISTRY

Supporting Information

for

Mechanochemical halogenation of unsymmetrically substituted azobenzenes

Dajana Barišić, Mario Pajić, Ivan Halasz, Darko Babić and Manda Ćurić

Beilstein J. Org. Chem. **2022,** *18,* 680–687. doi:10.3762/bjoc.18.69

Detailed experimental procedures, complete characterization data for new compounds, X-ray structures of compounds, and the results of in situ Raman monitoring

License and Terms: This is a supporting information file under the terms of the Creative Commons Attribution License (https://creativecommons.org/licenses/by/4.0). Please note that the reuse, redistribution and reproduction in particular requires that the author(s) and source are credited and that individual graphics may be subject to special legal provisions.
The license is subject to the *Beilstein Journal of Organic Chemistry* terms and conditions: (https://www.beilstein-journals.org/bjoc/terms)

Table of contents

1. General S1

2. Synthesis S2

  2.1. Synthesis of **L6**–**8** S2

  2.2. Regioselective mechanochemical halogenation reactions with **L2**–**5** S3

  2.3. Regioselective mechanochemical halogenation reactions with **L1** and **L6**–**8** S7

3. Mechanistic studies S10

  3.1. Synthesis of **I6**-**I** S10

  3.2. Synthesis of **I7**-**I** S12

  3.3. *In situ* observation of **I6-I – I8-I** during the time-resolved Raman monitoring of mechanochemical bromination reactions S14

  3.4. Mechanochemical formation of **I6-I** and **I7-I** S14

  3.5. Competition experiments S15

4. Raman monitoring S28

  4.1. Halogenation of **L3**–**5** S28

  4.2. *In situ* observation of **I6**-**I–I8-I** during mechanochemical bromination reactions S33

  4.3. C–H bond activation S36

  4.4. Raman spectra of **I6**-**I** and **I7**-**I** S37

5. Powder X-ray diffraction S38

6. Single-crystal X-ray diffraction S40

7. NMR data S42

8. References S93

1. General

Solvents and chemicals used in this study were of reagent grade and were not additionally purified. Ligands **L6**, **L7,** and **L8** were prepared using the mechanochemical procedure. Complex $Pd(OTs)_2(MeCN)_2$ was obtained according to the literature procedure [1]. Reactions were monitored by thin-layer chromatography on the silica gel 60 F254 aluminum sheets.

Mechanochemical experiments were performed at the ambient temperature of 23 ± 2 °C using an IST500 mixer mill with a built-in fan operating at 30 Hz (www.insolidotech.com/ist500.html). Reactions were conducted in 14 mL polymethyl methacrylate (PMMA) transparent jars that allowed for in situ Raman monitoring or in 14 mL polytetrafluoroethylene (PTFE) jars. One nickel bound tungsten carbide (WC, 7 mm in diameter, 3.9 g) or two zirconium oxide (ZrO_2, 8 mm in diameter, 1.6 g) milling balls were used, and silica gel as a milling auxiliary.

NMR spectra were recorded on a Bruker Avance III HD 400 MHz/54 mm Ascend spectrometer. The temperature was kept constant at 25 °C and chemical shifts are reported in ppm and referenced to residual solvent signals.

HRMS spectra of novel compounds were obtained with a Bruker Microflex MALDI/TOF instrument.

Powder X-ray diffraction experiments that were used for crystal structure determination of compounds **I6-I** and **I7-I** were performed on a Panalytical Aeris desktop laboratory powder X-ray diffractometer in Bragg-Bretano geometry for 10 hours. Copper X-ray tube was operated at 40 kV and 7.5 mA, while the samples were prepared as thin films on zero-background silicon holders. Supplementary crystallographic data for the structures are available through the Cambridge Structural Database with deposition numbers 2159789 (**I6-I**) and 2159790 (**I7-I**).

Single crystals of **L4-III** were obtained by recrystallization of the purified product from hot ethyl acetate with a small amout of *n*-hexane (19:1). X-ray diffraction data of **L4-III** were collected by ω-scans on an XtaLAB Synergy diffractometer, Dualflex, HyPix using Cu$K\alpha$ (λ = 1.54184 Å) radiation at ambient temperature (293.15 K). The CrysAlis software package [2,3] was used for data reduction, while programs incorporated in the OLEX2 system [4] were used for solution, structural refinement, and analysis of the structure. The structure was solved and refined with the SHELX programme suite [5,6]. Structural refinement was performed on $F2$ using all data. All hydrogen atoms were placed at calculated positions and treated as riding on their parent atoms. Crystal data and other crystallographic experimental details are summarized in Table S1.

Drawings of the structures were prepared using MERCURY programs [7]. Supplementary crystallographic data set for the structure is available through the Cambridge Structural Database with deposition number 2159788.

Raman experiments were performed by the portable Raman system with PD-LD (now Necsel) BlueBox laser source with the 785 nm excitation wavelength, equipped with B&W-Tek fiber optic Raman BAC102 probe and coupled to OceanOptics Maya2000Pro spectrometer. Details of Raman experiments and processing of data have been described previously [8].

2. Synthesis

2.1. Synthesis of L6–8

General procedure for mechanochemical synthesis of **L6–8**

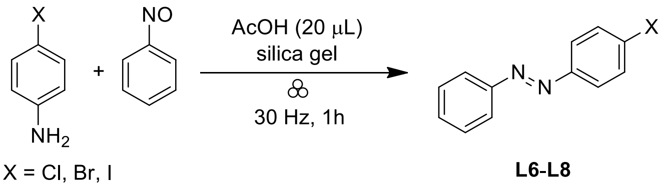

4-Halogenated aniline (0.8 mmol), nitrosobenzene (0.8 mmol), AcOH (20 μL), and silica gel (120 mg) were transferred to the 14 mL polytetrafluoroethylene (PTFE) jar loaded with two zirconium oxide (ZrO_2, 8 mm in diameter, 1.6 g) milling balls. The milling jar was placed in the mixer mill operating at 30 Hz, and the reaction mixture was milled at ambient temperature (23 ± 2 °C) for 1 h. The reaction mixture was scratched off from the jar, suspended in DCM (10 mL), and filtered through a thin layer of Celite. The mother liquid was collected, and the solvent was evaporated. The crude product was purified by column chromatography on silica gel (hexane/ethyl acetate 19:1) to obtain the desired product.

1-(4-Chlorophenyl)-2-phenyldiazene (**L6**):

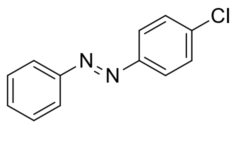

Following the general procedure gave the title compound as an orange solid (117.9 mg, 68 % isolated yield). Characterization data are consistent with previously published.[9] ^{1}H NMR ($CDCl_3$, 400 MHz): δ 7.91 (d, 2H, J = 8.0 Hz), 7.88 (d, 2H, J = 9.0 Hz), 7.56–7.46 (m, 5H). ^{13}C NMR ($CDCl_3$, 101 MHz): δ 152.57, 151.08, 137.01, 131.38, 129.44, 129.24, 124.25, 123.06.

1-(4-Bromophenyl)-2-phenyldiazene (L7):

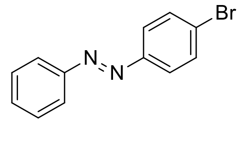

Following the general procedure gave the title compound as an orange solid (125.8 mg, 60 % isolated yield). Characterization data are consistent with previously published.[9] ^{1}H NMR ($CDCl_3$, 400 MHz): δ 7.92 (d, 2H, J = 7.6 Hz), 7.81 (d, 2H, J = 8.8 Hz), 7.65 (d, 2H, J = 8.8 Hz), 7.56–7.46 (m, 3H). ^{13}C NMR ($CDCl_3$, 101 MHz): δ 125.57, 151.43, 132.43, 131.43, 129.26, 125.49, 124.48, 123.07.

1-(4-Iodophenyl)-2-phenyldiazene (L8):

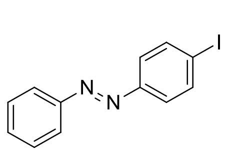

Following the general procedure gave the title compound as an orange solid (138.3 mg, 56 % isolated yield). Characterization data are consistent with previously published.[10] ^{1}H NMR ($CDCl_3$, 400 MHz): δ 7.98-7.91 (m, 4H), 7.66 (d, 2H, J = 8.6 Hz), 7.59–7.44 (m, 3H). ^{13}C NMR ($CDCl_3$, 101 MHz): δ 152.51, 151.98, 138.42, 131.44, 129.24, 124.58, 123.09, 97.81.

2.2. Regioselective mechanochemical halogenation reactions with L2–5

General procedure for the regioselective mechanochemical halogenation reactions with **L2–5**

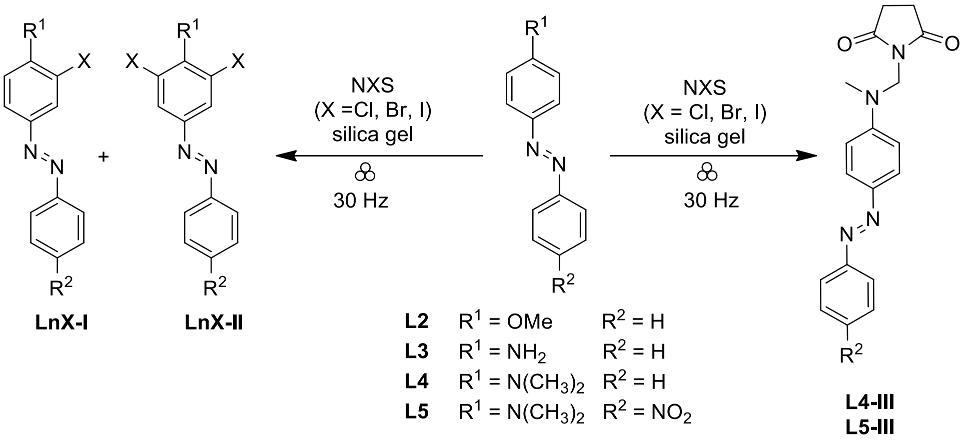

Azobenzene **L2–5** (0.5 mmol), NXS (X = Cl, Br, I) (0.6 mmol), and silica gel (250 mg) were transferred to a 14 mL polymethyl methacrylate (PMMA) jar loaded with one nickel bound tungsten carbide (WC, 7 mm in diameter, 3.9 g) milling ball. The milling jar was placed in the mixer mill operating at 30 Hz, and the reaction mixture was milled at ambient temperature (23 ± 2 °C for 1–15 hours as specified in Table 1. The crude product was scratched off from the jar, and re-chromatographed on silica gel (hexane/ethyl acetate 19:1) to obtain the desired monohalogenated product (**LnX-I**), and in the case of the reaction of **L5** with NCS and dihalogenated product (**L5Cl-II**) as a side product. NMR yield was calculated using 1,4-dinitrobenzene as an internal standard.

2-Chloro-4-(phenyldiazenyl)aniline (**L3Cl-I**):

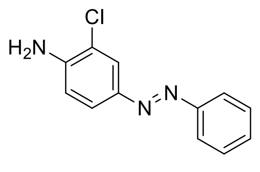

Following the general procedure gave the title compound as a brown solid (39.7 mg, 34 % isolated yield). ^{1}H NMR ($CDCl_3$, 400 MHz): δ 7.94 (d, 1H, J = 2.2 Hz, Ar-H), 7.85 (dd, 2H, J = 8.4 Hz, Ar-H), 7.75 (dd, 1H, J = 8.6 Hz, Ar-H), 7.49 (t, 2H, J = 7.4 Hz, Ar-H), 7.43 (t, 1H, J = 7.3 Hz, Ar-H), 6.85 (d, 1H, J = 8.5 Hz, Ar-H), 4.43 (s, 2H, NH_2). ^{13}C NMR ($CDCl_3$, 101 MHz): δ 152.80, 145.69, 145.56, 130.40, 129.17, 124.58, 123.82, 122.64, 119.58, 115.09. HRMS (MALDI TOF) m/z: $[M+H]^+$ Calcd for $C_{12}H_{11}ClN_3^+$ 232.0642; Found 232.0648.

2-Chloro-*N*,*N*-dimethyl-4-(phenyldiazenyl)aniline (**L4Cl-I**):

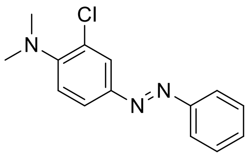

Following the general procedure gave the title compound as an orange liquid (95.3 mg, 73 % isolated yield). ^{1}H NMR ($CDCl_3$, 400 MHz): δ 7.99 (d, 1H, J = 2.3 Hz, Ar-*H*), 7.90 (dd, 2H, J = 7.9 Hz, Ar-*H*), 7.84 (dd, 1H, J = 8.6 Hz, Ar-*H*), 7.52 (t, 2H, J = 7.5 Hz, Ar-*H*), 7.46 (t, 1H, J = 7.2 Hz, Ar-*H*), 7.12 (d, 1H, J = 8.6 Hz, Ar-*H*), 2.94 (s, 6H, C*H*$_3$). ^{13}C NMR ($CDCl_3$, 101 MHz): δ 152.65, 147.60, 130.73, 129.11, 127.72, 124.21, 124.00, 122.75, 119.26, 43.47. HRMS (MALDI TOF) *m*/*z*: $[M+H]^+$ Calcd for $C_{14}H_{15}ClN_3^+$ 260.0955; Found 260.0952.

2-Chloro-*N*,*N*-dimethyl-4-((4-nitrophenyl)diazenyl)aniline (**L5Cl-I**):

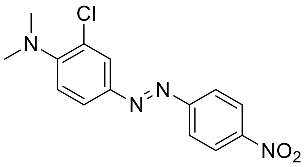

Following the general procedure gave the title compound as a red solid (110.4 mg, 72 % isolated yield). ^{1}H NMR ($CDCl_3$, 400 MHz): δ 8.37 (d, 2H, J = 9.0 Hz, Ar-*H*), 8.03-7.95 (m, 3H, Ar-*H*), 7.87 (dd, 1H, J = 8.8 Hz, Ar-*H*), 7.12 (d, 1H, J = 8.8 Hz, Ar-*H*), 2.99 (s, 6H, C*H*$_3$). ^{13}C NMR ($CDCl_3$, 101 MHz): δ 155.98, 153.89, 148.44, 147.14, 127.20, 125.05, 125.02, 124.84, 123.31, 119.08, 43.39. HRMS (MALDI TOF) *m*/*z*: $[M+H]^+$ Calcd for $C_{14}H_{14}ClN_4O_2^+$ 305.0805; Found 305.0790.

2,6-Dichloro-*N*,*N*-dimethyl-4-((4-nitrophenyl)diazenyl)aniline (**L5Cl-II**):

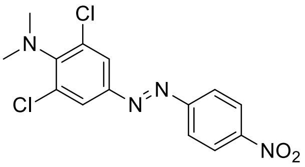

Following the general procedure gave the title compound as a red solid (13.5 mg, 8 % isolated yield). ^{1}H NMR ($CDCl_3$, 400 MHz): δ 8.36 (d, 2H, J = 9.1 Hz, Ar-*H*), 7.98 (d, 2H, J = 9.1 Hz, Ar-*H*), 7.90-7.87 (m, 2H, Ar-*H*), 3.01 (s, 6H, C*H*$_3$). ^{13}C NMR ($CDCl_3$, 101 MHz): δ 155.42, 149.93, 148.95, 148.25, 134.46, 124.88, 124.55, 123.64, 42.77. HRMS (MALDI TOF) *m*/*z*: $[M+H]^+$ Calcd for $C_{14}H_{13}Cl_2N_4O_2^+$ 339.0416; Found 339.0429.

1-(3-Bromo-4-methoxyphenyl)-2-phenyldiazene (**L2Br-I**):

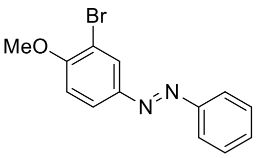

Following the general procedure gave the title compound as an orange solid (130.8 mg, 90 % isolated yield). Characterization data are consistent with previously published.[11] ^{1}H NMR ($CDCl_3$, 400 MHz): δ 8.19 (d, 1H, J = 2.4 Hz), 7.94 (dd, 1H, J = 8.8 Hz), 7.91-7.85 (m, 2H), 7.55-7.43 (m, 3H), 7.03 (d, 1H, J = 8.8 Hz), 3.99 (s, 3H). ^{13}C NMR ($CDCl_3$, 101 MHz): δ 158.10, 152.59, 147.24, 130.99, 129.23, 126.29, 125.90, 122.88, 111.48, 56.70.

2-Bromo-4-(phenyldiazenyl)aniline (**L3Br-I**):

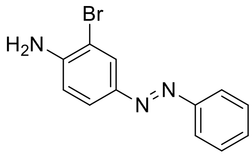

Following the general procedure gave the title compound as a brown solid (74.3 mg, 54 % isolated yield). ^{1}H NMR ($CDCl_3$, 400 MHz): δ 8.10 (d, 1H, J = 2.1 Hz, Ar-H), 7.85 (dd, 2H, J = 8.4 Hz, Ar-H), 7.79 (dd, 1H, J = 8.5 Hz, Ar-H), 7.49 (t, 2H, J = 7.6 Hz, Ar-H), 7.42 (t, 1H, J = 7.2 Hz, Ar-H), 6.85 (d, 1H, J = 8.5 Hz, Ar-H), 4.48 (s, 1H, NH_2). ^{13}C NMR ($CDCl_3$, 101 MHz): δ 152.71, 146.75, 145.64, 130.37, 129.14, 127.03, 125.08, 122.51, 114.89, 109.31. HRMS (MALDI TOF) m/z: $[M+H]^+$ Calcd for $C_{12}H_{11}BrN_3^+$ 276.0136; Found 276.0136.

2-Bromo-*N*,*N*-dimethyl-4-(phenyldiazenyl)aniline (**L4Br-I**):

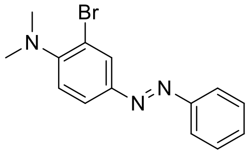

Following the general procedure gave the title compound as a brown solid (102.2 mg, 67 % isolated yield). ^{1}H NMR ($CDCl_3$, 400 MHz): δ 8.17 (d, 1H, J = 2.2 Hz, Ar-H), 7.91-7.85 (m, 3H, Ar-H), 7.51 (t, 2H, J = 7.2 Hz, Ar-H), 7.45 (t, 1H, J = 7.2 Hz, Ar-H), 7.15 (d, 1H, J = 8.6 Hz, Ar-H), 2.92 (s, 6H, CH_3). ^{13}C NMR ($CDCl_3$, 101 MHz): δ 154.24, 152.71, 148.17, 130.89, 129.21, 127.55, 124.61, 122.86, 119.85, 118.43, 44.06. HRMS (MALDI TOF) m/z: $[M+H]^+$ Calcd for $C_{14}H_{15}BrN_3^+$ 304.0449; Found 304.0448.

2-Bromo-*N*,*N*-dimethyl-4-((4-nitrophenyl)diazenyl)aniline (**L5Br-I**):

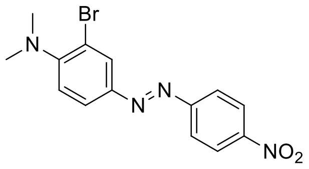

Following the general procedure gave the title compound as a purple solid (65.2 mg, 37 % isolated yield). ^{1}H NMR ($CDCl_3$, 400 MHz): δ 8.35 (d, 2H, J = 9.1 Hz, Ar-H), 8.18 (d, 1H, J = 2.3 Hz, Ar-H), 7.97 (d, 1H, J = 8.8 Hz, Ar-H), 7.13 (d, 1H, J = 8.6 Hz, Ar-H), 2.97 (s, 6H, CH_3). ^{13}C NMR ($CDCl_3$, 101 MHz): δ 155.91, 155.42, 148.50, 147.63, 128.33, 125.53, 124.85, 123.35, 119.62, 117.60, 43.85. HRMS (MALDI TOF) m/z: $[M+H]^+$ Calcd for $C_{14}H_{14}BrN_4O_2^+$ 349.0300; Found 349.0313.

2-Iodo-4-(phenyldiazenyl)aniline (**L3I-I**):

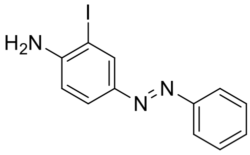

Following the general procedure gave the title compound as a brown solid (34.4 mg, 21 % isolated yield). ^{1}H NMR ($CDCl_3$, 400 MHz): δ 8.31 (d, 1H, J = 2.2 Hz, Ar-H), 7.91-7.76 (m, 3H, Ar-H), 7.50 (t, 2H, J = 7.5 Hz, Ar-H), 7.42 (t, 1H, J = 7.3 Hz, Ar-H), 6.81 (d, 1H, J = 8.6 Hz, Ar-H), 4.29 (s, 1H, NH_2). ^{13}C NMR ($CDCl_3$, 101 MHz): δ 152.75, 149.36, 146.00, 133.84, 130.37, 129.16, 125.70, 122.62, 113.85, 83.66. HRMS (MALDI TOF) m/z: $[M+H]^+$ Calcd for $C_{12}H_{11}IN_3^+$ 323.9998; Found 324.0014.

1-((Methyl(4-(phenyldiazenyl)phenyl)amino)methyl)pyrrolidine-2,5-dione (**L4-III**):

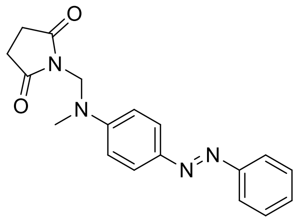

Following the general procedure gave the title compound as a dark orange solid (46.3 mg, 29 % isolated yield). ^{1}H NMR ($CDCl_3$, 400 MHz): δ 7.89 (d, 2H, J = 9.2 Hz, Ar-H), 7.85 (d, 2H, J = 8.1 Hz, Ar-H), 7.48 (t, 2H, J = 7.3 Hz, Ar-H), 7.40 (t, 1H, J = 7.3 Hz, Ar-H), 7.09 (d, 2H, J = 9.3 Hz, Ar-H), 5.17 (s, 2H, CH_2), 3.26 (s, 3H, CH_3), 2.72 (s, 4H, succ CH_2). ^{13}C NMR ($CDCl_3$, 101 MHz): δ 177.51, 153.12, 149.67, 145.18, 129.93, 129.08, 124.84, 122.48, 112.93, 56.74, 40.13, 28.31. HRMS (MALDI TOF) m/z: $[M+H]^+$ Calcd for $C_{18}H_{19}N_4O_2^+$ 323.1508; Found 323.1515.

1-((Methyl(4-((4-nitrophenyl)diazenyl)phenyl)amino)methyl)pyrrolidine-2,5-dione (**L5-III**):

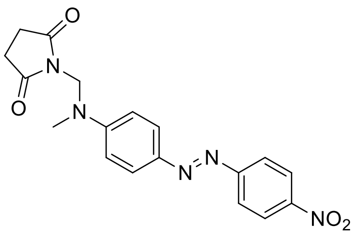

Following the general procedure gave the title compound as a dark orange solid (57.4 mg, 31 % isolated yield). ^{1}H NMR ($CDCl_3$, 400 MHz): δ 8.33 (d, 2H, J = 9.0 Hz, Ar-H), 8.0-7.89 (m, 4H, Ar-H), 7.12 (d, 2H, J = 9.3 Hz, Ar-H), 5.21 (s, 2H, CH_2), 3.31 (s, 3H, CH_3), 2.75 (s, 4H, succ CH_2). ^{13}C NMR ($CDCl_3$, 101 MHz): δ 177.50, 156.55, 150.92, 147.86, 145.17, 125.81, 124.80, 122.97, 112.96, 56.52, 40.21, 28.35. HRMS (MALDI TOF) m/z: $[M+H]^+$ Calcd for $C_{18}H_{18}N_5O_4^+$ 368.1359; Found 368.1351.

2.3. Regioselective mechanochemical halogenation reactions with L1 and L6–8

General procedure for the regioselective mechanochemical halogenation reactions with **L1** and **L6–8**

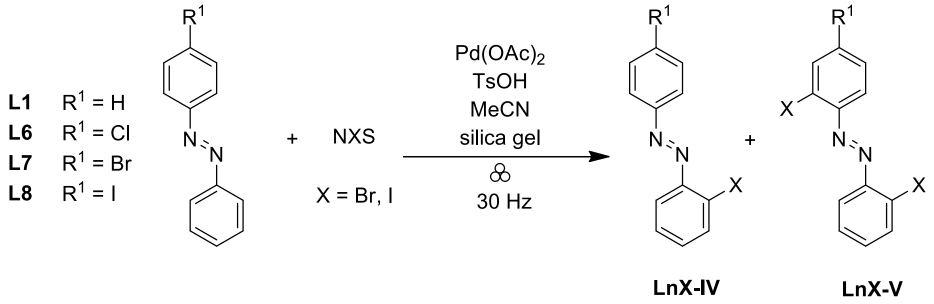

Azobenzene **L1** or **L6–8** (0.5 mmol), NXS (X = Br, I, 0.6 mmol), $Pd(OAc)_2$ (0.025 mmol), TsOH (0.25 mmol), MeCN (15 μL), and silica gel (250 mg) were transferred to a 14 mL polymethyl methacrylate (PMMA) jar loaded with one nickel bound tungsten carbide (WC, 7 mm in diameter, 3.9 g) milling ball. The milling jar was placed in the mixer mill operating at 30 Hz, and the reaction mixture was milled at the ambient temperature (23 ± 2 °C) for 4–8 hours as specified in Table 2. The crude product was scratched off from the jar, and re-chromatographed on silica gel (hexane/ethyl acetate 19:1) to obtain the desired monohalogenated product (**LnX-IV**), and in some cases dihalogenated product (**LnX-V**) as a side product. NMR yield was calculated using 1,4-dinitrobenzene as an internal standard.

1-(2-Bromophenyl)-2-(4-chlorophenyl)diazene (**L6Br-IV**):

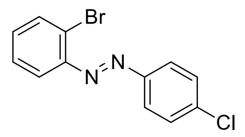

Following the general procedure gave the title compound as an orange solid (87.4 mg, 59 % isolated yield). Characterization data are consistent with previously published.[11] ^{1}H NMR ($CDCl_3$, 400 MHz): δ 7.93 (d, 2H, J = 8.7 Hz), 7.76 (d, 1H, J = 8.0 Hz), 7.67 (d, 1H, J = 8.0 Hz), 7.50 (d, 2H, J = 8.7 Hz), 7.39 (t, 1H, J = 7.6 Hz), 7.32 (t, 1H, J = 7.6 Hz). ^{13}C NMR ($CDCl_3$, 101 MHz): δ 151.09, 149.53, 137.71, 133.96, 132.29, 129.57, 128.11, 126.16, 124.78, 117.83.

1-(2-Bromophenyl)-2-(4-bromophenyl)diazene (**L7Br-IV**):

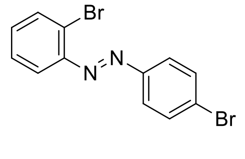

Following the general procedure gave the title compound as an orange solid (102.6 mg, 60 % isolated yield). ^{1}H NMR ($CDCl_3$, 400 MHz): δ 7.86 (d, 2H, J = 8.7 Hz, Ar-*H*), 7.76 (d, 1H, J = 8.0 Hz, Ar-*H*), 7.71-7.63 (m, 3H, Ar-*H*), 7.40 (t, 1H, J = 7.6 Hz, Ar-*H*), 7.33 (t, 1H, J = 7.6 Hz, Ar-*H*). ^{13}C NMR ($CDCl_3$, 101 MHz): δ 151.48, 149.59, 134.00, 132.59, 132.55, 132.36, 128.15, 126.26, 126.18, 125.01, 117.85. HRMS (MALDI TOF) m/z: $[M+H]^+$ Calcd for $C_{12}H_9Br_2N_2^+$ 338.9132; Found 338.9131.

1-(2-Bromophenyl)-2-(4-iodophenyl)diazene (**L8Br-IV**):

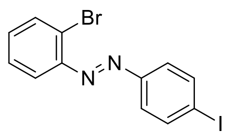

Following the general procedure gave the title compound as an orange solid (106.3 mg, 55 % isolated yield). ^{1}H NMR ($CDCl_3$, 400 MHz): δ 7.88 (d, 2H, J = 8.7 Hz, Ar-*H*), 7.76 (d, 1H, J = 7.9 Hz, Ar-*H*), 7.73-7.64 (m, 3H, Ar-*H*), 7.39 (t, 1H, J = 7.7 Hz, Ar-*H*), 7.33 (t, 1H, J = 7.7 Hz, Ar-*H*). ^{13}C NMR ($CDCl_3$, 101 MHz): δ 151.93, 149.44, 138.53, 133.93, 132.32, 128.08, 126.22, 125.02, 117.79, 98.68. HRMS (MALDI TOF) *m*/*z*: $[M+H]^+$ Calcd for $C_{12}H_9BrIN_2^+$ 386.8994; Found 386.8996.

1-(4-Chlorophenyl)-2-(2-iodophenyl)diazene (**L6I-IV**):

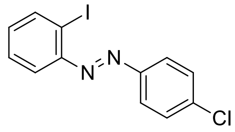

Following the general procedure gave the title compound as an orange solid (108.4 mg, 63 % isolated yield). Characterization data are consistent with previously published.[11] ^{1}H NMR ($CDCl_3$, 400 MHz): δ 8.04 (d, 1H, J = 7.7 Hz), 7.95 (d, 2H, J = 8.0 Hz), 7.64 (d, 1H, J = 7.7 Hz), 7.51 (d, 2H, J = 8.0 Hz), 7.43 (t, 1H, J = 7.7 Hz), 7.18 (t, 1H, J = 7.7 Hz). ^{13}C NMR ($CDCl_3$, 101 MHz): δ 151.19, 150.77, 140.05, 137.70, 132.60, 129.58, 129.04, 124.90, 117.41, 102.99.

1-(4-Chloro-2-iodophenyl)-2-(2-iodophenyl)diazene (**L6I-V**):

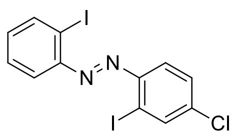

Following the general procedure gave the title compound as an orange solid (25.1 mg, 11 % isolated yield; yield calculated with respect to **L6**). ^{1}H NMR ($CDCl_3$, 400 MHz): δ 8.08-8.00 (m, 2H, Ar-*H*), 7.75 (d, 1H, J = 8.0 Hz, Ar-*H*), 7.70 (d, 1H, J = 8.6 Hz, Ar-*H*), 7.50-7.40 (m, 2H, Ar-*H*), 7.20 (t, 1H, J = 7.6 Hz, Ar-*H*). ^{13}C NMR ($CDCl_3$, 101 MHz): δ 150.85, 149.58, 140.17, 139.36, 138.27, 133.17, 129.54, 129.21, 118.88, 118.34, 103.64, 103.54. HRMS (MALDI TOF) *m*/*z*: $[M+H]^+$ Calcd for $C_{12}H_8ClI_2N_2^+$ 468.8465; Found 468.8460.

1-(2-Iodophenyl)-2-(4-bromophenyl)diazene (**L7I-IV**):

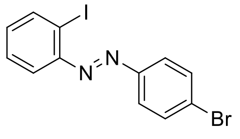

Following the general procedure gave the title compound as an orange solid (114.9 mg, 59 % isolated yield). ^{1}H NMR ($CDCl_3$, 400 MHz): δ 8.04 (d, 1H, J = 7.9 Hz, Ar-*H*), 7.87 (d, 2H, J = 8.8 Hz, Ar-*H*), 7.71-7.60 (m, 3H, Ar-*H*), 7.43 (t, 1H, J = 7.6 Hz, Ar-*H*), 7.18 (t, 1H, J = 7.6 Hz, Ar-*H*). ^{13}C NMR ($CDCl_3$, 101 MHz): δ 151.14, 151.07, 140.03, 132.62, 132.54, 129.02, 126.23, 125.08, 124.78, 117.38, 103.07. HRMS (MALDI TOF) *m*/*z*: $[M+H]^+$ Calcd for $C_{12}H_9BrIN_2^+$ 386.8994; Found 386.8995.

1-(4-Bromo-2-iodophenyl)-2-(2-iodophenyl)diazene (**L7I-V**):

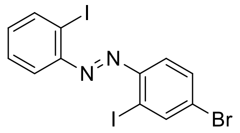

Following the general procedure gave the title compound as an orange solid (19.9 mg, 8 % isolated yield; yield calculated with respect to **L7**). ^{1}H NMR ($CDCl_3$, 400 MHz): δ 8.20 (d, 1H, J = 2.0 Hz, Ar-H), 8.04 (d, 1H, J = 8.0 Hz, Ar-H), 7.75 (d, 1H, J = 8.0 Hz, Ar-H), 7.64 (d, 1H, J = 8.6 Hz, Ar-H), 7.59 (d, 1H, J = 8.6 Hz, Ar-H), 7.45 (t, 1H, J = 7.7 Hz, Ar-H), 7.21 (t, 1H, J = 7.6 Hz, Ar-H). ^{13}C NMR ($CDCl_3$, 101 MHz): δ 150.86, 149.95, 142.08, 140.19, 133.21, 132.48, 129.22, 126.70, 119.18, 118.35, 103.88, 103.66. HRMS (MALDI TOF) m/z: $[M+H]^+$ Calcd for $C_{12}H_8BrI_2N_2^+$ 512.7960; Found 512.7957.

1-(2-Iodophenyl)-2-(4-iodophenyl)diazene (**L8I-IV**):

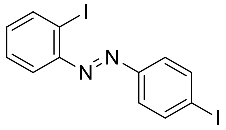

Following the general procedure gave the title compound as an orange solid (99.3 mg, 45 % isolated yield). ^{1}H NMR ($CDCl_3$, 400 MHz): δ 8.03 (d, 1H, J = 7.8 Hz, Ar-H), 7.89 (d, 2H, J = 8.6 Hz, Ar-H), 7.72 (d, 2H, J = 8.6 Hz, Ar-H), 7.63 (d, 1H, J = 7.8 Hz, Ar-H), 7.43 (t, 1H, J = 7.8 Hz, Ar-H), 7.18 (t, 1H, J = 7.8 Hz, Ar-H). ^{13}C NMR ($CDCl_3$, 101 MHz): δ 151.70, 151.18, 140.06, 138.61, 132.67, 129.05, 125.19, 117.42, 103.07, 98.70. HRMS (MALDI TOF) m/z: $[M+H]^+$ Calcd for $C_{12}H_9I_2N_2^+$ 434.8855; Found 434.8860.

1-(2,4-Diiodophenyl)-2-(2-iodophenyl)diazene (**L8I-V**):

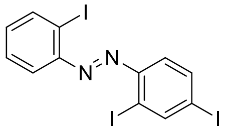

Following the general procedure gave the title compound as an orange solid (46.8 mg, 17 % isolated yield; yield calculated with respect to **L8**). ^{1}H NMR ($CDCl_3$, 400 MHz): δ 8.40 (s, 1H, Ar-H), 8.04 (d, 1H, J = 8.0 Hz, Ar-H), 7.78 (d, 1H, J = 8.6 Hz, Ar-H), 7.75 (d, 1H, J = 8.2 Hz, Ar-H), 7.51-7.41 (m, 2H, Ar-H), 7.20 (t, 1H, J = 7.5 Hz, Ar-H). ^{13}C NMR ($CDCl_3$, 101 MHz): δ 150.84, 150.57, 147.75, 140.18, 138.42, 133.23, 129.22, 119.38, 118.35, 104.30, 103.69, 99.15. HRMS (MALDI TOF) m/z: $[M+H]^+$ Calcd for $C_{12}H_8I_3N_2^+$ 560.7822; Found 560.7809.

3. Mechanistic studies

3.1. Synthesis of I6-I

Solution synthesis of **I6-I**

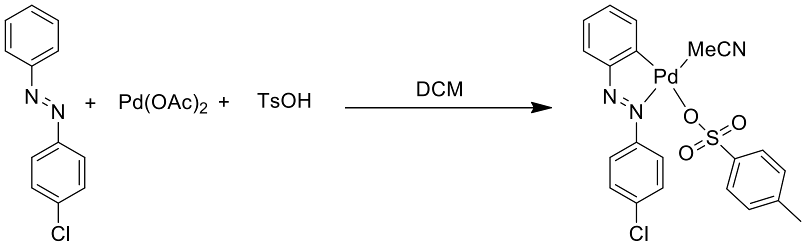

4-Chloroazobenzene (**L6**, 0.825 mmol) was added to a solution of palladium acetate (0.625 mmol) in DCM (3 mL), and the reaction mixture was stirred for 10 minutes at ambient temperature, after which a solution of TsOH (0.7 mmol) in DCM (3 mL) was added. The reaction mixture was stirred for 1 hour at ambient temperature, filtered through a thin layer of Celite, and washed with DCM. The mother liquid was collected and the solvent was evaporated. The remaining solid was dissolved in MeCN (1,5 mL), and the desired product began to precipitate as a yellow powder after successive addition of hexane followed by diethyl ether. The precipitate was filtered off, then washed with hexane, and dried under vacuum. The product **I6-I** was isolated as an orange powder (283.1 mg, 85% isolated yield). The crystal structure of **I6-I** was solved using powder X-ray diffraction.

^{1}H NMR (DMSO-d_6, 400 MHz): δ 8.06 (dd, J = 7.1 Hz, 1H, AB Ar-H), 7.88-7.76 (m, 2H, AB Ar-H), 7.71 (d, J = 8.6 Hz, 1H, AB Ar-H), 7.65 (d, J = 5.4 Hz, 1H, AB Ar-H), 7.55-7.23 (m, 5H, TsOH Ar-H and AB Ar-H), 7.10 (d, J = 7.9 Hz, 2H, TsOH Ar-H), 2.28 (s, 3H, TsOH CH_3), 2.07 (s, 3H, MeCN CH_3).

^{13}C NMR (DMSO-d_6, 101 MHz): δ 163.31, 162.08, 153.79, 153.28, 150.74, 149.24, 145.04, 137.99, 136.44, 136.17, 132.62, 132.60, 131.81, 131.57, 130.99, 130.29, 129.02, 128.94, 128.13, 127.19, 127.07, 125.51, 125.25, 123.34, 118.17, 20.82, 1.20.

Additional signals in the ^{13}C NMR spectrum suggest the existence of isomers of **I6-I** in DMSO-d_6. Only signals for the major isomer are presented in ^{1}H NMR data. Signals for the minor isomer are overlapped with signals of the major isomer of **I6-I**.

Mechanochemical synthesis of **I6-I**

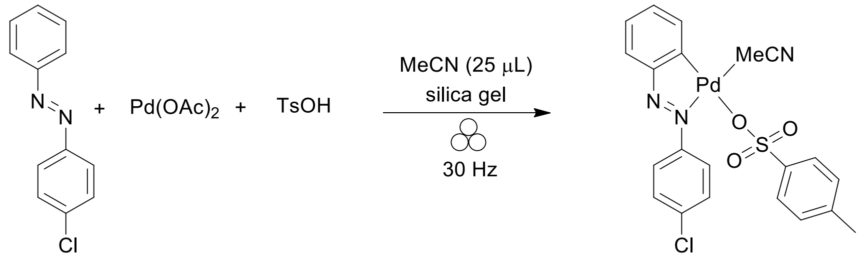

4-Chloroazobenzene (**L6**, 0.4 mmol), palladium acetate (0.42 mmol), TsOH (0.42 mmol), MeCN (25 μL), and silica gel (400 mg) were transferred to a 14 mL polymethyl methacrylate (PMMA) jar loaded with one nickel bound tungsten carbide (WC, 7 mm in diameter, 3.9 g) milling ball. The milling jar was placed in the mixer mill operating at 30 Hz, and the reaction mixture was milled at ambient temperature (23 ± 2 °C) for 3 hours. The resulting reaction mixture was scratched off from the jar, and the crude product was suspended in DCM (10 mL), and filtered through a thin layer of Celite. The mother liquid was collected, and the solvent was evaporated. The remaining solid was dissolved in MeCN (3 mL), and the desired product began to precipitate as a yellow powder after successive addition of hexane followed by diethyl ether. The precipitate was filtered, then washed with hexane, and dried under vacuum. The product **I6-I** was isolated as an orange powder (46.2 mg, 22% isolated yield). Characterization data are in agreement with those obtained for the same compound synthesized by the solution procedure.

3.2. Synthesis of I7-I

Solution synthesis of **I7-I**

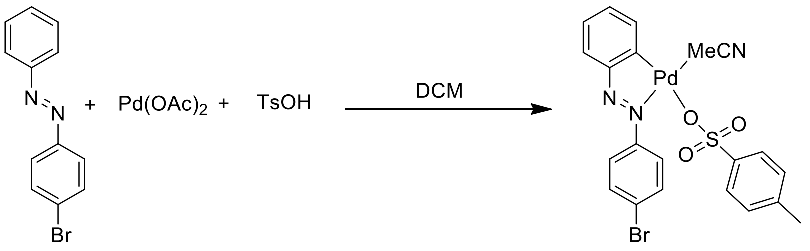

L7 **I7-I**

4-Bromoazobenzene (**L7**, 0.825 mmol) was added to a solution of palladium acetate (0.625 mmol) in DCM (3 mL), and the reaction mixture was stirred for 10 minutes at ambient temperature, after which a solution of TsOH (0.7 mmol) in DCM (3 mL) was added. The reaction mixture was stirred for 1 hour at ambient temperature, filtered through a thin layer of Celite, and washed with DCM. The mother liquid was collected and the solvent was evaporated. The remaining solid was dissolved in MeCN (1,5 mL), and the desired product began to precipitate as a yellow powder after successive addition of hexane followed by diethyl ether. The precipitate was filtered off, then washed with hexane, and dried under vacuum. The product **I7-I** was isolated as an orange powder (201.6 mg, 56% isolated yield). The crystal structure of **I7-I** was solved using powder X-ray diffraction.

^{1}H NMR (DMSO-d_6, 400 MHz): δ 8.05 (d, J = 7.3 Hz, 1H, AB Ar-*H*), 7.85 (d, J = 8.5 Hz, 2H, AB Ar-*H*), 7.76 (d, J = 8.6 Hz, 2H, AB Ar-*H*), 7.65 (m, 1H, AB Ar-*H*), 7.50-7.26 (m, 5H, TsOH Ar-*H* and AB Ar-*H*), 7.10 (d, J = 7.8 Hz, 2H, TsOH Ar-*H*), 2.29 (s, 3H, TsOH CH_3), 2.07 (s, 3H, MeCN CH_3).

^{13}C NMR (DMSO-d_6, 101 MHz): δ 163.34, 162.22, 153.93, 153.29, 150.81, 149.59, 144.83, 138.11, 134.43, 132.63, 131.85, 131.19, 130.31, 130.15, 129.01, 128.18, 127.09, 126.29, 125.53, 125.40, 125.03, 123.33, 118.23, 20.86, 1.23.

Additional signals in the ^{13}C NMR spectrum suggest the existence of isomers of **I7-I** in DMSO-d_6. Only signals for major isomer are presented in ^{1}H NMR data. Signals for the minor isomer are overlapped with signals of the major isomer of **I7-I**.

Mechanochemical synthesis of **I7-I**

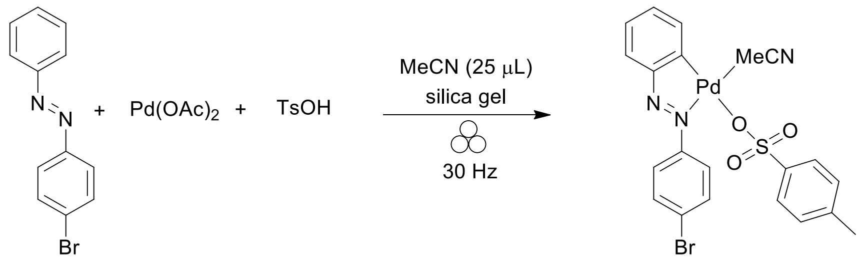

L7 **I7-I**

4-Bromoazobenzene (**L7**, 0.4 mmol), palladium acetate (0.42 mmol), TsOH (0.42 mmol), MeCN (25 μL), and silica gel (400 mg) were transferred to a 14 mL polymethyl methacrylate (PMMA) jar loaded with one nickel bound tungsten carbide (WC, 7 mm in diameter, 3.9 g) milling ball. The milling jar was placed in the mixer mill operating at 30 Hz, and the reaction mixture was milled at ambient temperature (23 ± 2 °C) for 3 hours. The resulting reaction mixture was scratched off from the jar, and the crude product was suspended in DCM (10 mL), and filtered through a thin layer of Celite. The mother liquid was collected, and the solvent was evaporated. The remaining solid was dissolved in MeCN (3 mL), and the desired product began to precipitate as a yellow powder after successive addition of hexane followed by diethyl ether. The precipitate was filtered, then washed with hexane, and dried under vacuum. The product **I7-I** was isolated as an orange powder (50.0 mg, 13.8% isolated yield). Characterization data are in agreement with those obtained for the same compound synthesized by the solution procedure.

3.3. *In situ* observation of I6-I – I8-I during the time-resolved Raman monitoring

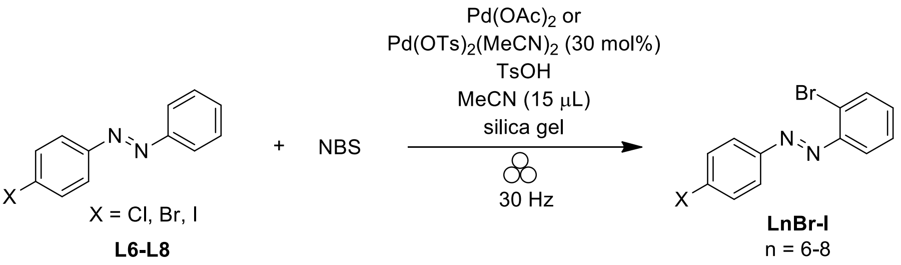

4-Halogenated azobenzene **L6–8** (0.5 mmol), NBS (0.6 mmol), $Pd(OAc)_2$ or $Pd(OTs)_2(MeCN)_2$ (0.15 mmol), TsOH (0.25 mmol), MeCN (15 μL), and silica gel (250 mg) were transferred to a 14 mL polymethyl methacrylate (PMMA) jar loaded with one nickel bound tungsten carbide (WC, 7 mm in diameter, 3.9 g) milling ball. The milling jar was placed in the mixer mill operating at 30 Hz, and the reaction mixture was milled at ambient temperature (23 ± 2 °C) for 4 hours in the case of **L6** and 8 hours in the case of **L7** and **L8**. Experimental data were interpreted by *in situ* collected Raman spectra and NMR spectra of the crude reaction mixture. *In situ* Raman monitorings of these reactions are shown in Figure 4a and Figures S23–S27.

3.4. Mechanochemical formation of I6-I and I7-I

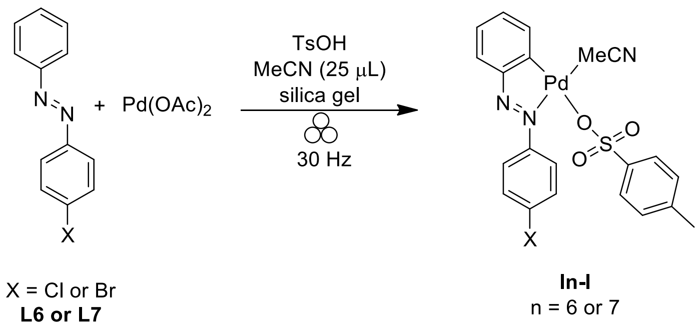

4-Chloroazobenzene **L6** or 4-bromoazobenzene **L7** (0.4 mmol), palladium acetate (0.42 mmol), TsOH (0.42 mmol), MeCN (25 μL), and silica gel (400 mg) were transferred to a 14 mL polymethyl methacrylate (PMMA) jar loaded with one nickel bound tungsten carbide (WC, 7 mm in diameter, 3.9 g) milling ball. The milling jar was placed in the mixer mill operating at 30 Hz, and the reaction mixture was milled at ambient temperature (23 ± 2 °C) for 3 hours. Experimental data were interpreted by analyzing *in situ* collected Raman spectra and NMR spectra of the crude reaction mixture. *In situ* Raman monitorings of these reactions are shown in Figure 4b and Figure S28.

3.5. Competition experiments

General procedure for competition experiments between azobenzenes **L2–5**

Azobenzene **A** (0.75 mmol), azobenzene **B** (0.75 mmol), NXS (X = Cl, Br or I, 0.5 mmol), and silica gel (250 mg) were transferred to a 14 mL polymethyl methacrylate (PMMA) jar loaded with one nickel bound tungsten carbide (WC, 7 mm in diameter, 3.9 g) milling ball. The milling jar was placed in the mixer mill operating at 30 Hz, and the reaction mixture was milled at ambient temperature (23 ± 2 °C) until full consumption of NXS was observed. The ratio of products was determined from the crude reaction mixture by ^{1}H NMR spectroscopy.

- Competition experiment between **L4** and **L3** with NCS

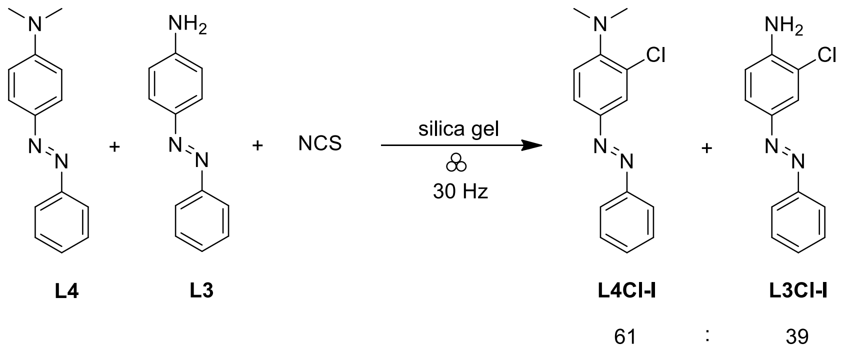

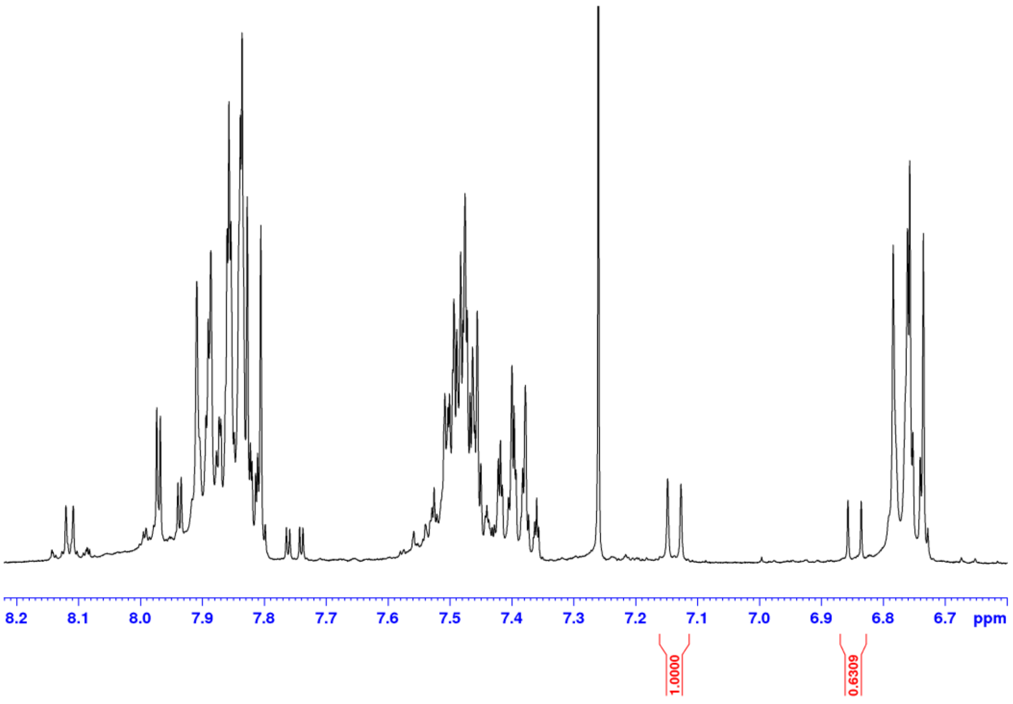

Figure S1: Aromatic region of the ^{1}H NMR spectrum of the crude reaction mixture of the competition experiment between **L4** and **L3** with NCS in $CDCl_3$ (400 MHz).

- Competition experiment between **L3** and **L5** with NCS

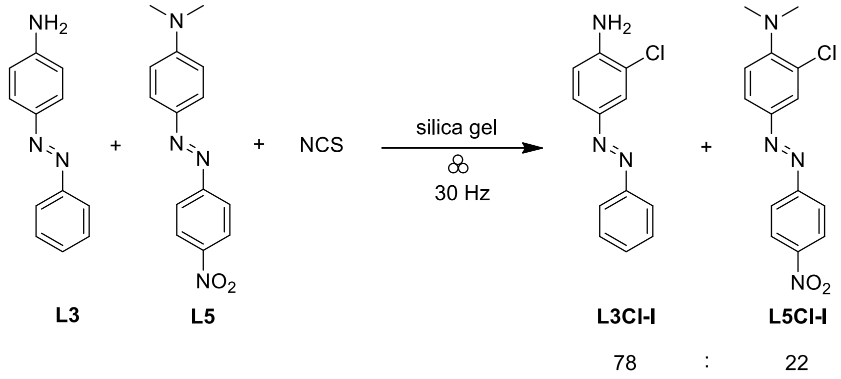

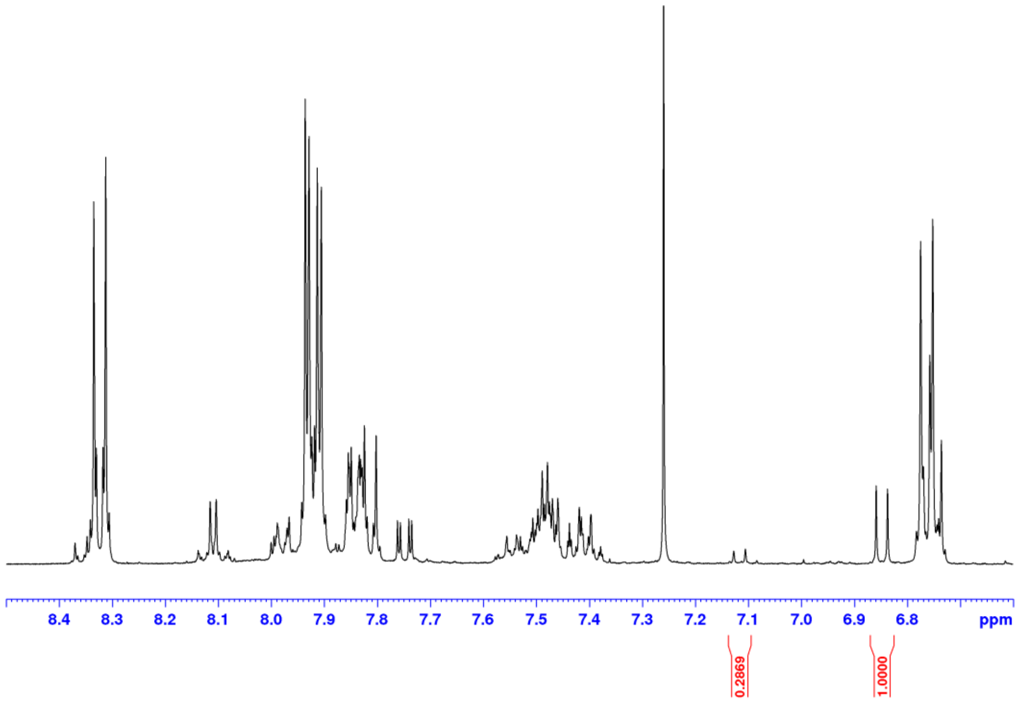

Figure S2: Aromatic region of the ^{1}H NMR spectrum of the crude reaction mixture of the competition experiment between **L3** and **L5** with NCS in $CDCl_3$ (400 MHz).

- Competition experiment between **L4** and **L2** with NBS

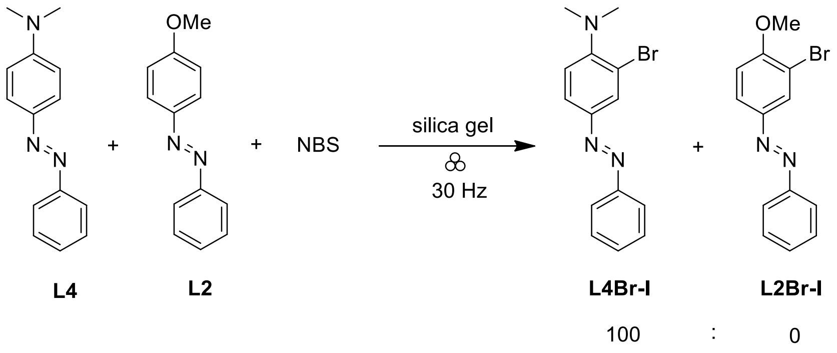

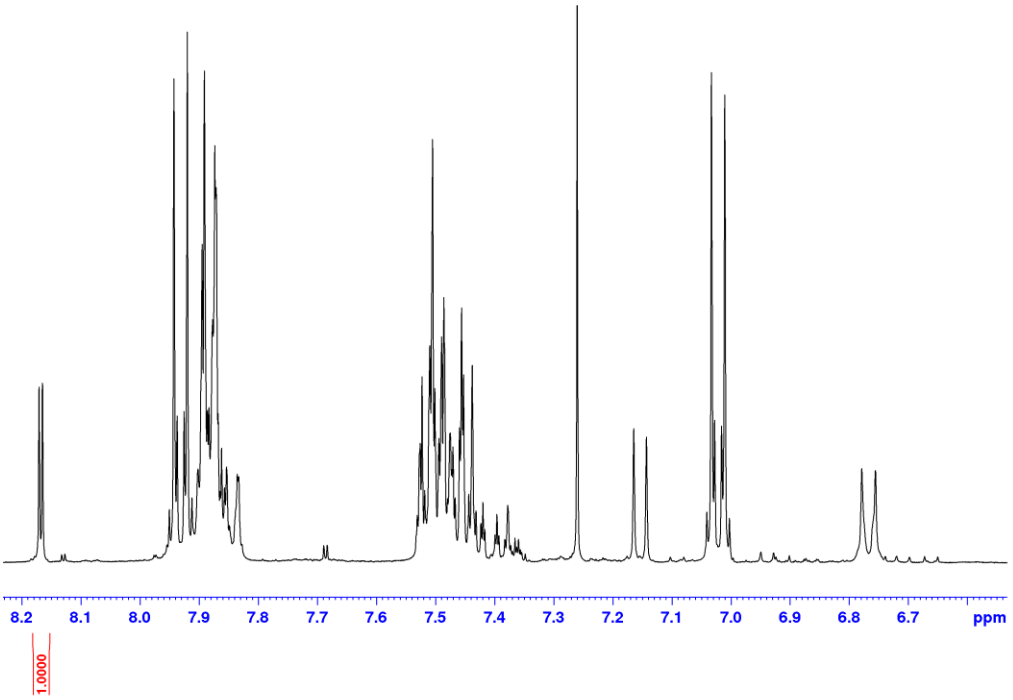

Figure S3: Aromatic region of the ^{1}H NMR spectrum of the crude reaction mixture of the competition experiment between **L4** and **L2** with NBS in $CDCl_3$ (400 MHz).

- Competition experiment between **L4** and **L3** with NBS

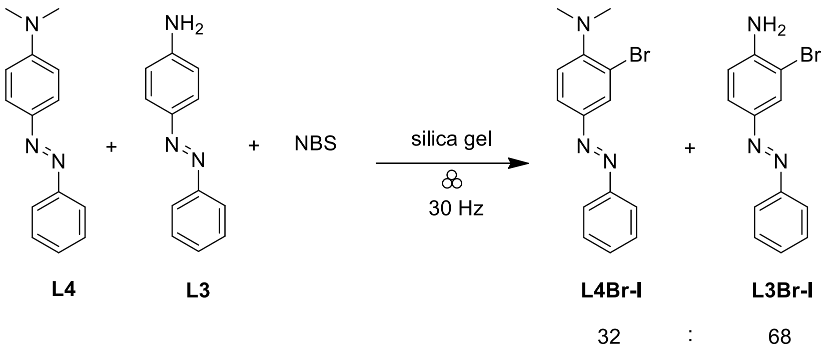

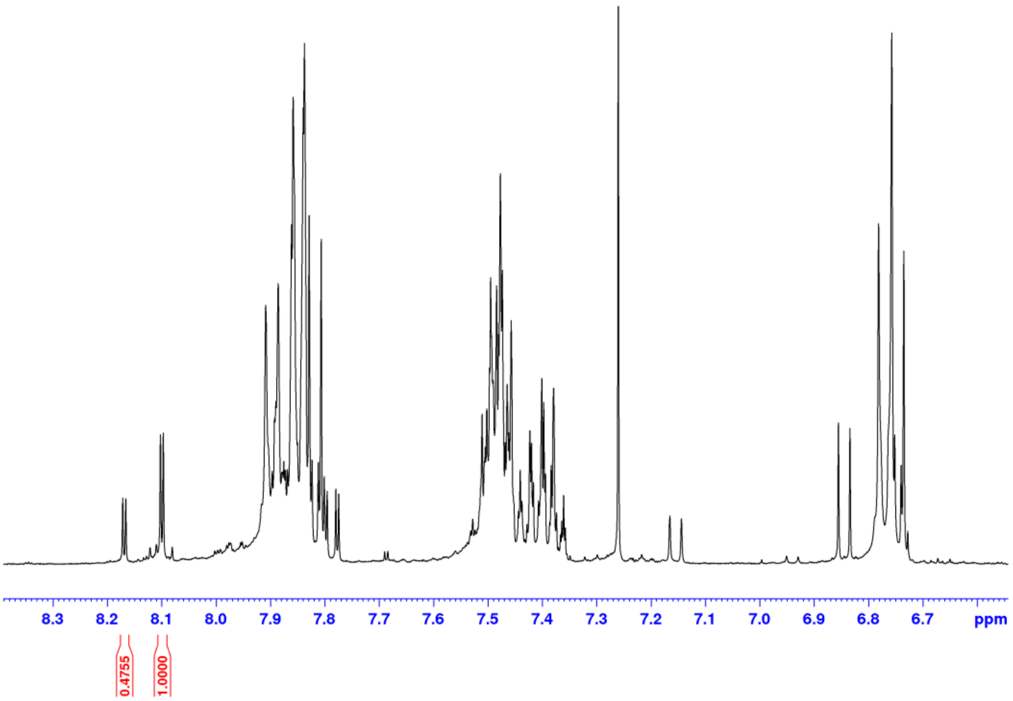

Figure S4: Aromatic region of the ^{1}H NMR spectrum of the crude reaction mixture of the competition experiment between **L4** and **L3** with NBS in $CDCl_3$ (400 MHz).

- Competition experiment between **L3** and **L5** with NBS

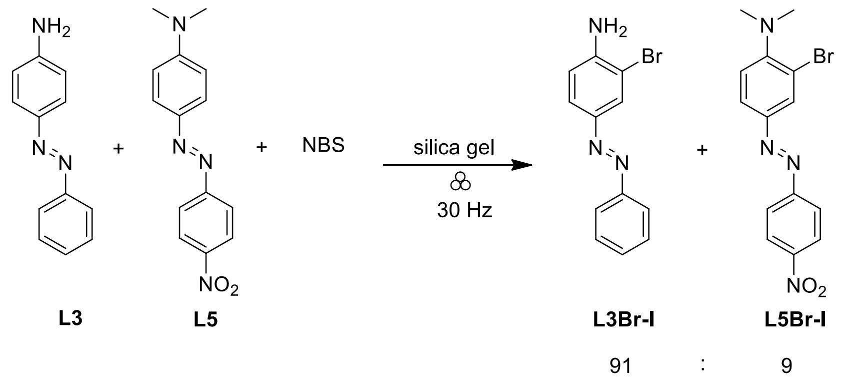

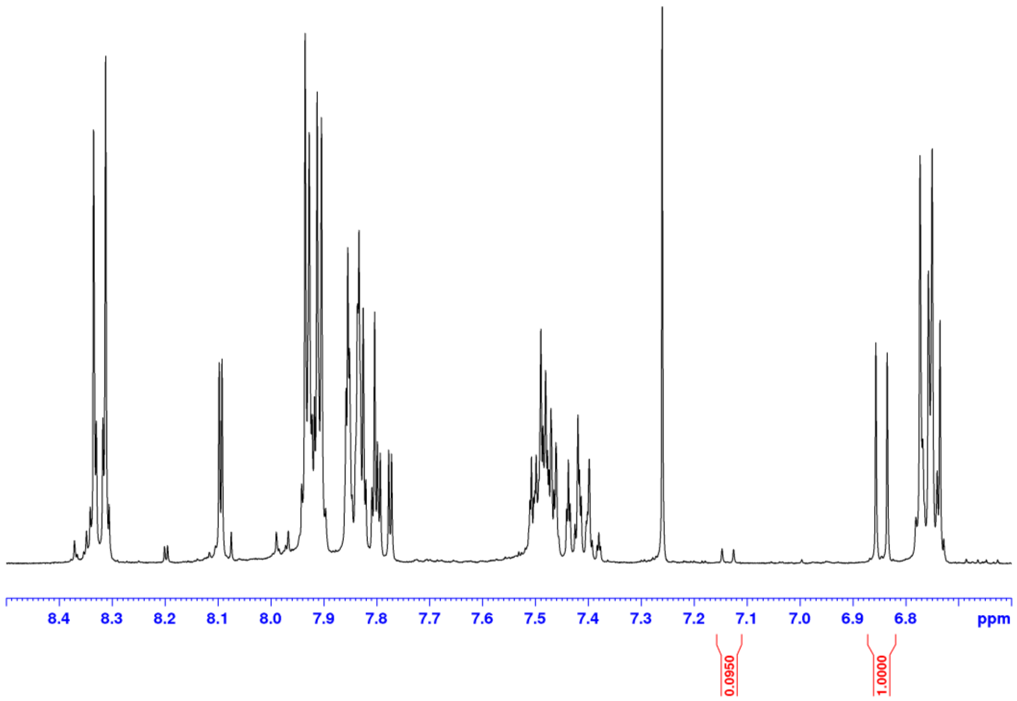

Figure S5: Aromatic region of the ^{1}H NMR spectrum of the crude reaction mixture of the competition experiment between **L3** and **L5** with NBS in $CDCl_3$ (400 MHz).

- Competition experiment between **L5** and **L2** with NBS

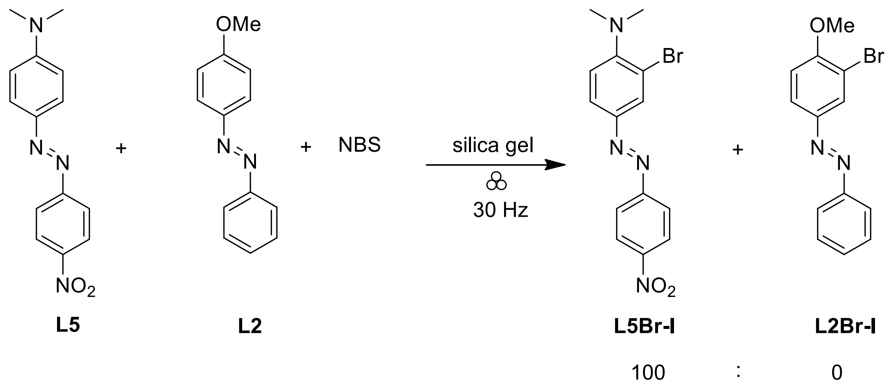

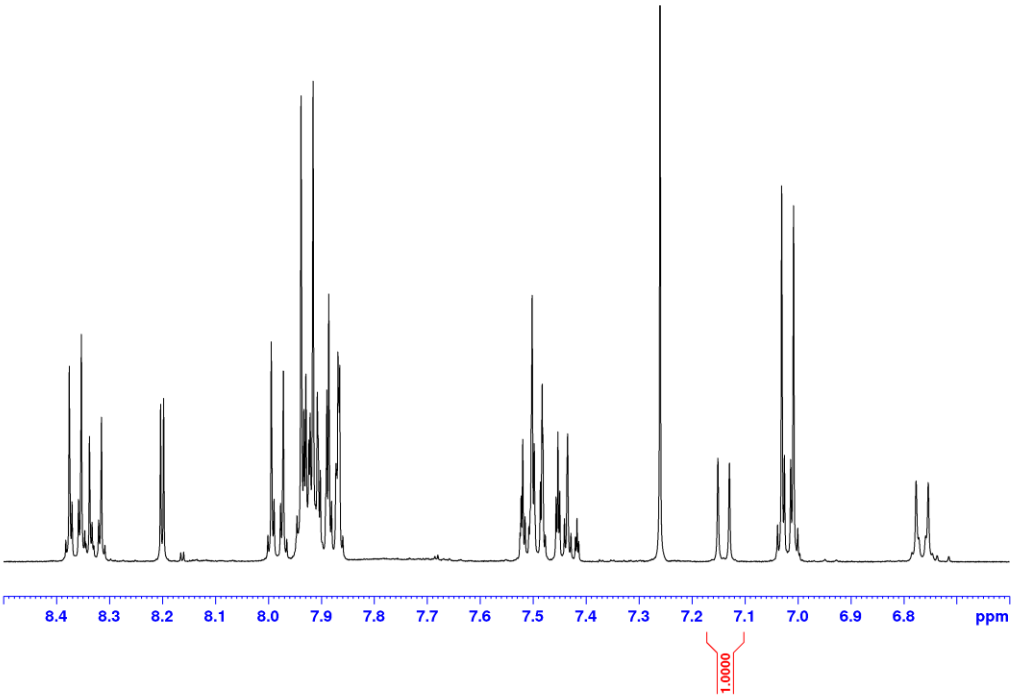

Figure S6: Aromatic region of the ¹H NMR spectrum of the crude reaction mixture of the competition experiment between **L5** and **L2** with NBS in $CDCl_3$ (400 MHz).

- Competition experiment between **L5** and **L4** with NIS

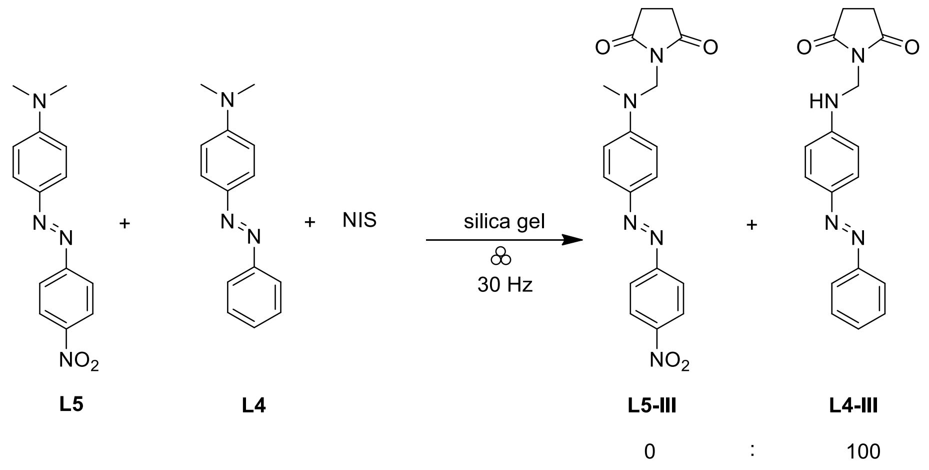

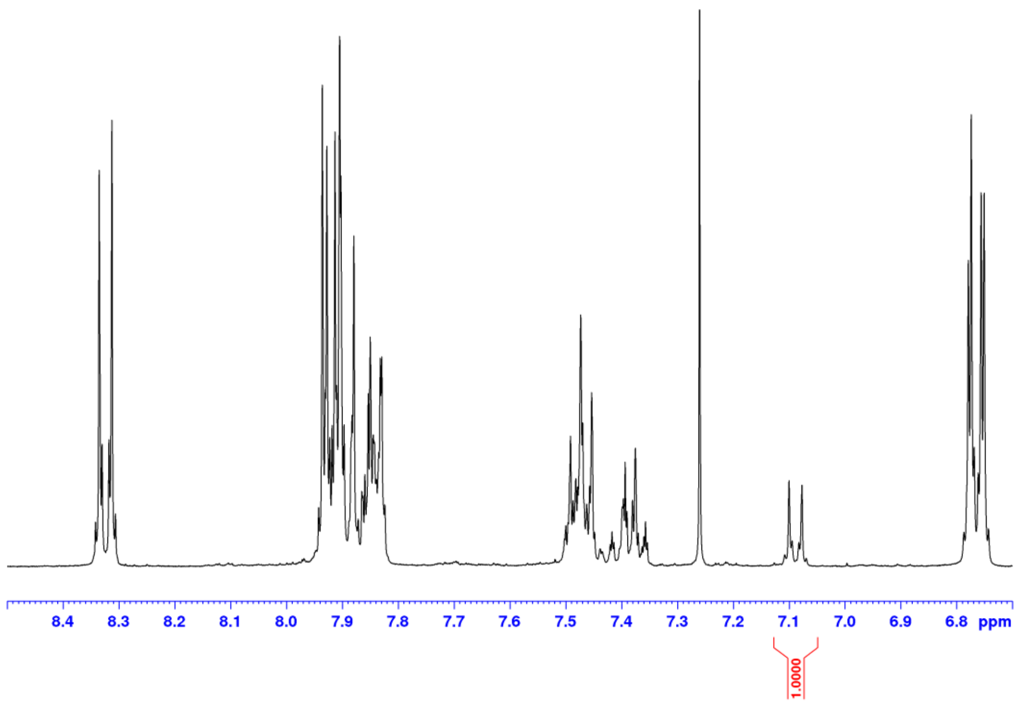

Figure S7: Aromatic region of the ^{1}H NMR spectrum of the crude reaction mixture of the competition experiment between **L5** and **L4** with NIS in $CDCl_3$ (400 MHz).

General procedure for competition experiments between azobenzenes **L1** and **L6–8**

Azobenzene **L1** (0.75 mmol), 4-halogenated azobenzene **L6–8** (0.75 mmol), NXS (X = Br or I, 0.5 mmol), $Pd(OAc)_2$ (0.025 mmol), TsOH (0.25 mmol), MeCN (15 µL), and silica gel (250 mg) were transferred to a 14 mL polymethyl methacrylate (PMMA) jar loaded with one nickel bound tungsten carbide (WC, 7 mm in diameter, 3.9 g) milling ball. The milling jar was placed in the mixer mill operating at 30 Hz, and the reaction mixture was milled at ambient temperature (23 ± 2 °C) for 5 hours. The ratio of products was determined from the crude reaction mixture by ^{1}H NMR spectroscopy.

- Competition experiment between **L1** and **L6** with NBS

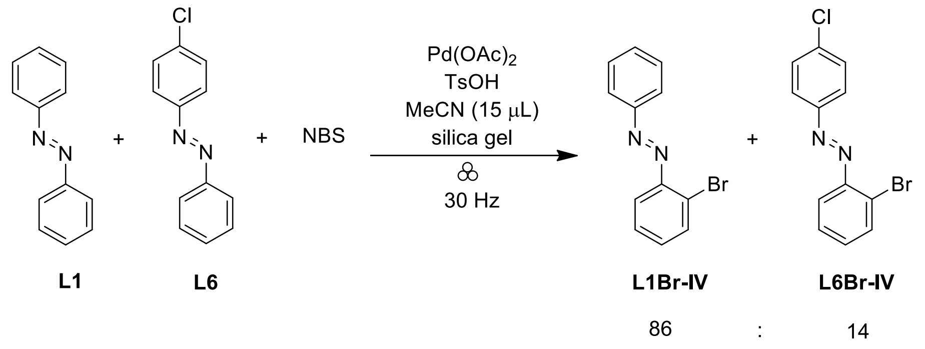

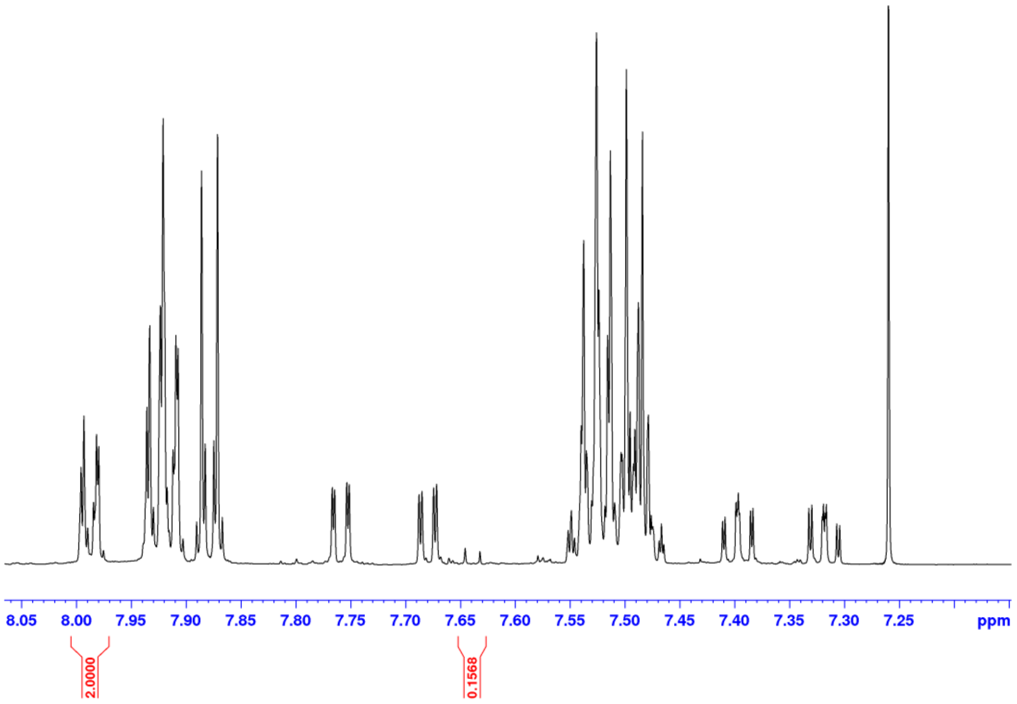

Figure S8: Aromatic region of the ^{1}H NMR spectrum of the crude reaction mixture of the competition experiment between **L1** and **L6** with NBS in $CDCl_3$ (400 MHz).

- Competition experiment between **L1** and **L7** with NBS

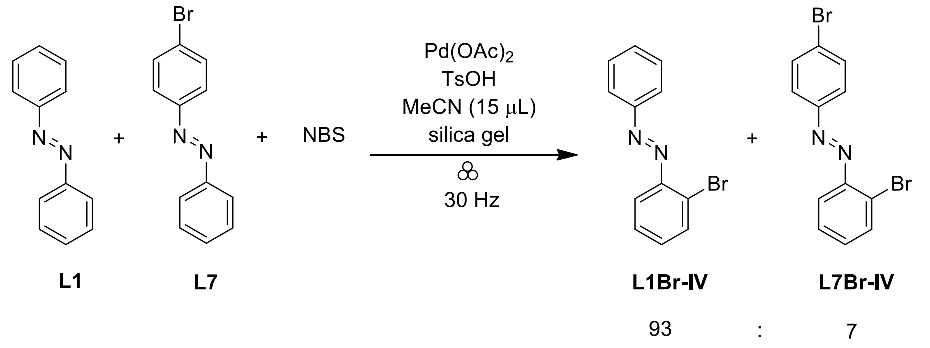

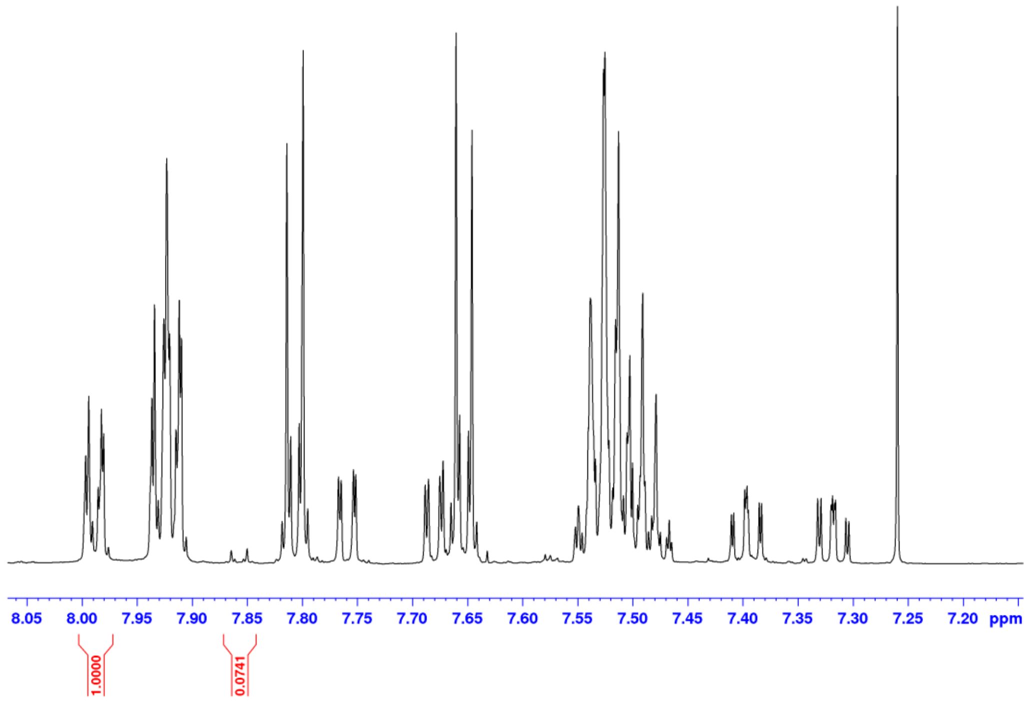

Figure S9: Aromatic region of the ^{1}H NMR spectrum of the crude reaction mixture of the competition experiment between **L1** and **L7** with NBS in $CDCl_3$ (400 MHz).

- Competition experiment between **L1** and **L8** with NBS

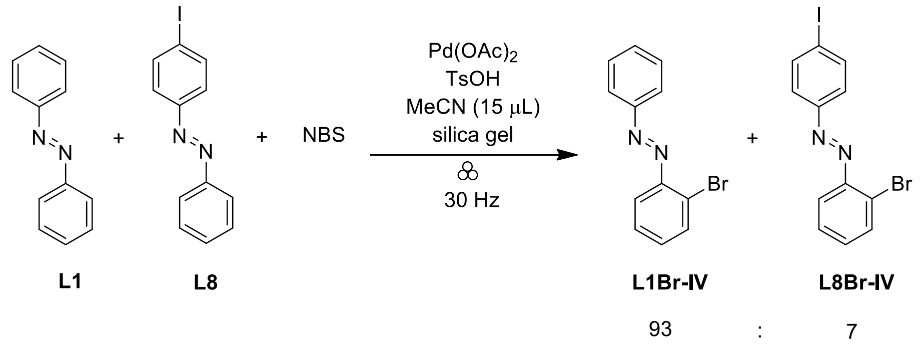

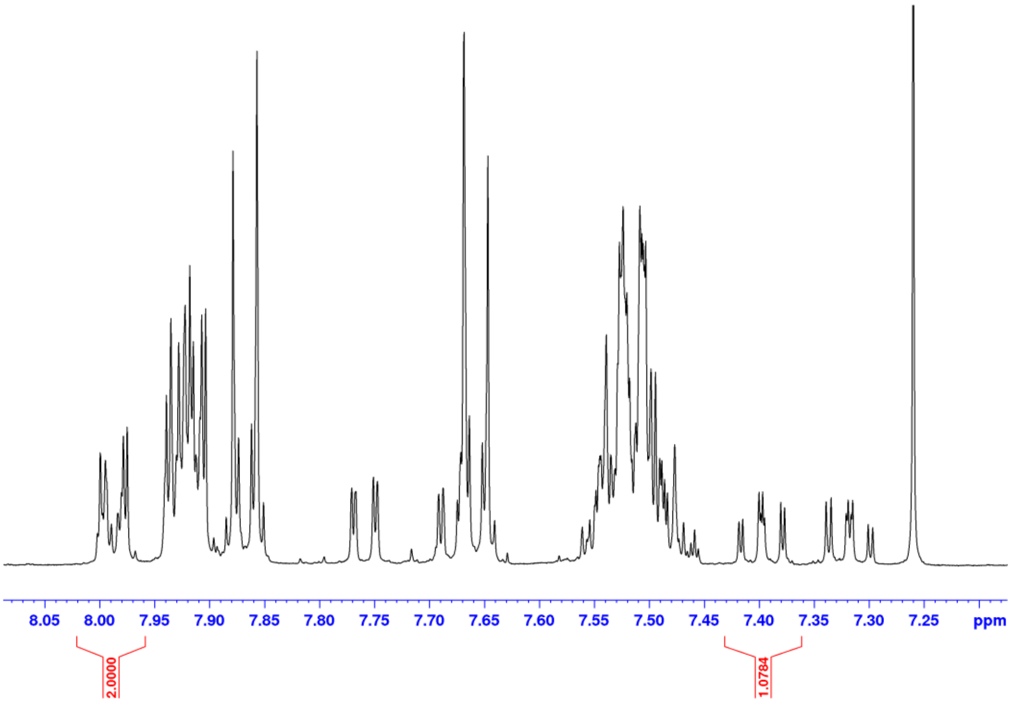

Figure S10: Aromatic region of the ^{1}H NMR spectrum of the crude reaction mixture of the competition experiment between **L1** and **L8** with NBS in $CDCl_3$ (400 MHz). Overlapping of the characteristic signals of **L1** and **L8**.

- Competition experiment between **L1** and **L6** with NIS

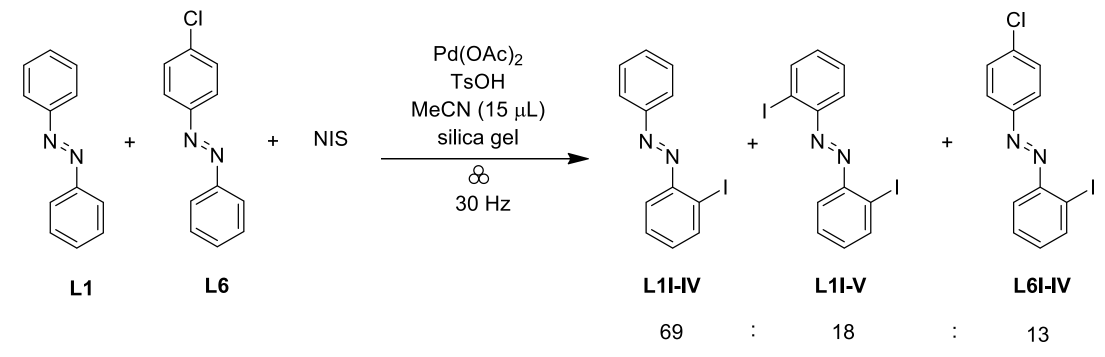

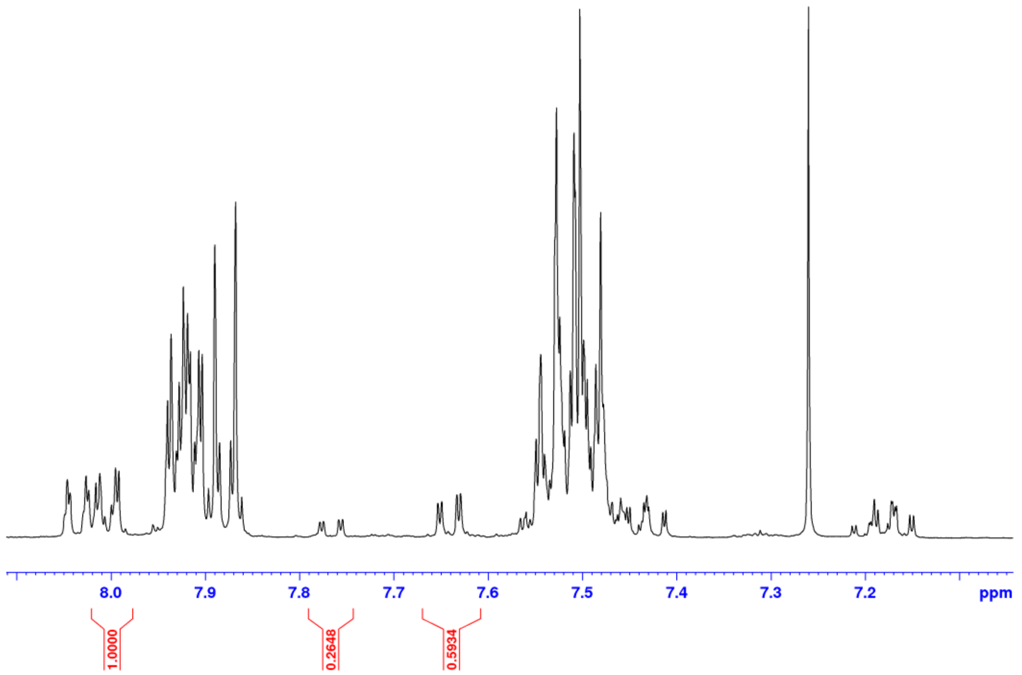

Figure S11: Aromatic region of the ^{1}H NMR spectrum of the crude reaction mixture of the competition experiment between **L1** and **L6** with NIS in $CDCl_3$ (400 MHz).

- Competition experiment between **L1** and **L7** with NIS

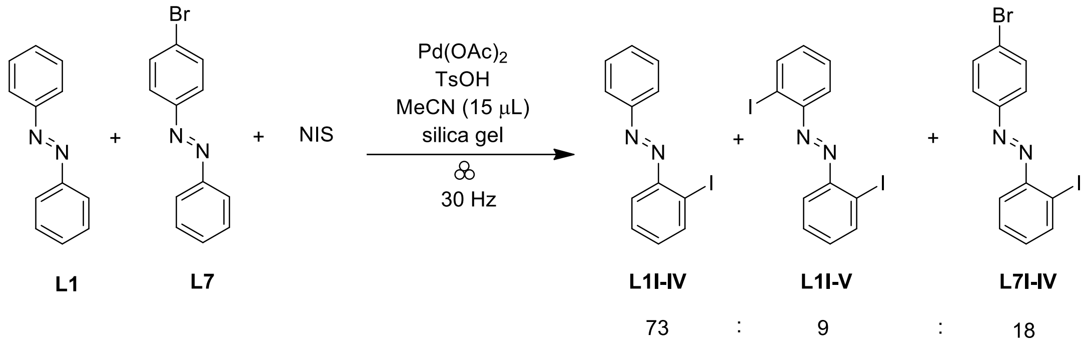

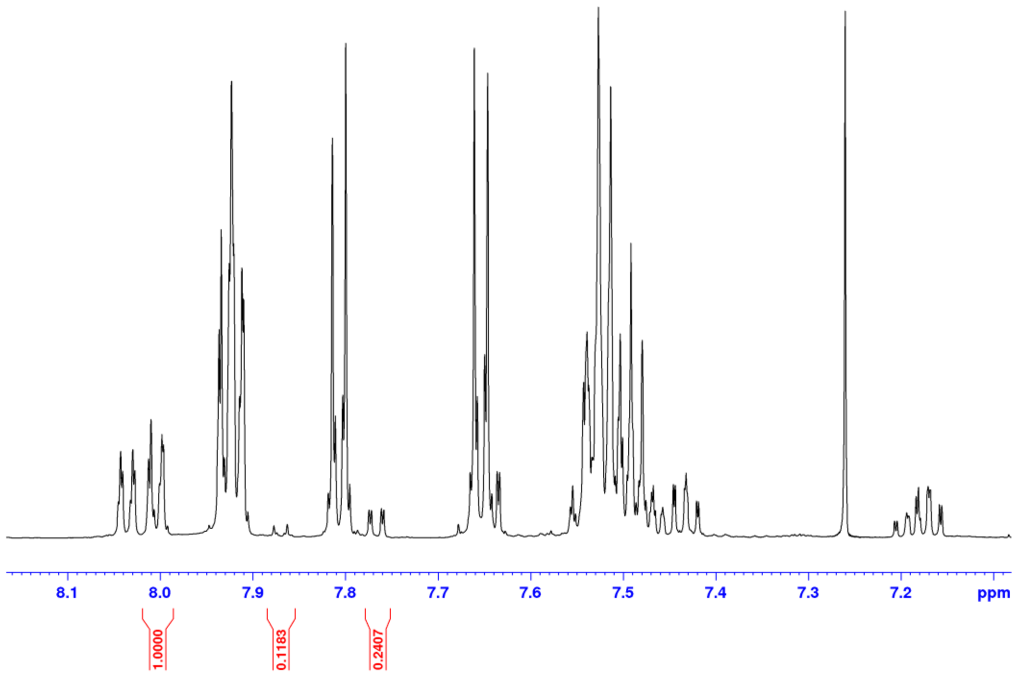

Figure S12: Aromatic region of the ^{1}H NMR spectrum of the crude reaction mixture of the competition experiment between **L1** and **L7** with NIS in $CDCl_3$ (400 MHz).

- Competition experiment between **L1** and **L8** with NIS

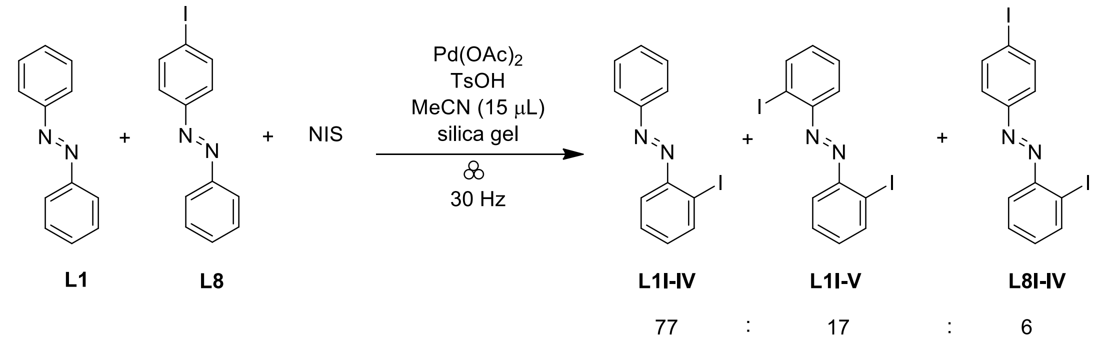

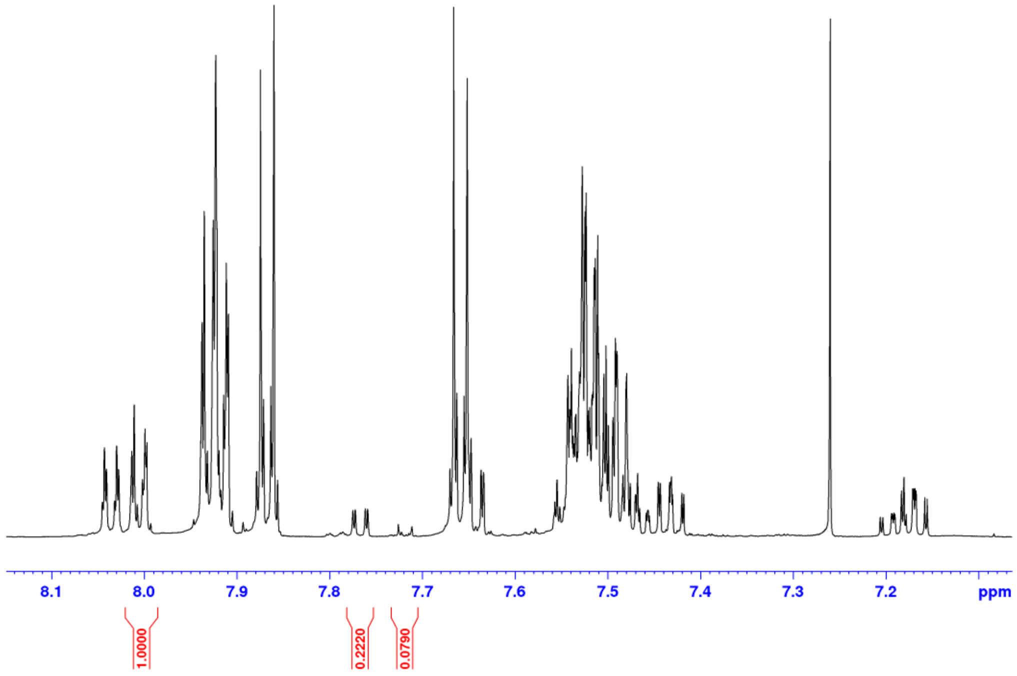

Figure S13: Aromatic region of the ^{1}H NMR spectrum of the crude reaction mixture of the competition experiment between **L1** and **L8** with NIS in $CDCl_3$ (400 MHz).

4. Raman monitoring

4.1. Halogenation of L3–5

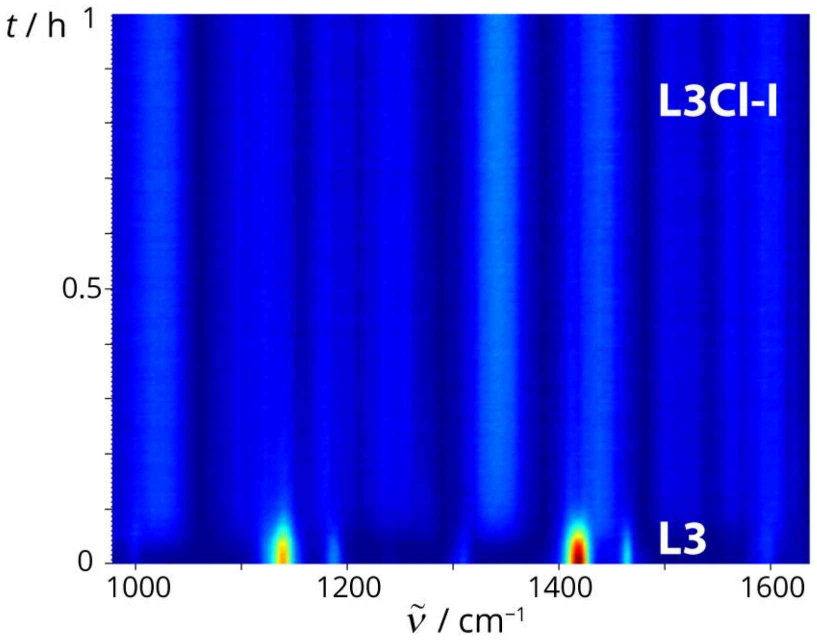

Figure S14: 2D plot of the time-resolved Raman monitoring of NG of **L3** (0.50 mmol) with NCS (0.60 mmol) and SiO_2 (250 mg).

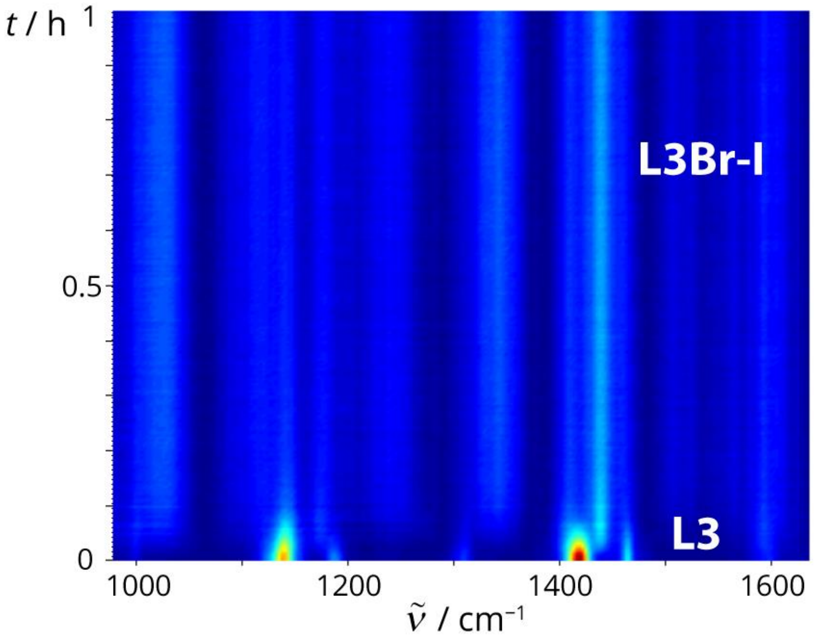

Figure S15: 2D plot of the time-resolved Raman monitoring of NG of **L3** (0.50 mmol) with NBS (0.60 mmol) and SiO_2 (250 mg).

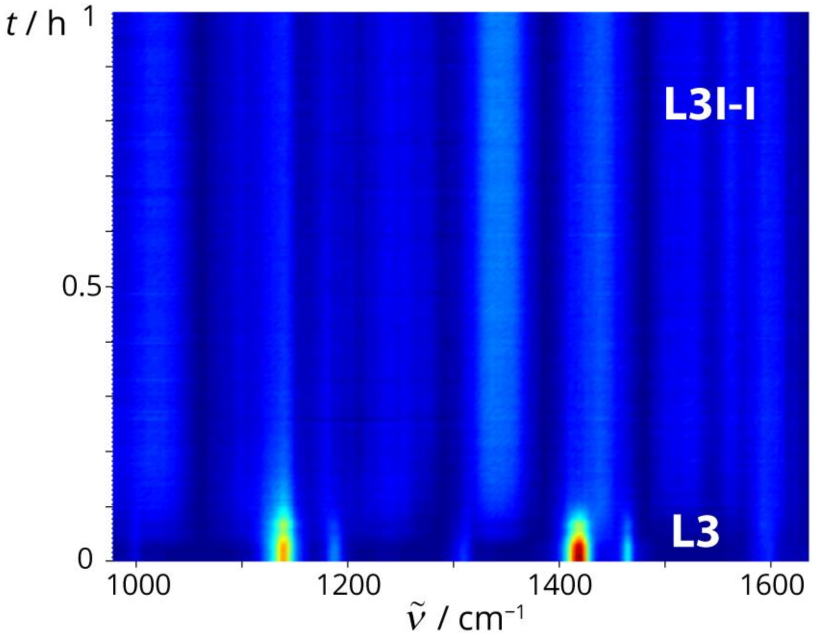

Figure S16: 2D plot of the time-resolved Raman monitoring of NG of **L3** (0.50 mmol) with NIS (0.60 mmol) and SiO_2 (250 mg).

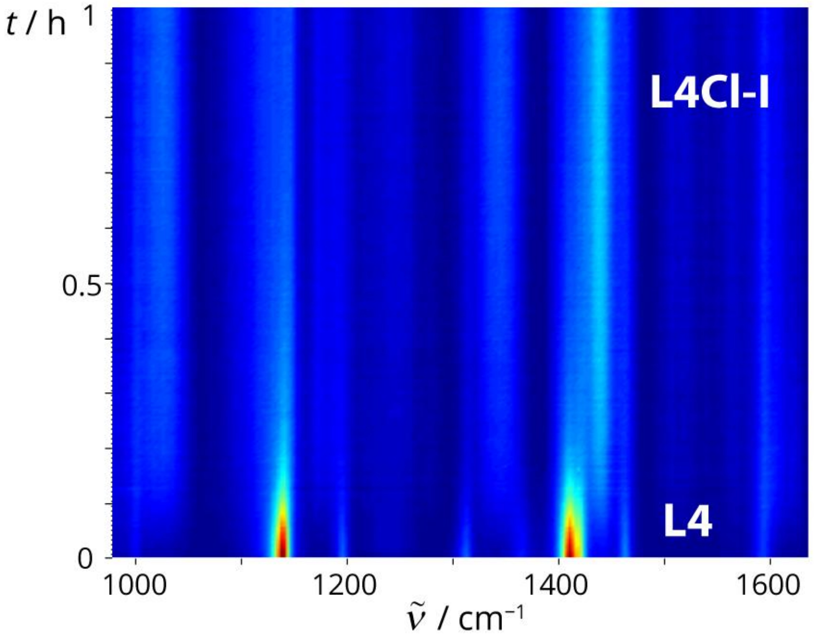

Figure S17: 2D plot of the time-resolved Raman monitoring of NG of **L4** (0.50 mmol) with NCS (0.60 mmol) and SiO_2 (250 mg).

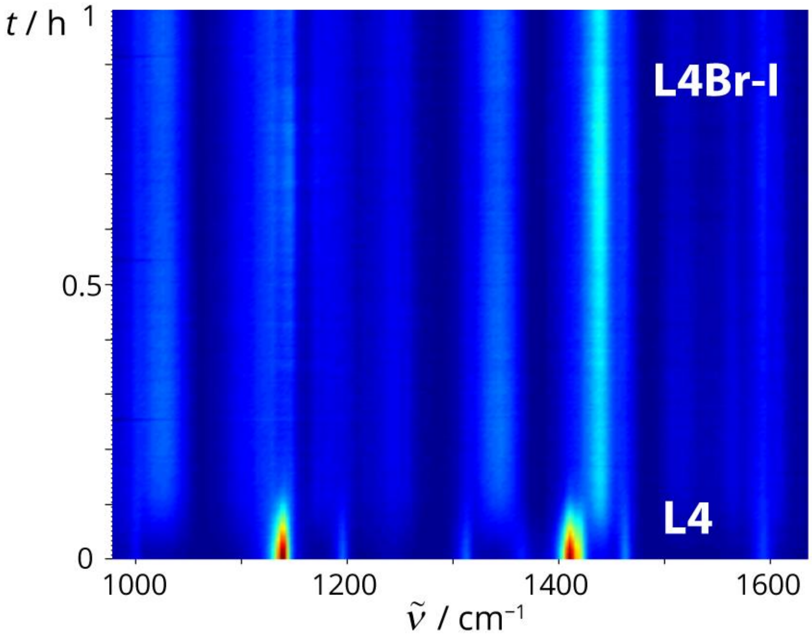

Figure S18: 2D plot of the time-resolved Raman monitoring of NG of **L4** (0.50 mmol) with NBS (0.60 mmol) and SiO_2 (250 mg).

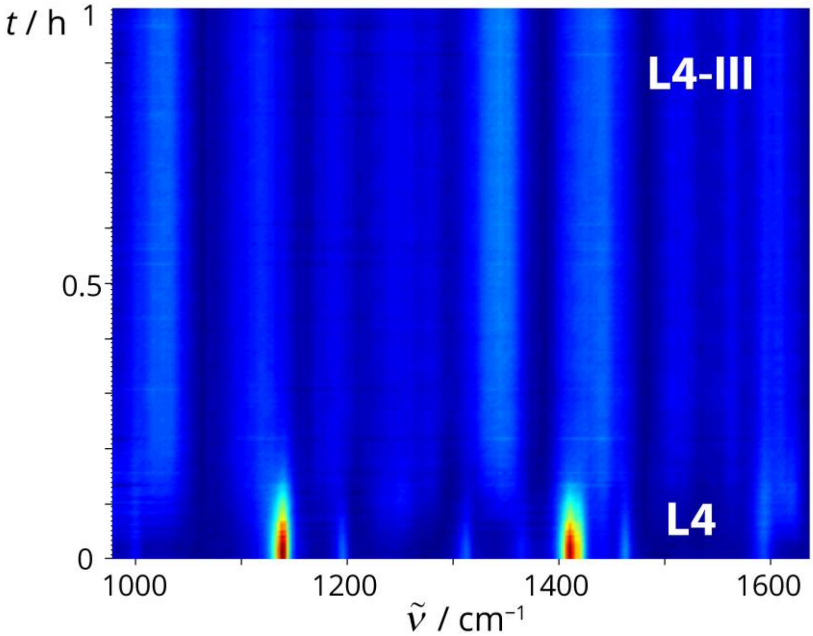

Figure S19: 2D plot of the time-resolved Raman monitoring of NG of **L4** (0.50 mmol) with NIS (0.60 mmol) and SiO_2 (250 mg).

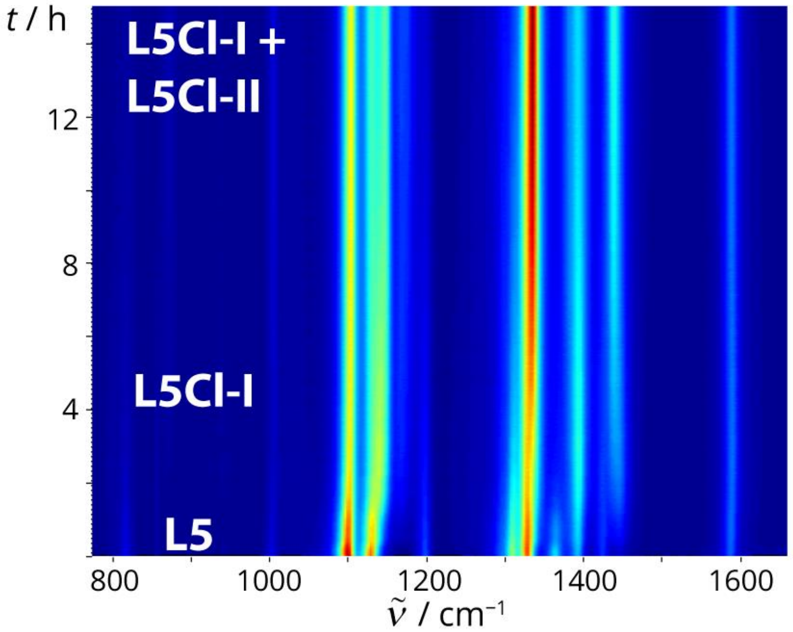

Figure S20: 2D plot of the time-resolved Raman monitoring of NG of **L5** (0.50 mmol) with NCS (0.60 mmol) and SiO_2 (250 mg).

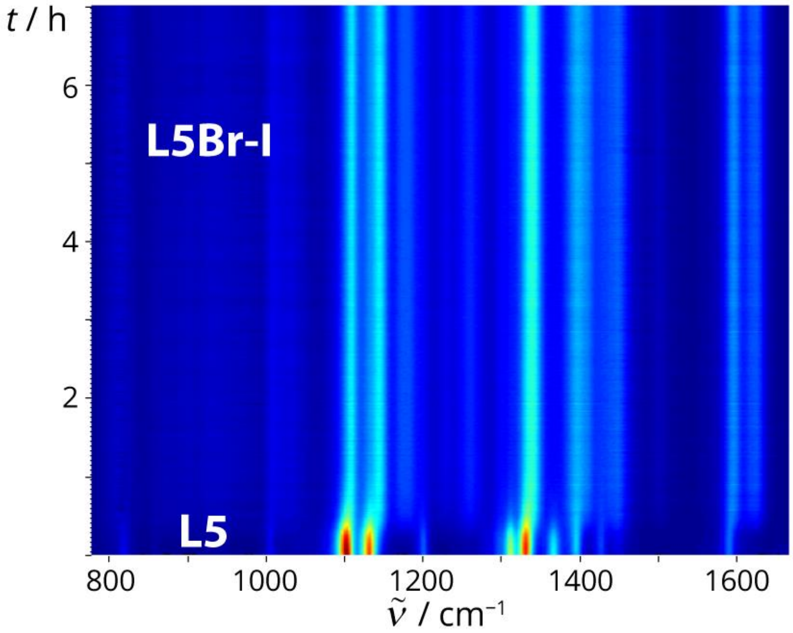

Figure S21: 2D plot of the time-resolved Raman monitoring of NG of **L5** (0.50 mmol) with NBS (0.60 mmol) and SiO_2 (250 mg).

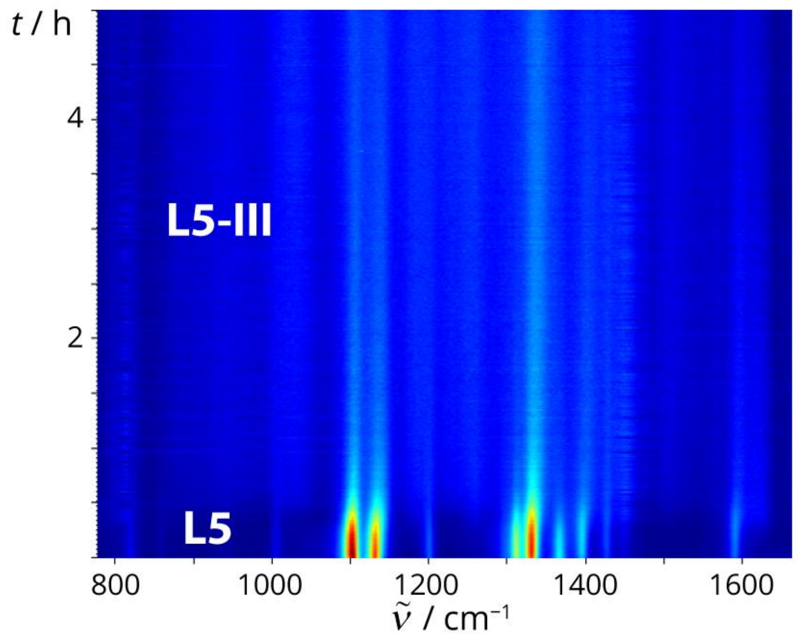

Figure S22: 2D plot of the time-resolved Raman monitoring of NG of **L5** (0.50 mmol) with NIS (0.60 mmol) and SiO_2 (250 mg).

4.2. *In situ* observation of I6-I – I8-I during mechanochemical bromination reactions

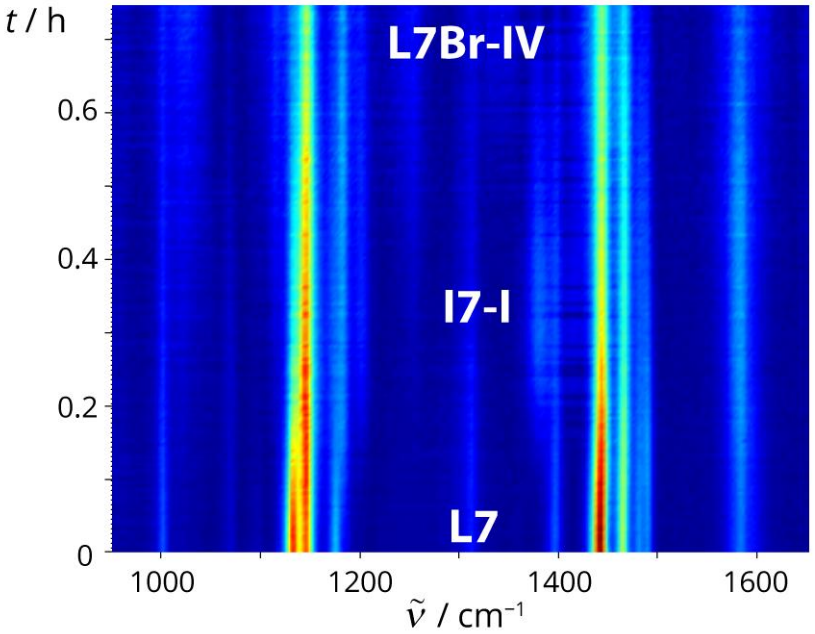

Figure S23: *In situ* observation of **I7-I** during the time-resolved Raman monitoring of LAG of **L7** (0.50 mmol) with NBS (0.6 mmol), TsOH (0.25 mmol), $Pd(OAc)_2$ (30 mol%), MeCN (15 µL) and SiO_2 (250 mg).

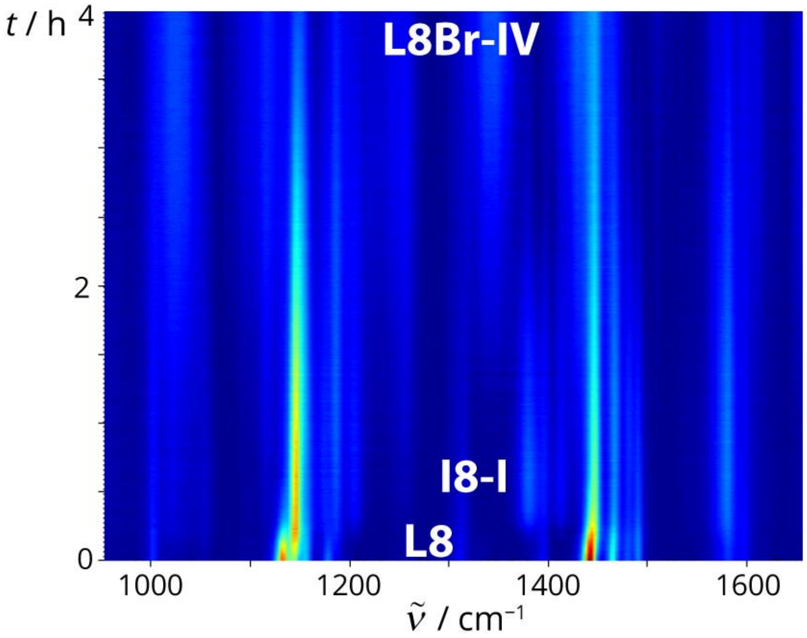

Figure S24: *In situ* observation of **I8-I** during the time-resolved Raman monitoring of LAG of **L8** (0.50 mmol) with NBS (0.6 mmol), TsOH (0.25 mmol), $Pd(OAc)_2$ (30 mol%), MeCN (15 µL) and SiO_2 (250 mg).

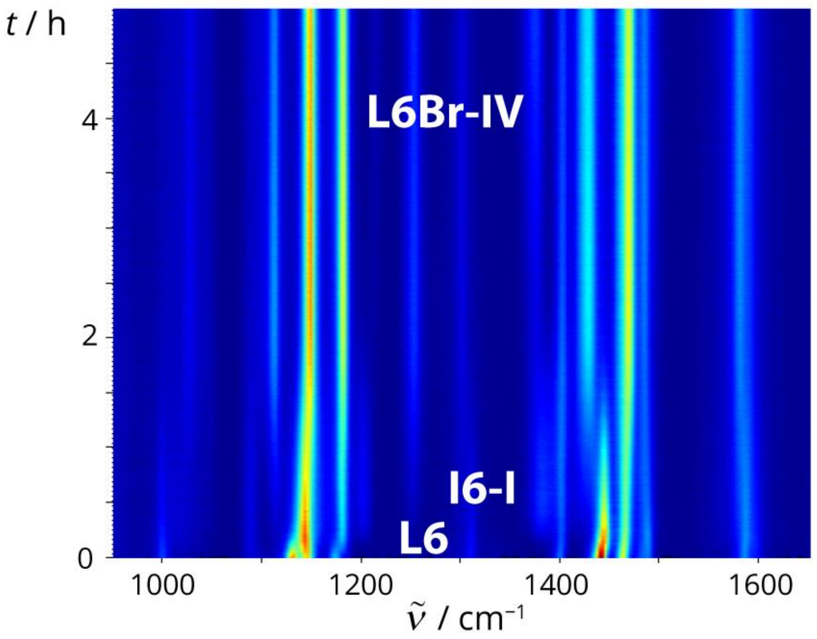

Figure S25: *In situ* observation of **I6-I** during the time-resolved Raman monitoring of LAG of **L6** (0.50 mmol) with NBS (0.6 mmol), TsOH (0.25 mmol), $Pd(OTs)_2(MeCN)_2$ (30 mol%), MeCN (15 µL) and SiO_2 (400 mg).

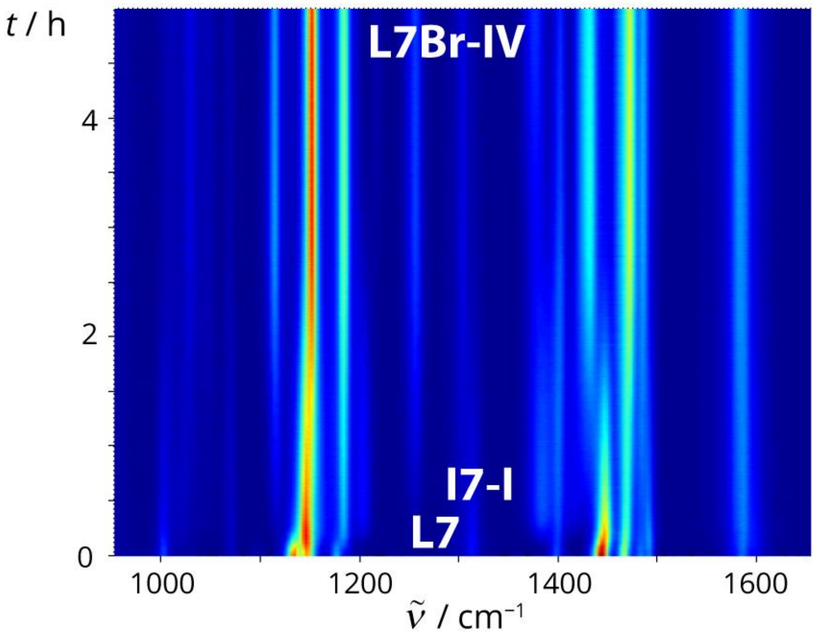

Figure S26: *In situ* observation of **I7-I** during the time-resolved Raman monitoring of LAG of **L7** (0.50 mmol) with NBS (0.6 mmol), TsOH (0.25 mmol), $Pd(OTs)_2(MeCN)_2$ (30 mol%), MeCN (15 µL) and SiO_2 (400 mg).

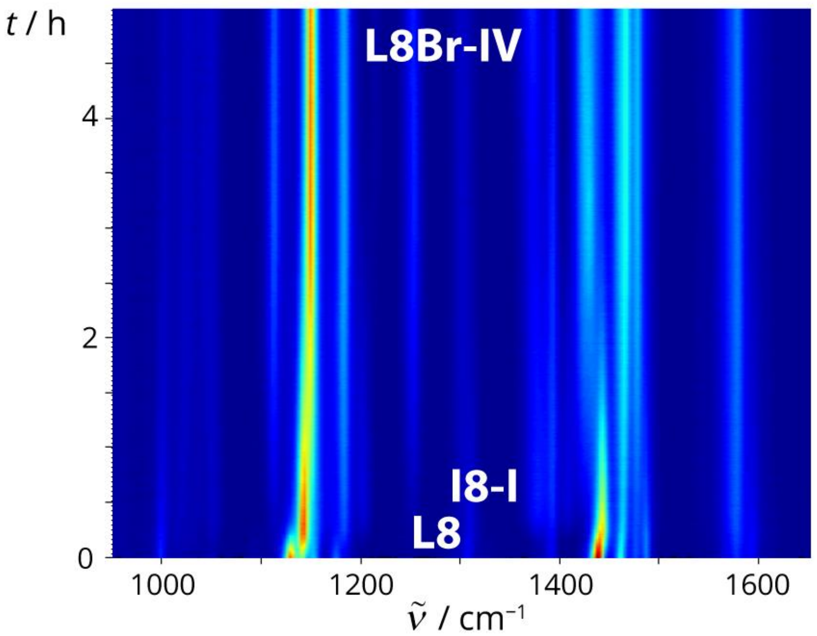

Figure S27: *In situ* observation of **I8-I** during the time-resolved Raman monitoring of LAG of **L8** (0.50 mmol) with NBS (0.6 mmol), TsOH (0.25 mmol), $Pd(OTs)_2(MeCN)_2$ (30 mol%), MeCN (15 µL) and SiO_2 (400 mg).

4.3. C–H bond activation

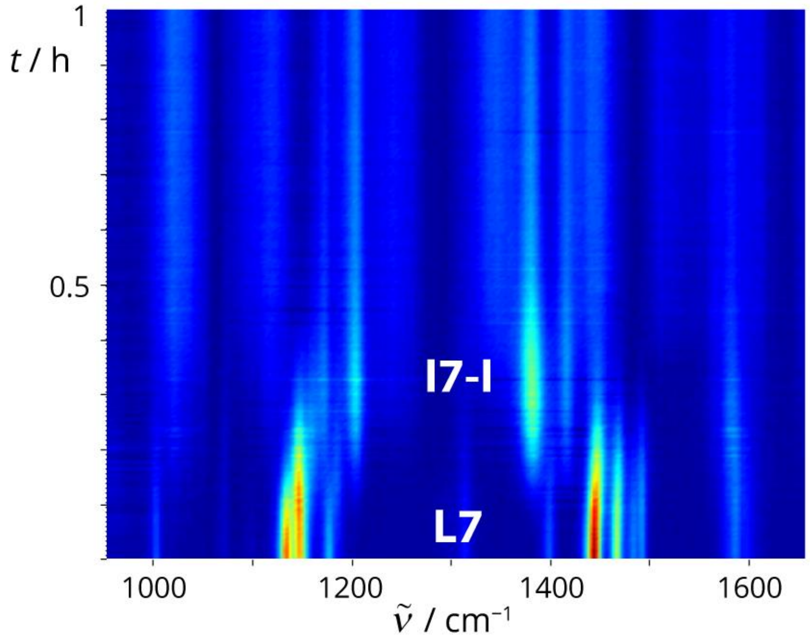

Figure S28: 2D plot of the time-resolved Raman monitoring of LAG of **L7** (0.40 mmol) with $Pd(OAc)_2$ (0.42 mmol), TsOH (0.42 mmol), MeCN (25 µL) and SiO_2 (250 mg).

4.4. Raman spectra of I6-I and I7-I

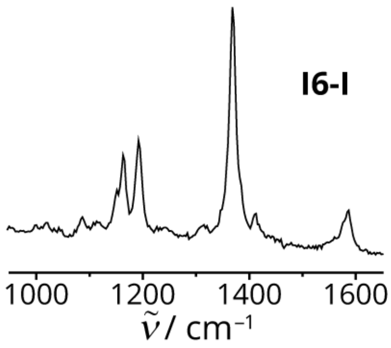

Figure S29: Solid-state Raman spectrum of **I6-I**.

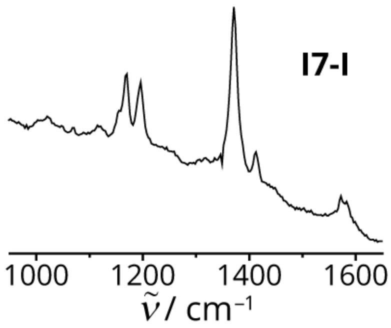

Figure S30: Solid-state Raman spectrum of **I7-I**.

5. Powder X-ray diffraction

Crystal structure of compound **I6-I**

The collected powder pattern was indexed using a monoclinic unit cell (a = 7.138 Å, b = 10.491 Å, c = 29.761 Å, a = 87.76°, b = 90.88°, g = 101.54°) with the volume of 2182 Å^3. Solved by simulated annealing in direct space with rigid-body Rietveld refinement. The P-1 space group was chosen because cell volume indicated two independent molecules in the asymmetric unit with an inversion center. Molecules in the asymmetric unit were treated independently in simulated annealing with torsions included in optimization. The starting molecular geometry was optimized in vaccuo and represented in Topas as a Z-matrix. Errors on atomic coordinates were estimated using bootstraping. Structure solution agreed with experimental PXRD data. Presented geometry had no overlap between neighboring molecules.

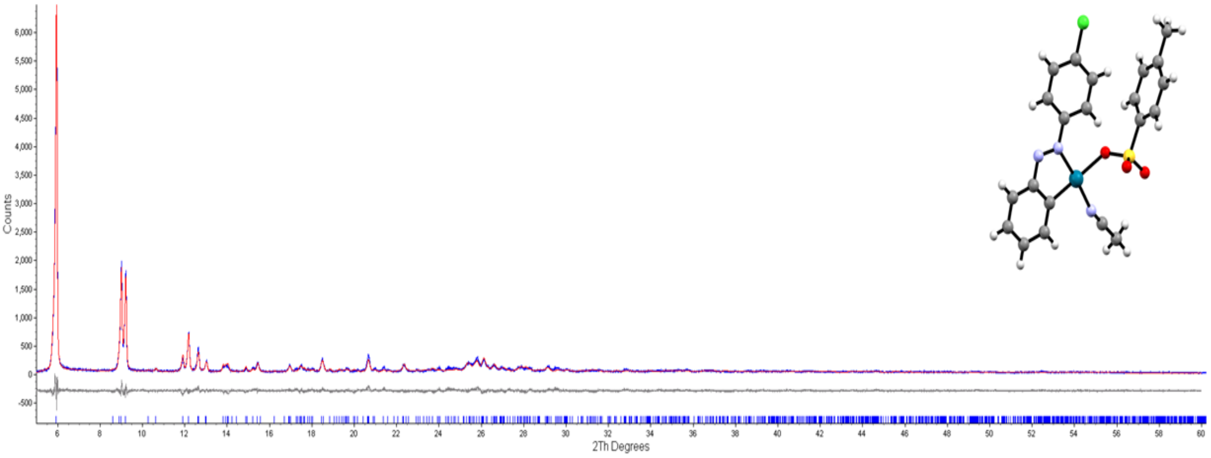

Figure S31: Rietveld plot for the crystal structure solution of compound **I6-I**.

Crystal structure of compound **I7-I**

The collected powder pattern was indexed using a monoclinic unit cell (a = 7.064 Å, b = 10.442 Å, c = 29.556 Å, α = 88.13°, β = 91.13°, γ = 101.74°) with the volume of 2133 Å^3. The crystal structure is isostructural to the Cl-substituted analogue (**I6-I**). The Z-matrix describing the molecules of **I6-I** were modified to account for longer C–Br bond distances and such a structure model was the initial point for Rietveld refinement. Errors on atomic coordinates were estimated using bootstraping. Structure solution agreed with experimental PXRD data. Presented geometry had no overlap between neighboring molecules.

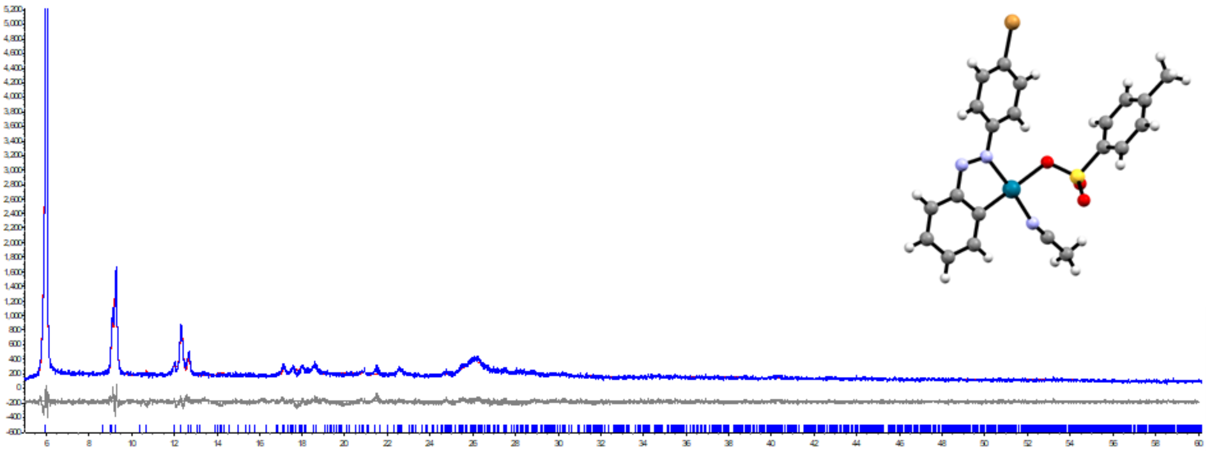

Figure S32: Rietveld plot for the crystal structure solution of compound **I7-I**.

6. Single-crystal X-ray diffraction

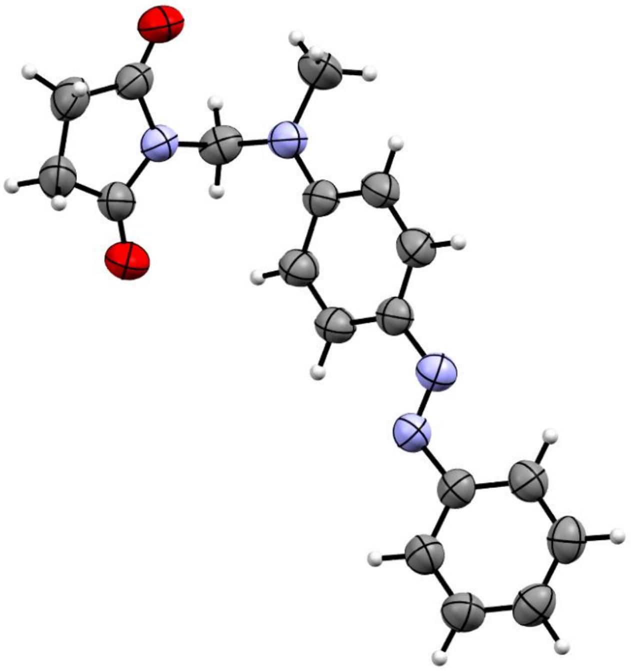

Figure S33: ORTEP plot of **L4-III**, represented by thermal ellipsoids shown at the 50% probability level.

Table S1: Crystal data and structure refinements parameters for **L4-III**.

Formula	$C_{18}H_{18}N_4O_2$
Crystal system	Monoclinic
Crystal size / mm^3	0.2×0.1×0.1
Crystal habitus	Needle
Crystal colour	Brown
Space group	P 2_1/n
Temperature / K	293
Diffractometer (Oxford diffraction)	XtaLAB Synergy, Dualflex, HyPix
Radiation, λ / Å	1.54184
Unit cell dimensions	
a / Å	6.79478(9)
b / Å	18.3256(2)
c / Å	12.96063(16)
α / °	90
β / °	94.7795(12)
γ / °	90
V / Å^3	1608.23(4)
Z	4
r_{calc} / g cm^{-3}	1.331
μ / mm^{-1}	0.79
F(000)	680
Reflections (collected /independent)	12828/3424
No. observed refl. [I>2σ(I)]	3424
No. restraints /No. parameters	0/263
R/wR [I>2σ(I)]	0.0357/0.1071
R/wR [all data]	0.0398/0.1107
Goodness-of-fit	1.110
Largest diff. peak and hole / e Å^{-3}	0.14 / −0.15

7. NMR data

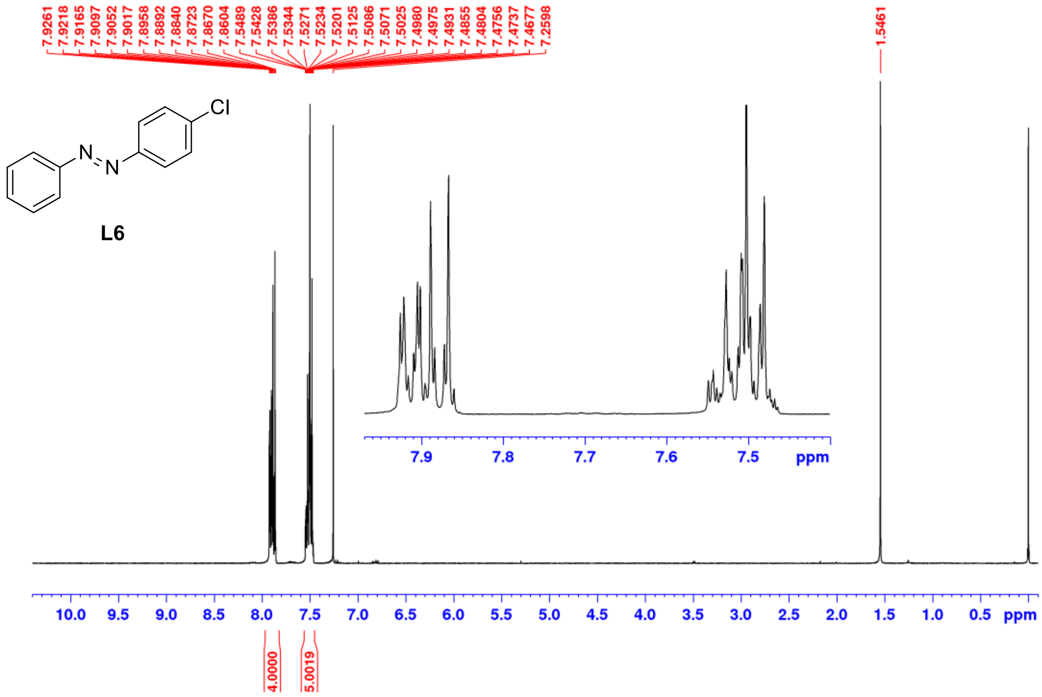

Figure S34: ^{1}H NMR spectrum of **L6** in $CDCl_3$ (400 MHz).

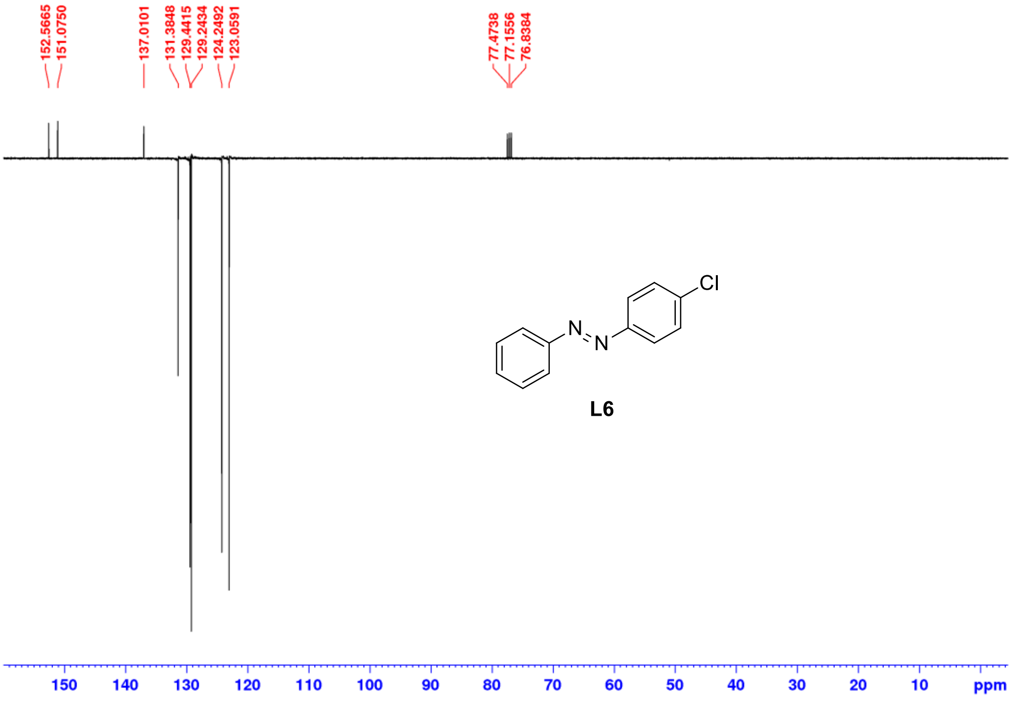

Figure S35: DEPTQ NMR spectrum of **L6** in $CDCl_3$ (101 MHz).

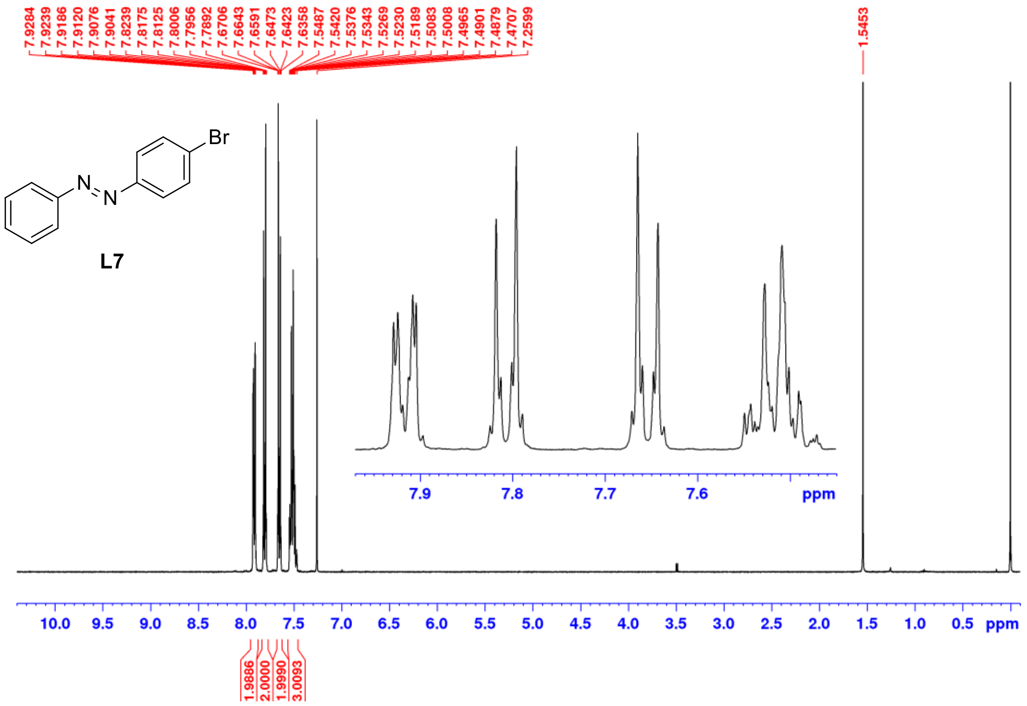

Figure S36: ^{1}H NMR spectrum of **L7** in $CDCl_3$ (400 MHz).

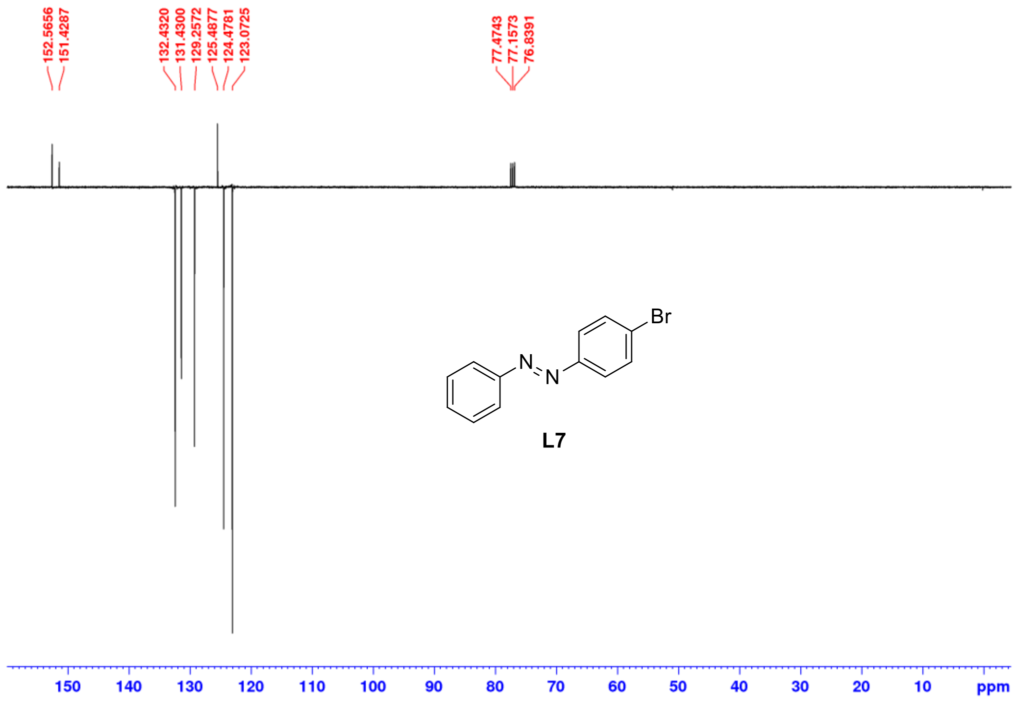

Figure S37: DEPTQ NMR spectrum of **L7** in $CDCl_3$ (101 MHz).

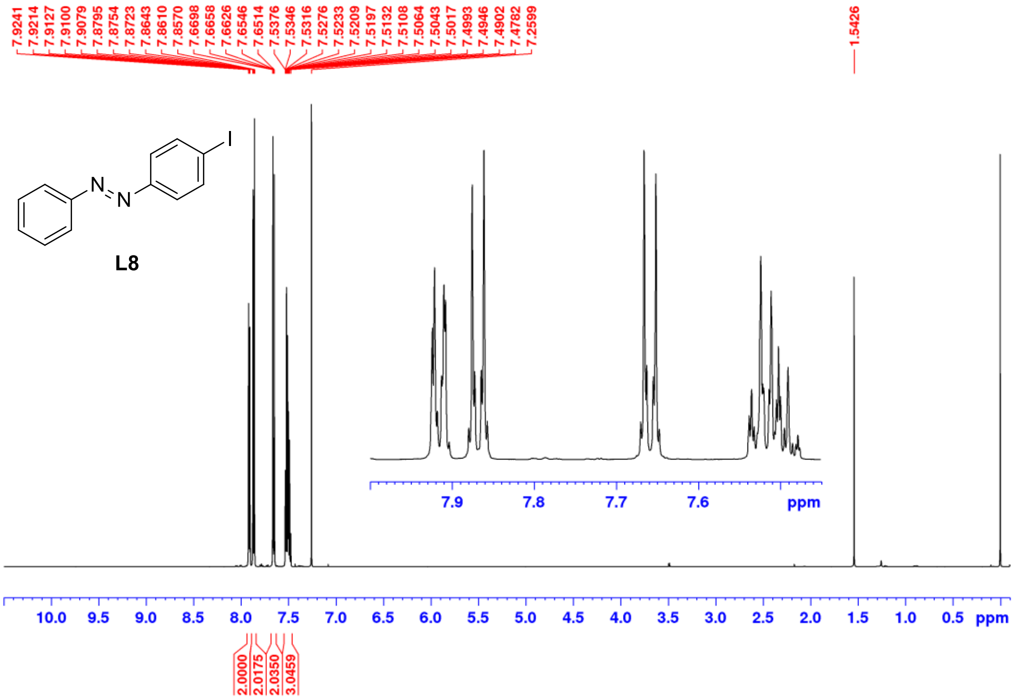

Figure S38: ^{1}H NMR spectrum of **L8** in $CDCl_3$ (400 MHz).

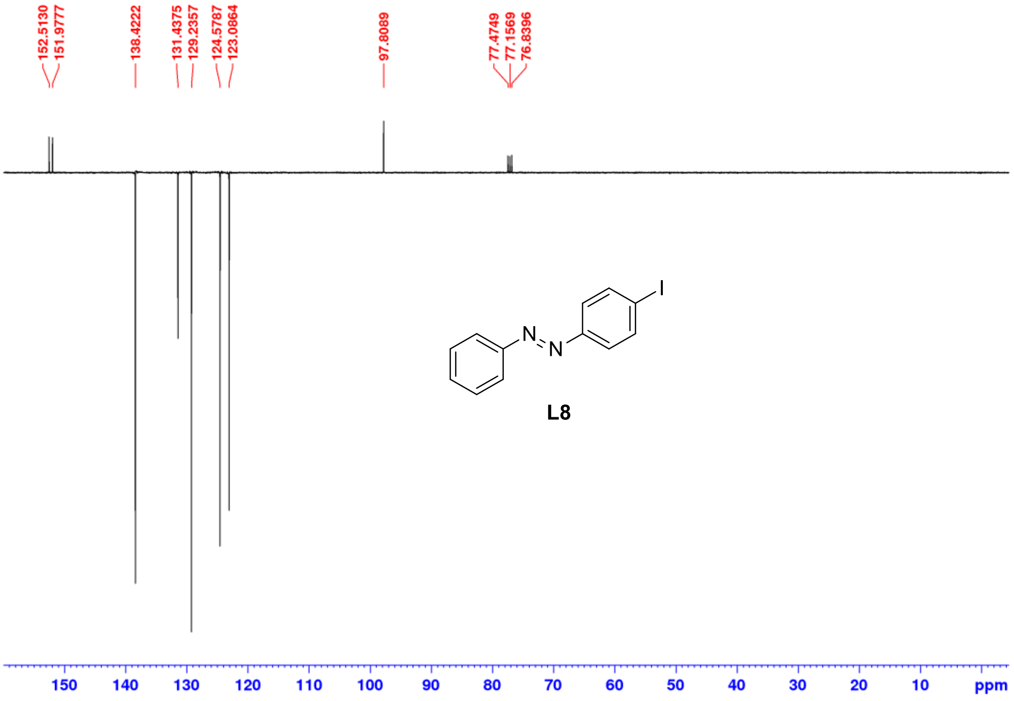

Figure S39: DEPTQ NMR spectrum of **L8** in $CDCl_3$ (101 MHz).

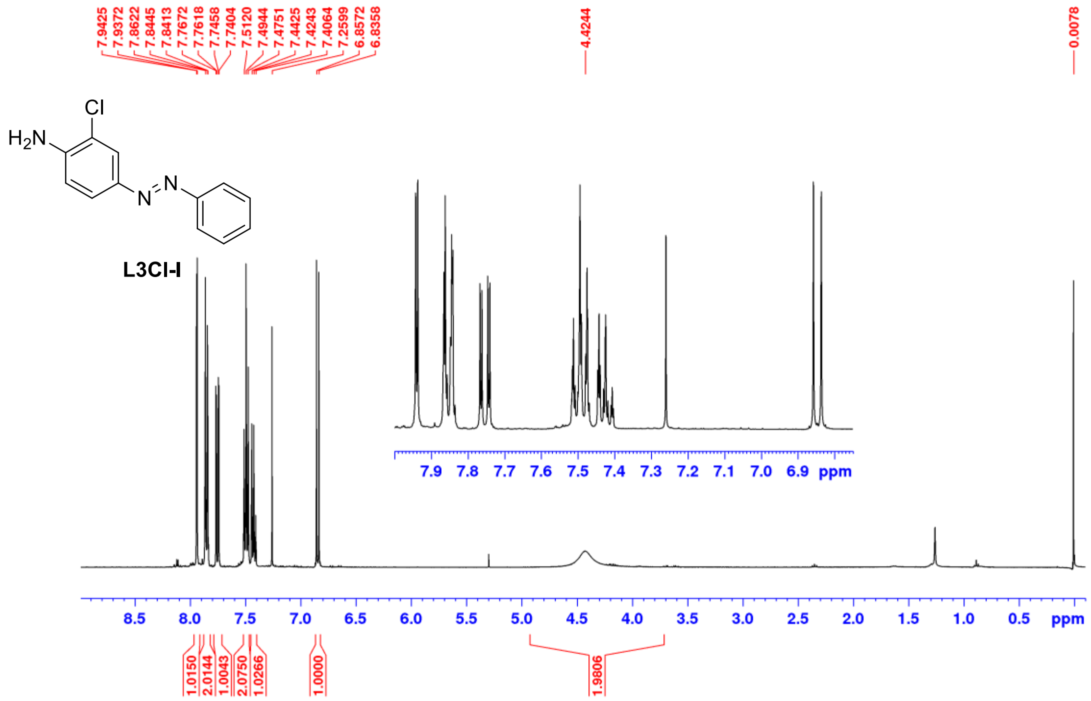

Figure S40: ^{1}H NMR spectrum of **L3Cl-I** in $CDCl_3$ (400 MHz).

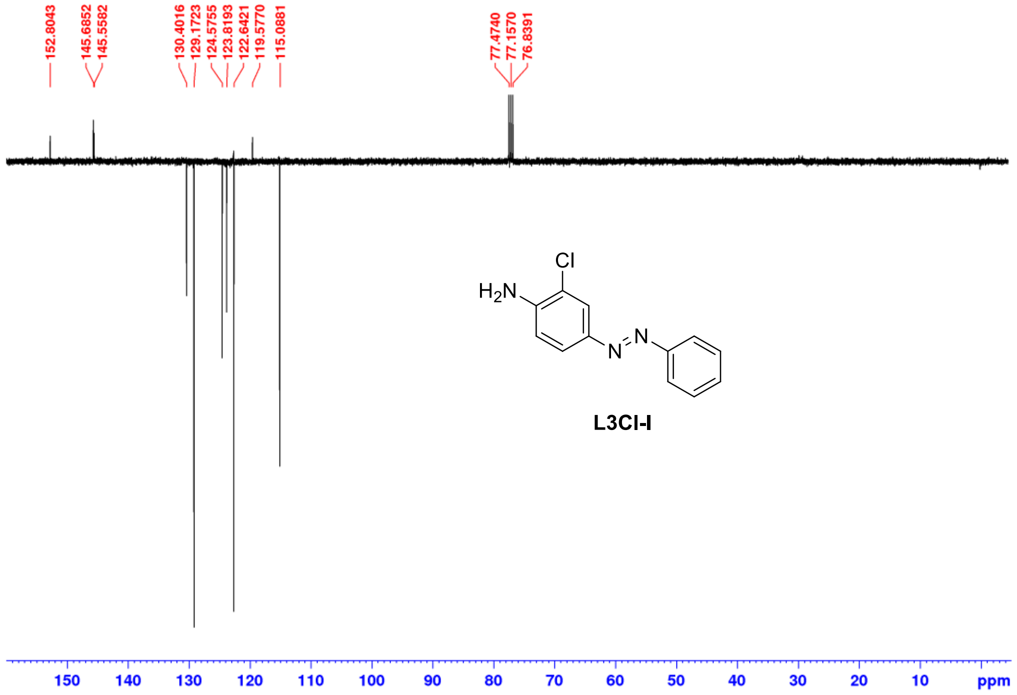

Figure S41: DEPTQ NMR spectrum of **L3Cl-I** in $CDCl_3$ (101 MHz).

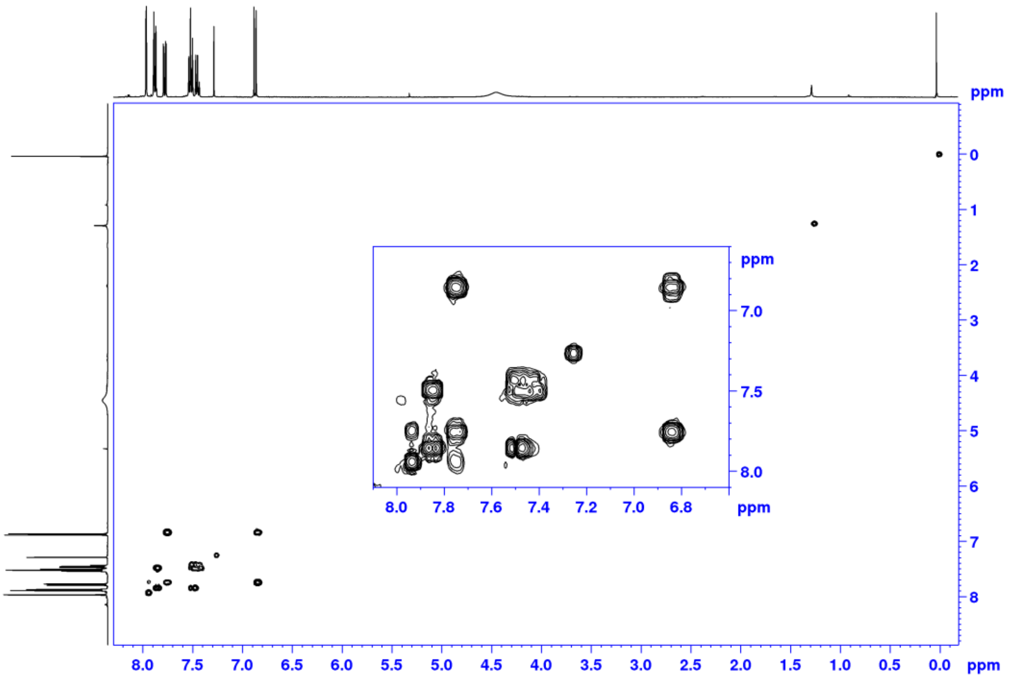

Figure S42: COSY spectrum of **L3Cl-I** in $CDCl_3$ (400 MHz).

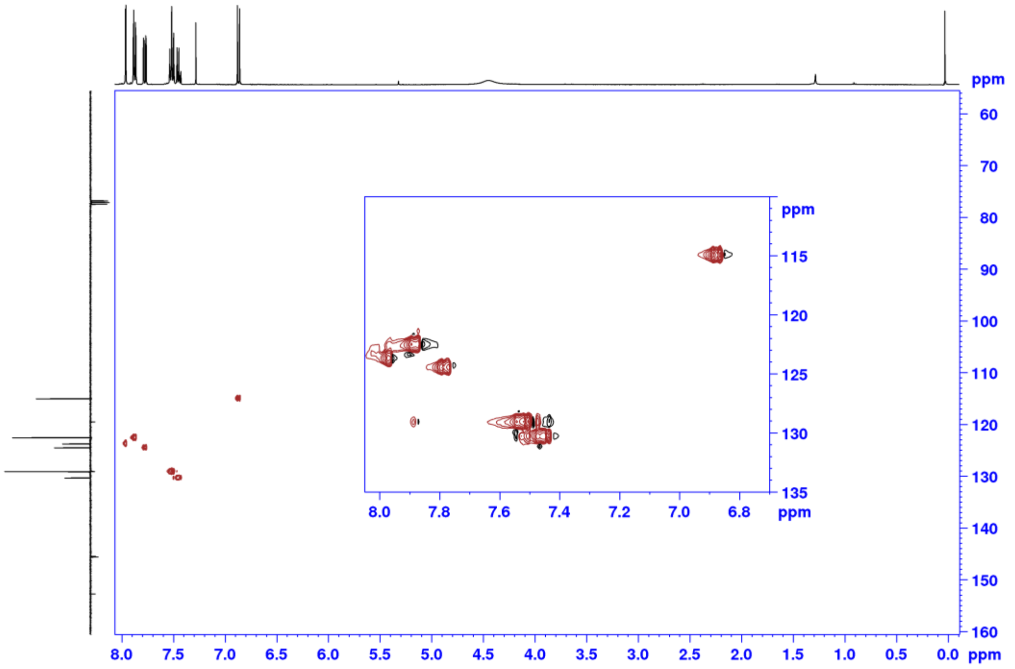

Figure S43: HSQC spectrum of **L3Cl-I** in $CDCl_3$ (400 MHz).

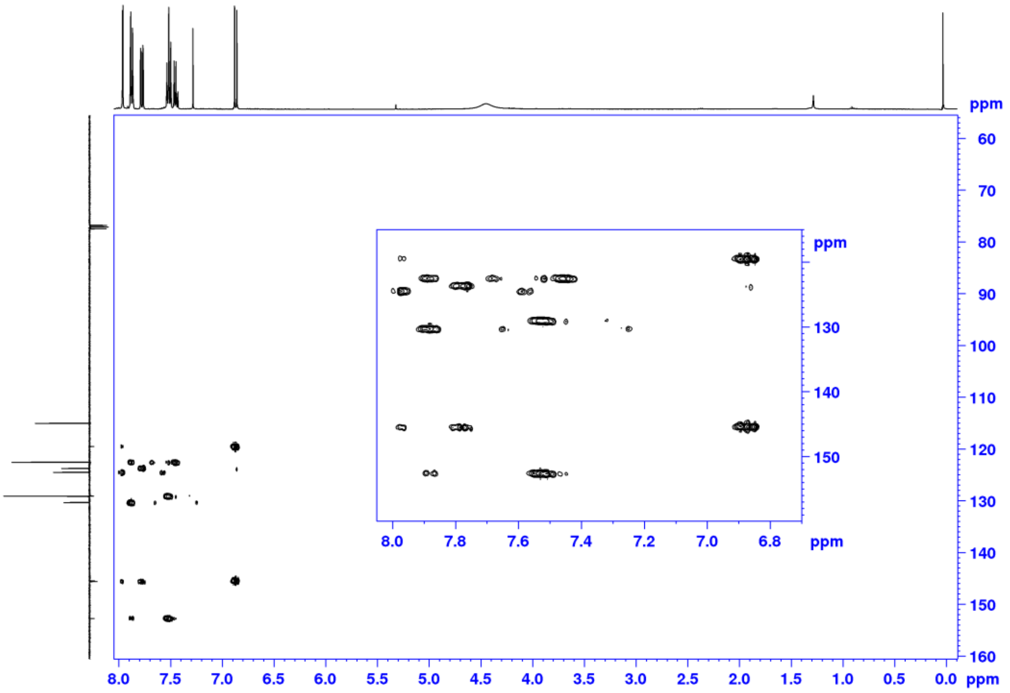

Figure S44: HMBC spectrum of **L3Cl-I** in $CDCl_3$ (400 MHz).

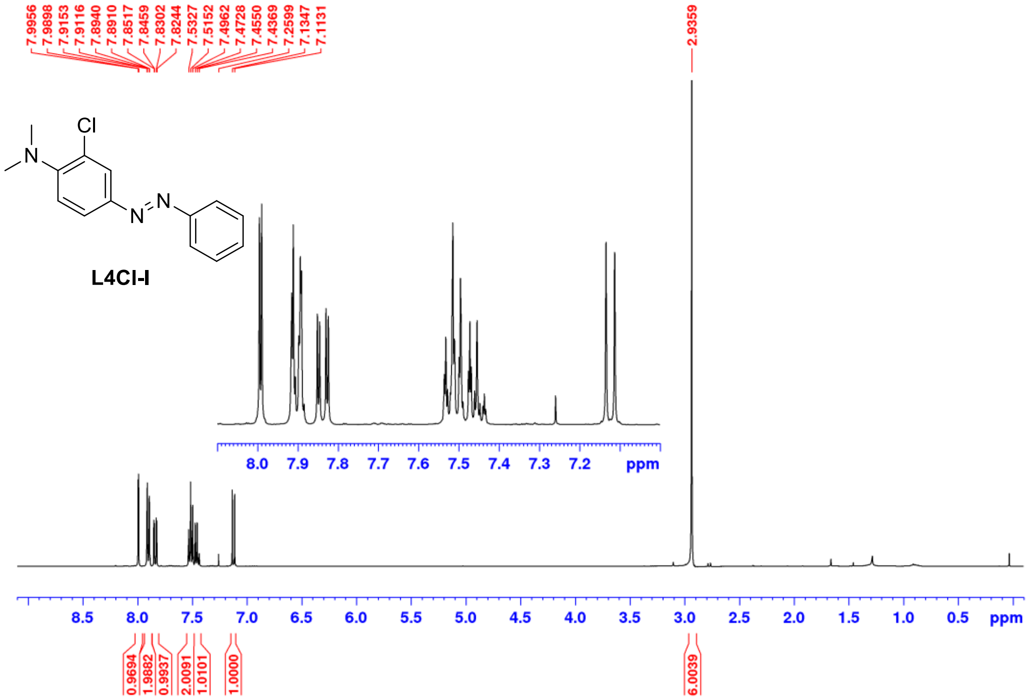

Figure S45: ^{1}H NMR spectrum of **L4Cl-I** in $CDCl_3$ (400 MHz).

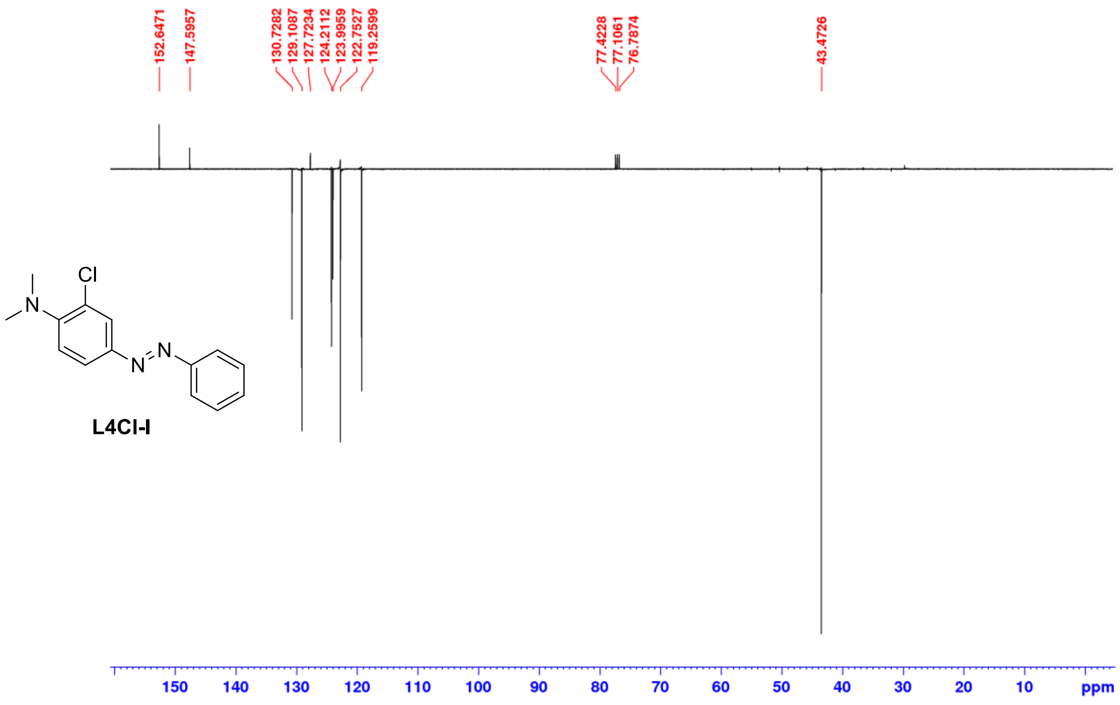

Figure S46: DEPTQ NMR spectrum of **L4Cl-I** in $CDCl_3$ (101 MHz).

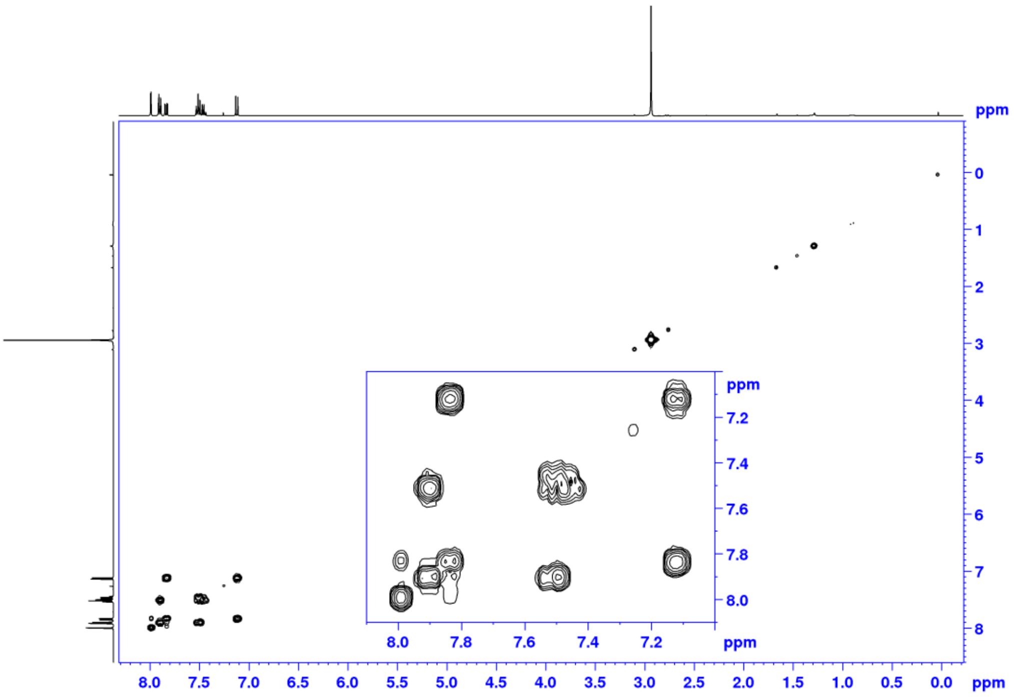

Figure S47: COSY spectrum of **L4Cl-I** in $CDCl_3$ (400 MHz).

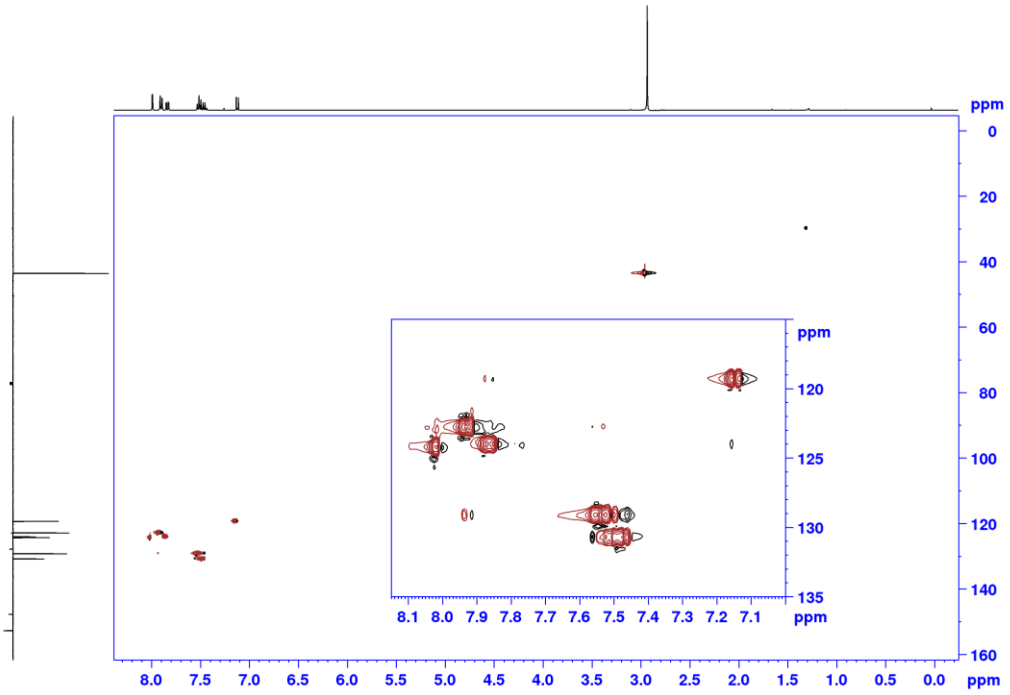

Figure S48: HSQC spectrum of **L4Cl-I** in $CDCl_3$ (400 MHz).

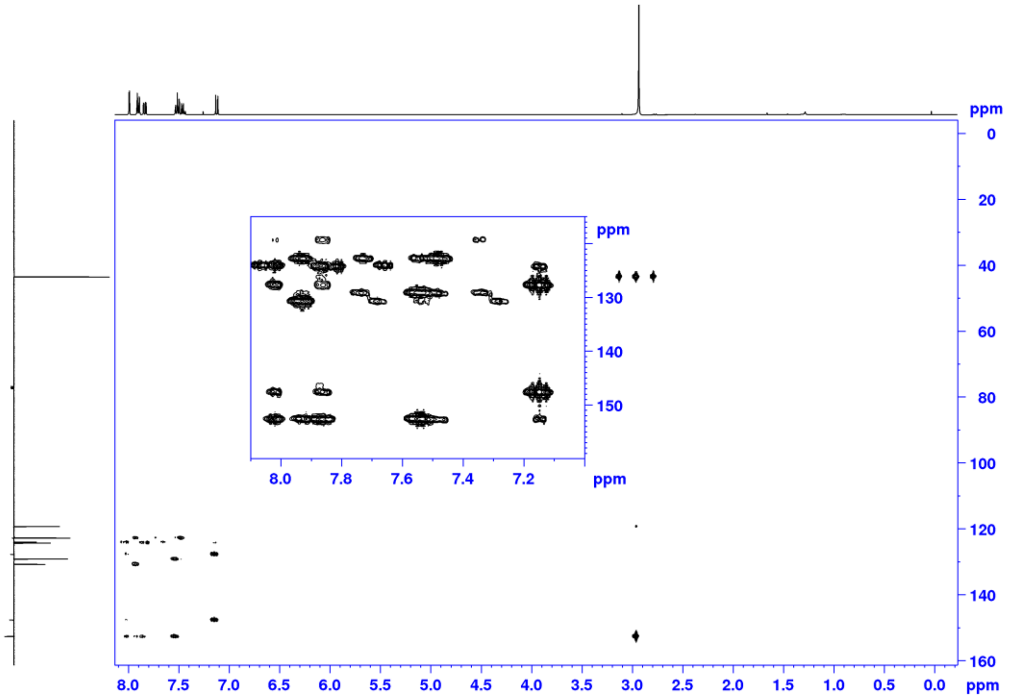

Figure S49: HMBC spectrum of **L4Cl-I** in $CDCl_3$ (400 MHz).

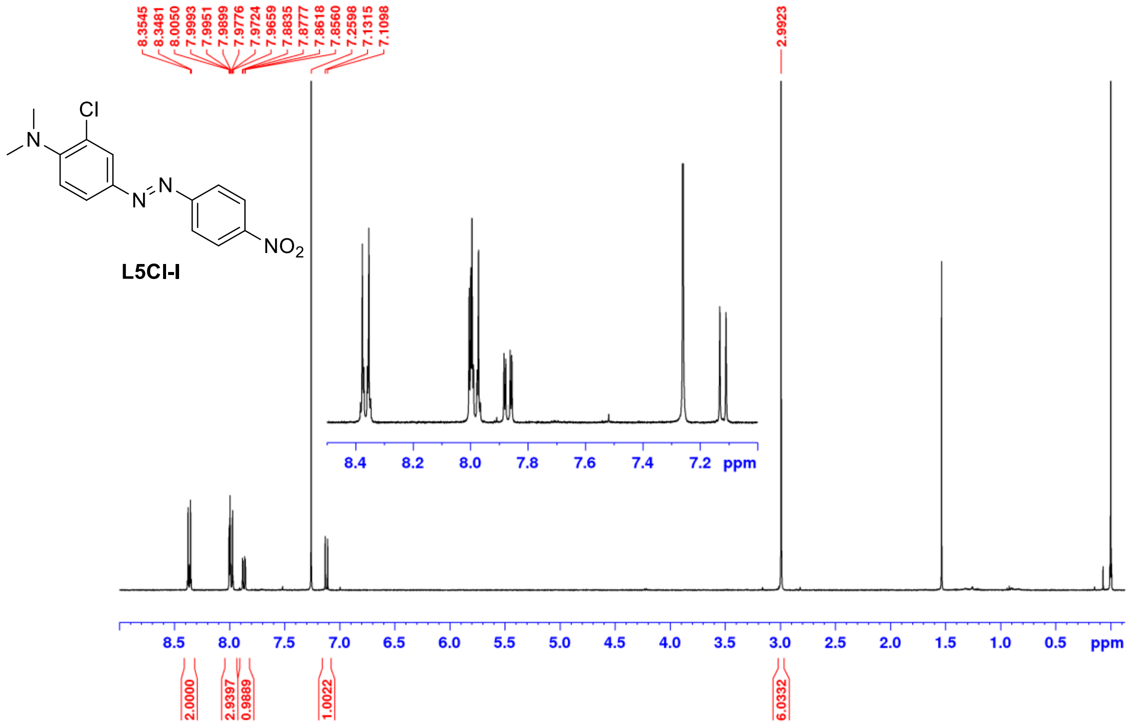

Figure S50: ^{1}H NMR spectrum of **L5Cl-I** in $CDCl_3$ (400 MHz).

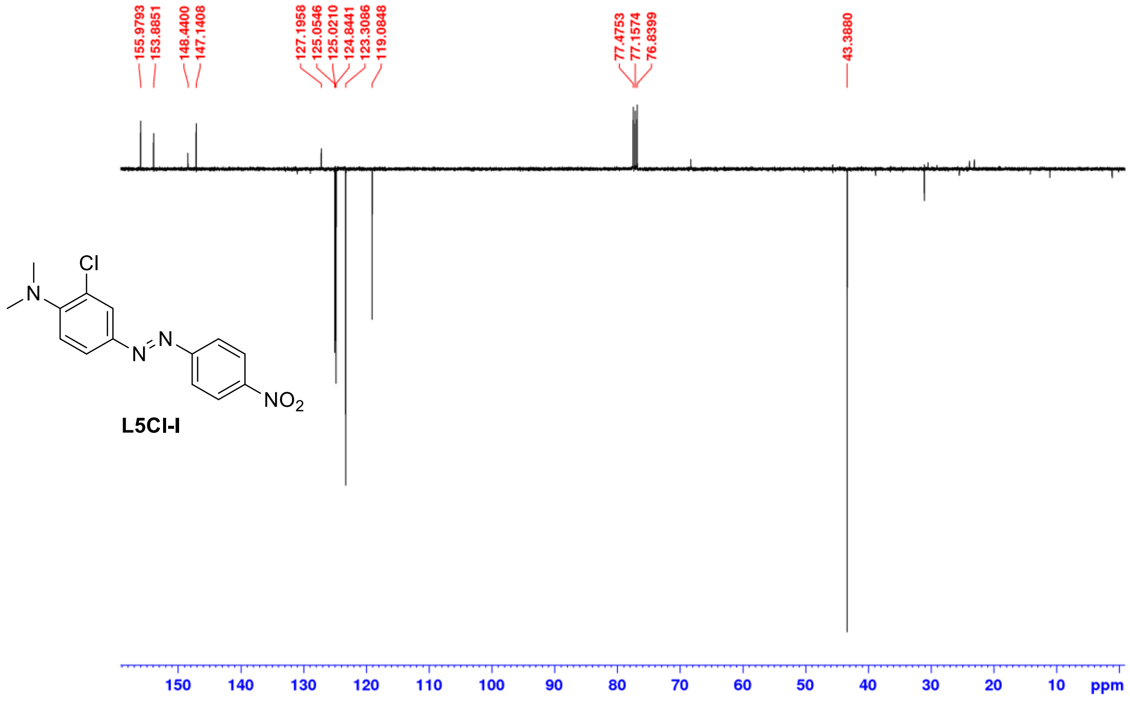

Figure S51: DEPTQ NMR spectrum of **L5Cl-I** in $CDCl_3$ (101 MHz).

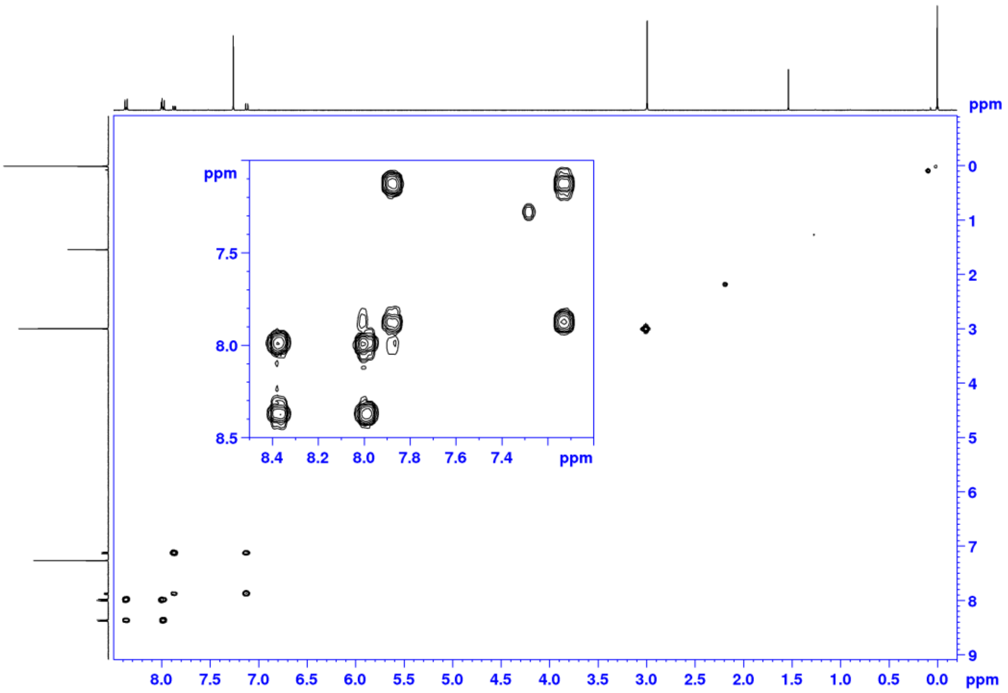

Figure S52: COSY spectrum of **L5Cl-I** in $CDCl_3$ (400 MHz).

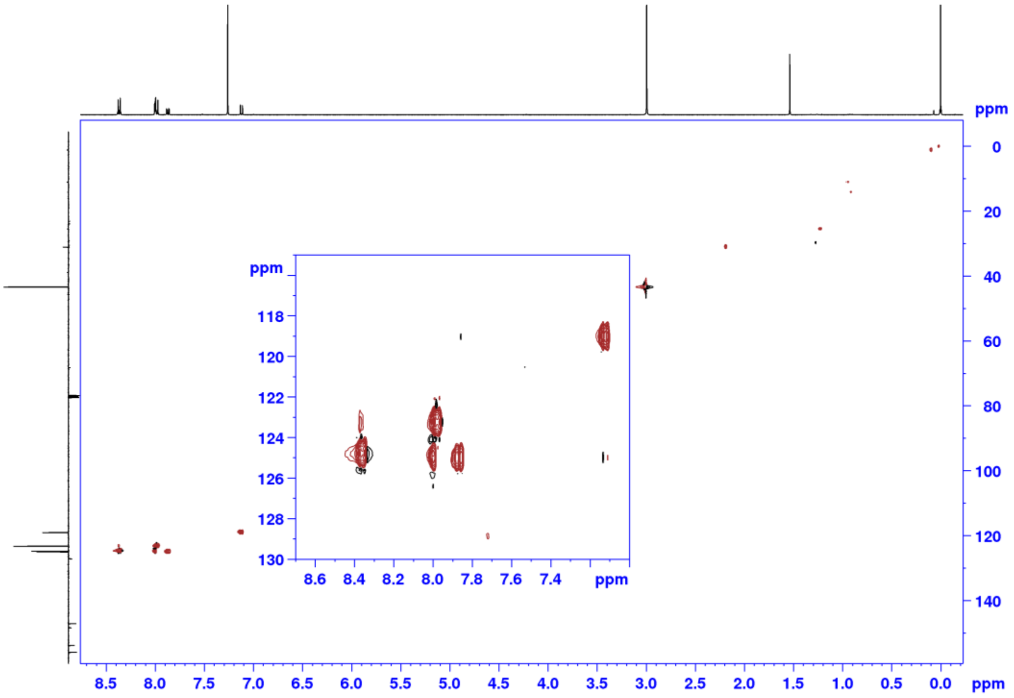

Figure S53: HSQC spectrum of **L5Cl-I** in $CDCl_3$ (400 MHz).

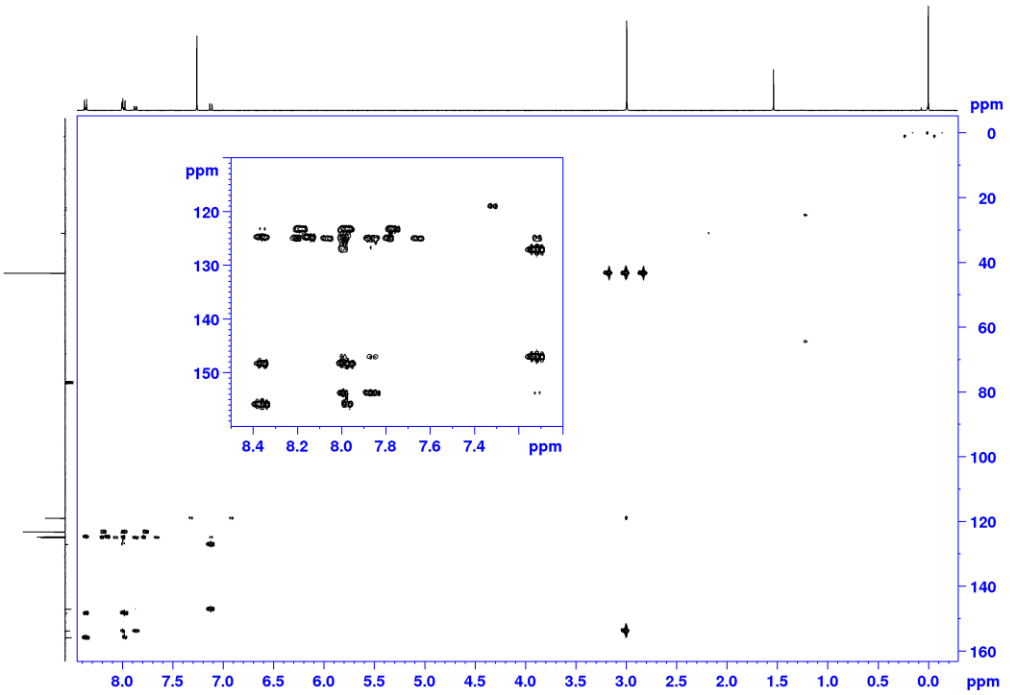

Figure S54: HMBC spectrum of **L5Cl-I** in $CDCl_3$ (400 MHz).

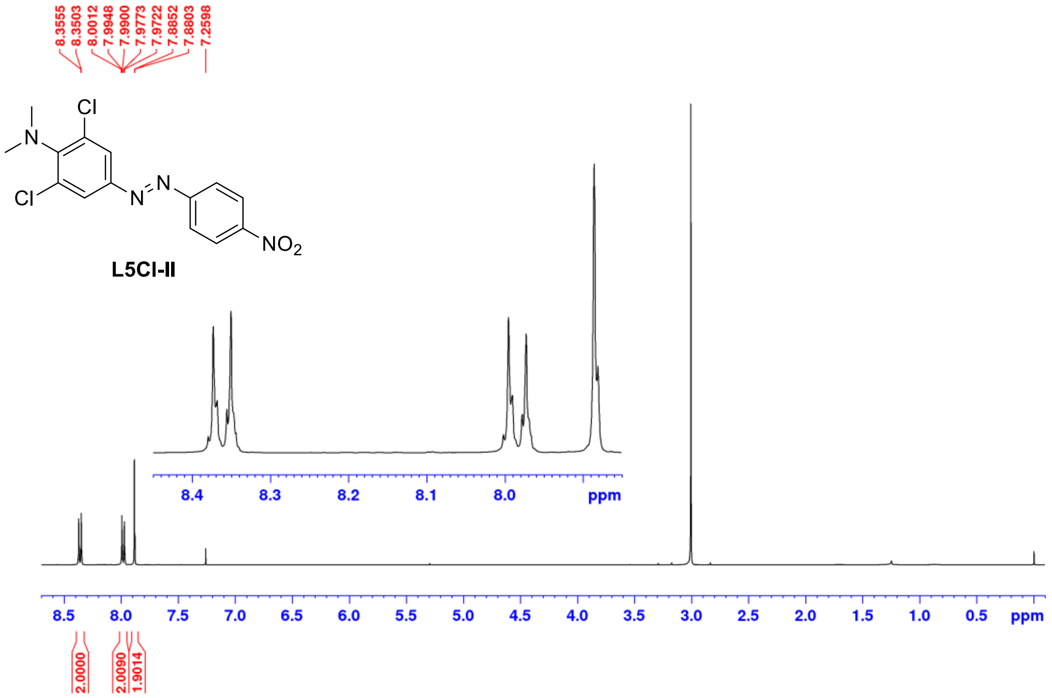

Figure S55: ^{1}H NMR spectrum of **L5Cl-II** in $CDCl_3$ (400 MHz).

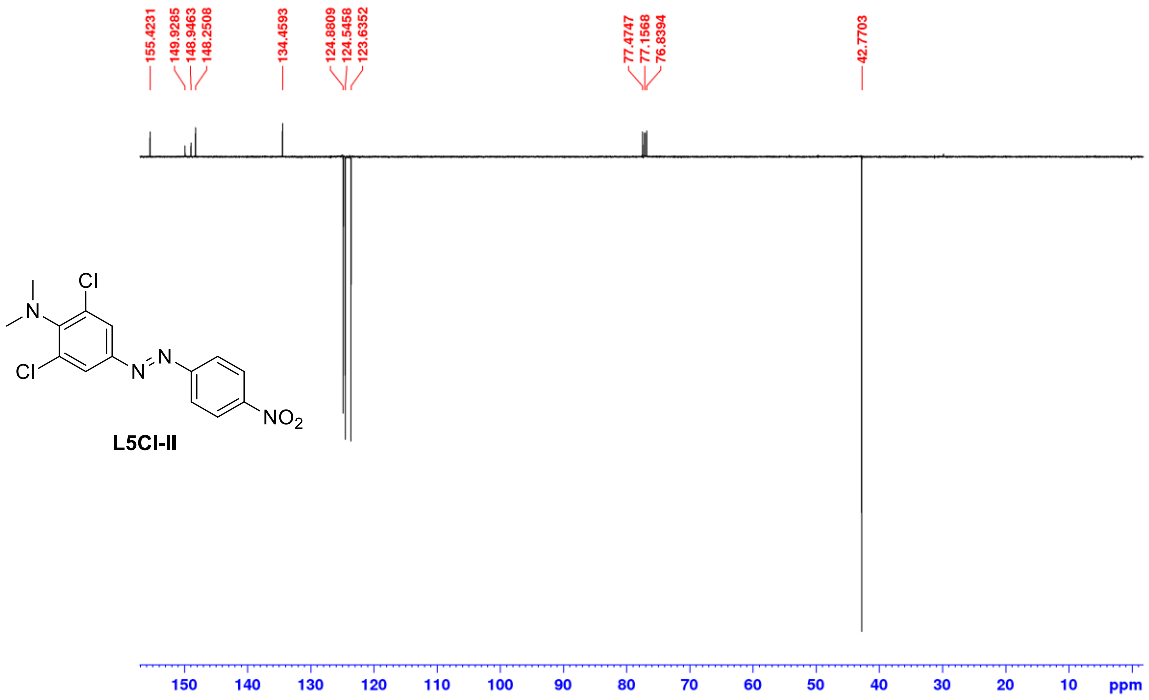

Figure S56: DEPTQ NMR spectrum of **L5Cl-II** in $CDCl_3$ (101 MHz).

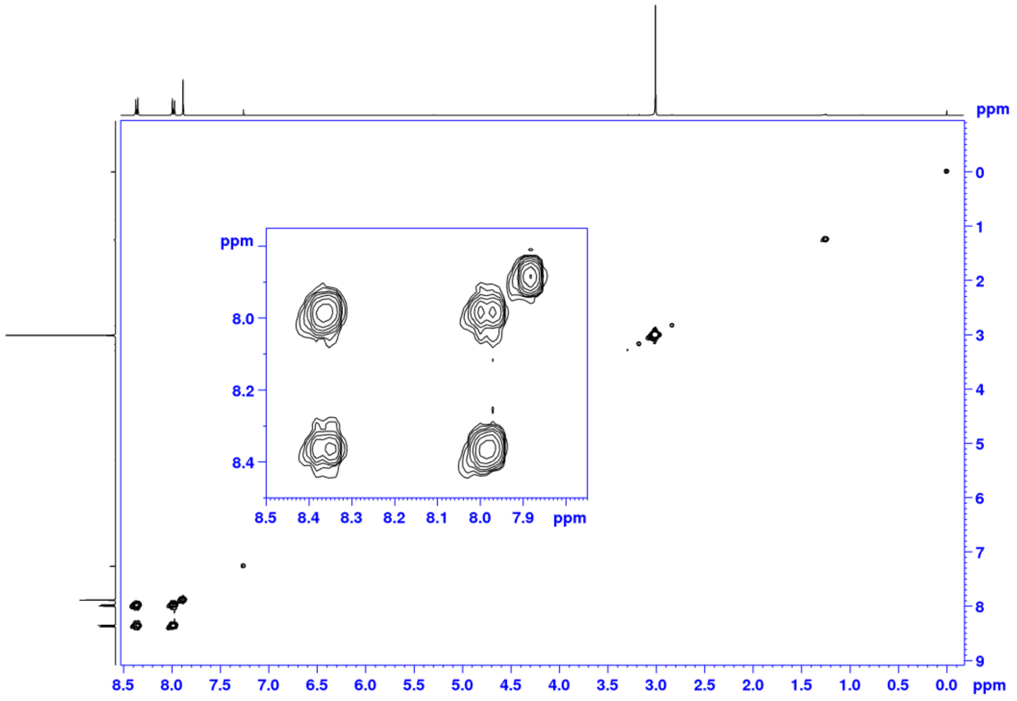

Figure S57: COSY spectrum of **L5Cl-II** in $CDCl_3$ (400 MHz).

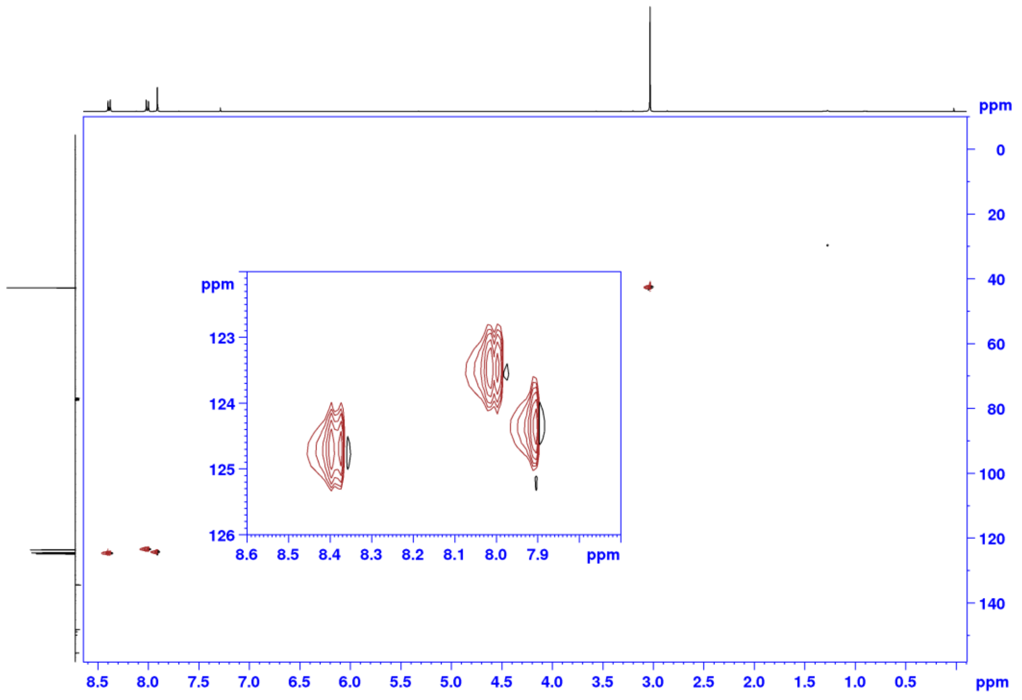

Figure S58: HSQC spectrum of **L5Cl-II** in $CDCl_3$ (400 MHz).

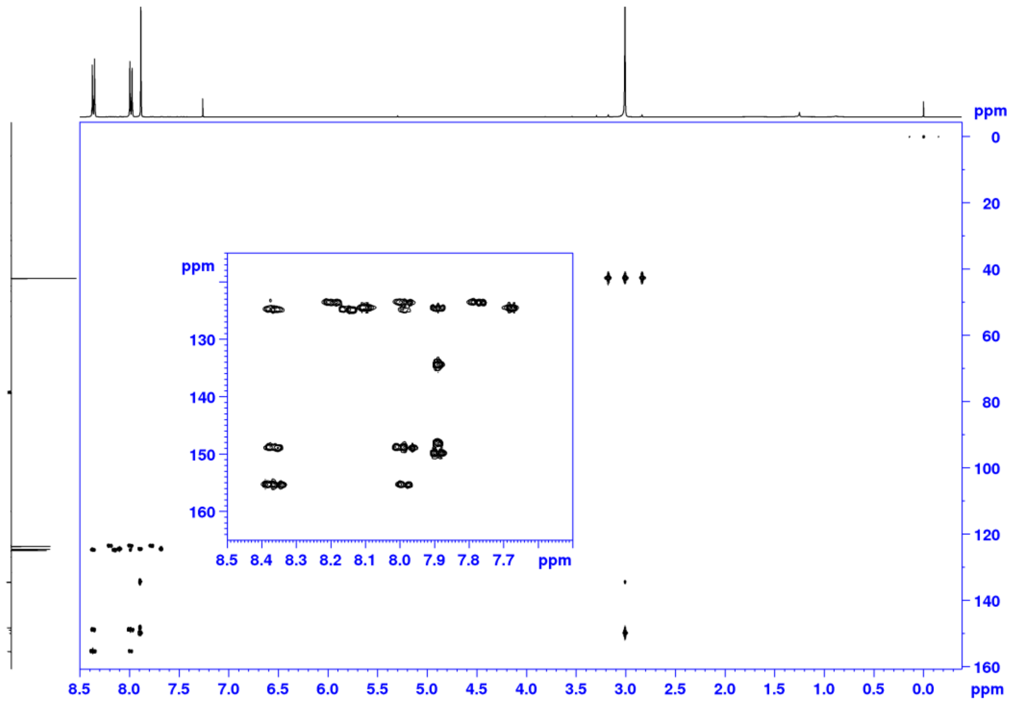

Figure S59: HMBC spectrum of **L5Cl-II** in $CDCl_3$ (400 MHz).

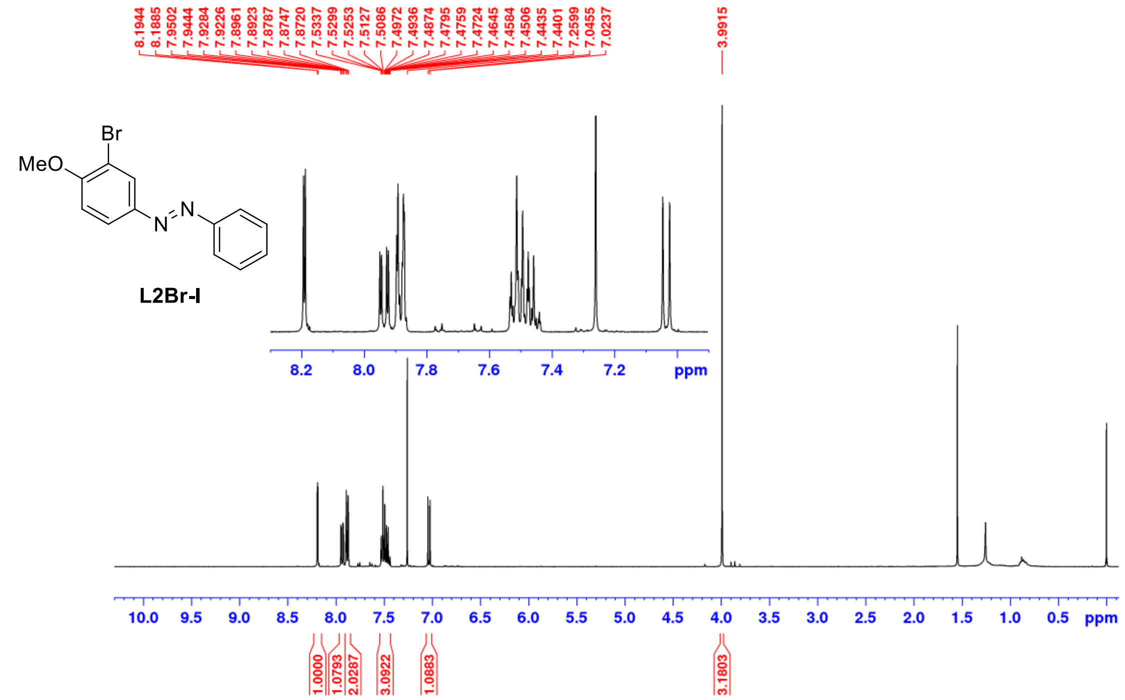

Figure S60: ^{1}H NMR spectrum of **L2Br-I** in $CDCl_3$ (400 MHz).

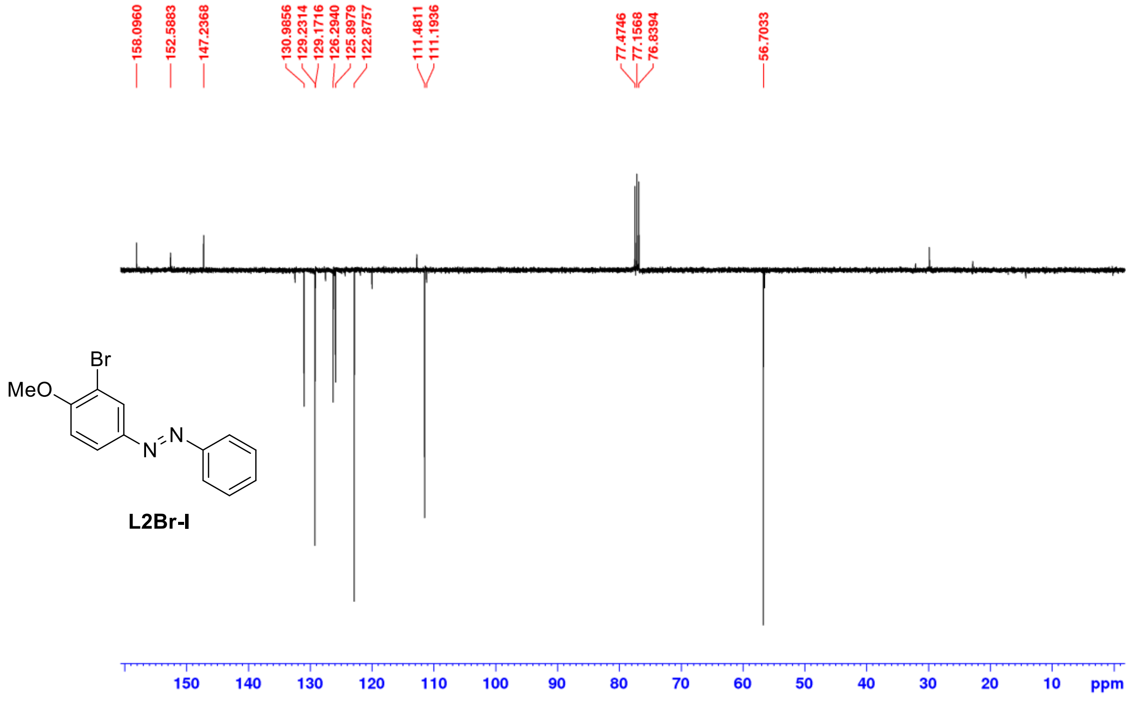

Figure S61: DEPTQ NMR spectrum of **L2Br-I** in $CDCl_3$ (101 MHz).

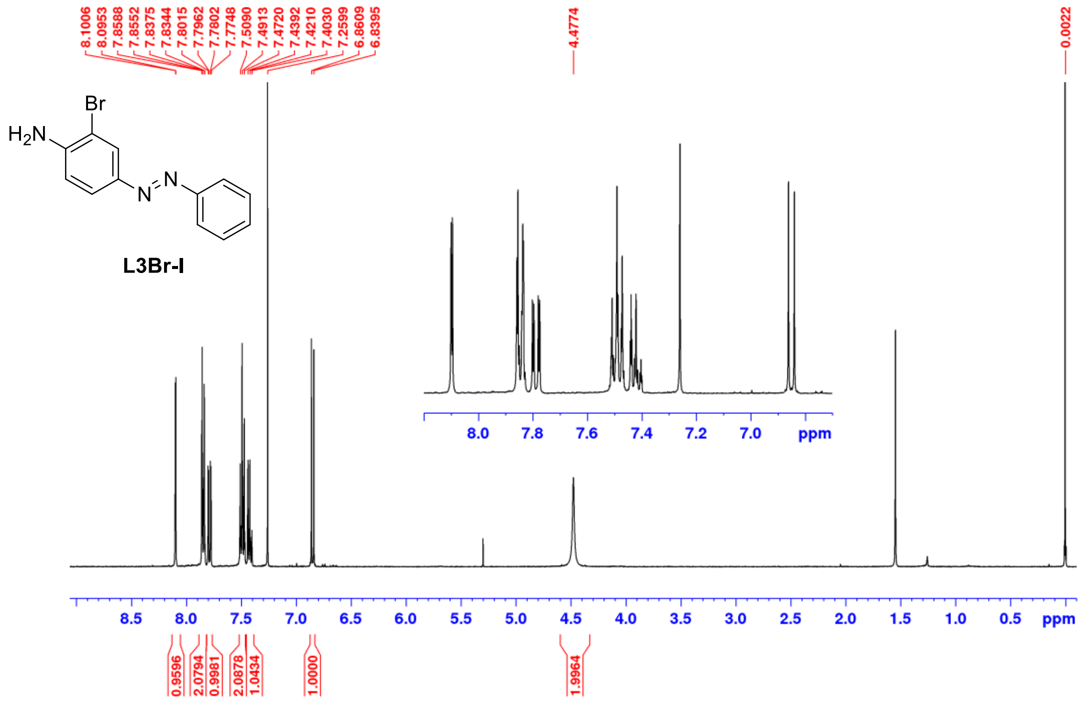

Figure S62: ^{1}H NMR spectrum of **L3Br-I** in $CDCl_3$ (400 MHz).

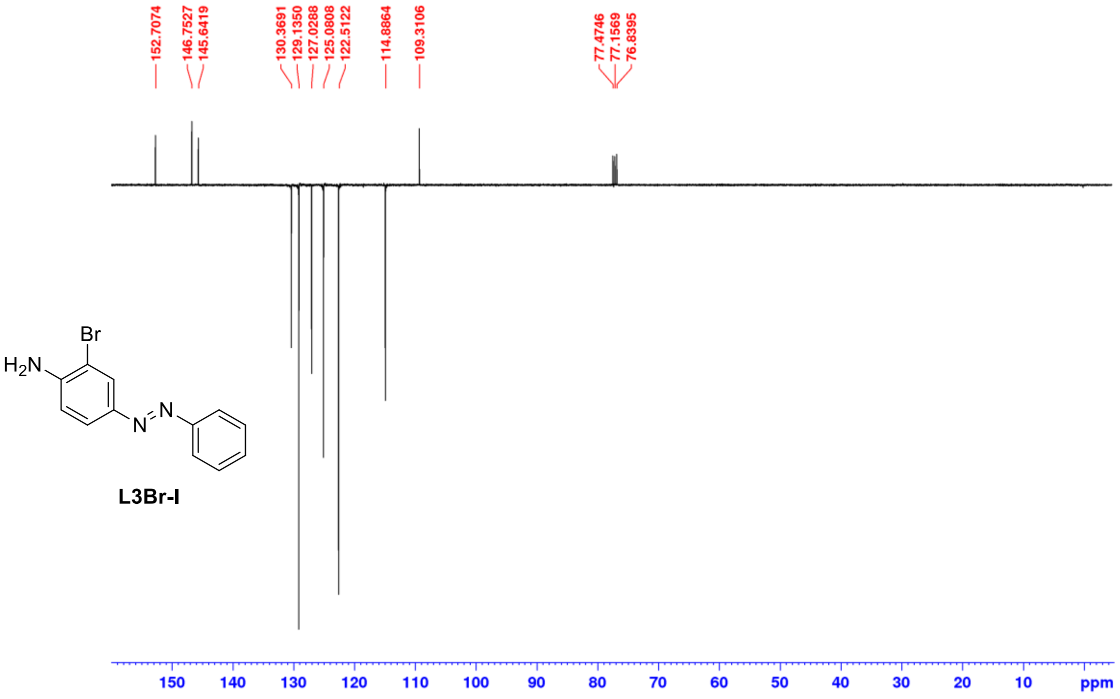

Figure S63: DEPTQ NMR spectrum of **L3Br-I** in $CDCl_3$ (101 MHz).

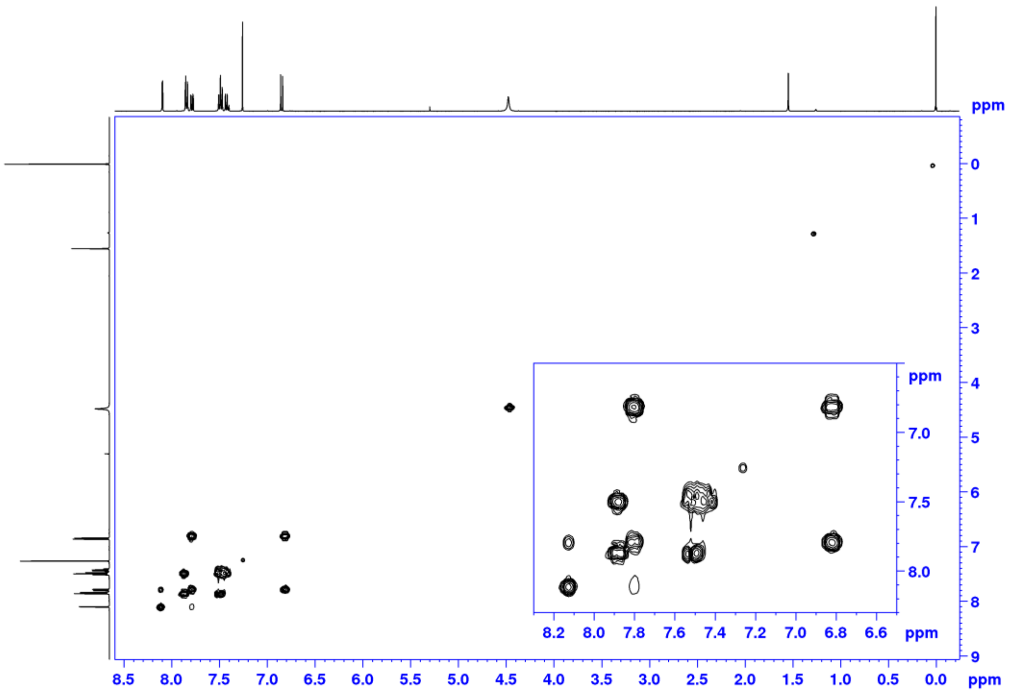

Figure S64: COSY spectrum of **L3Br-I** in $CDCl_3$ (400 MHz).

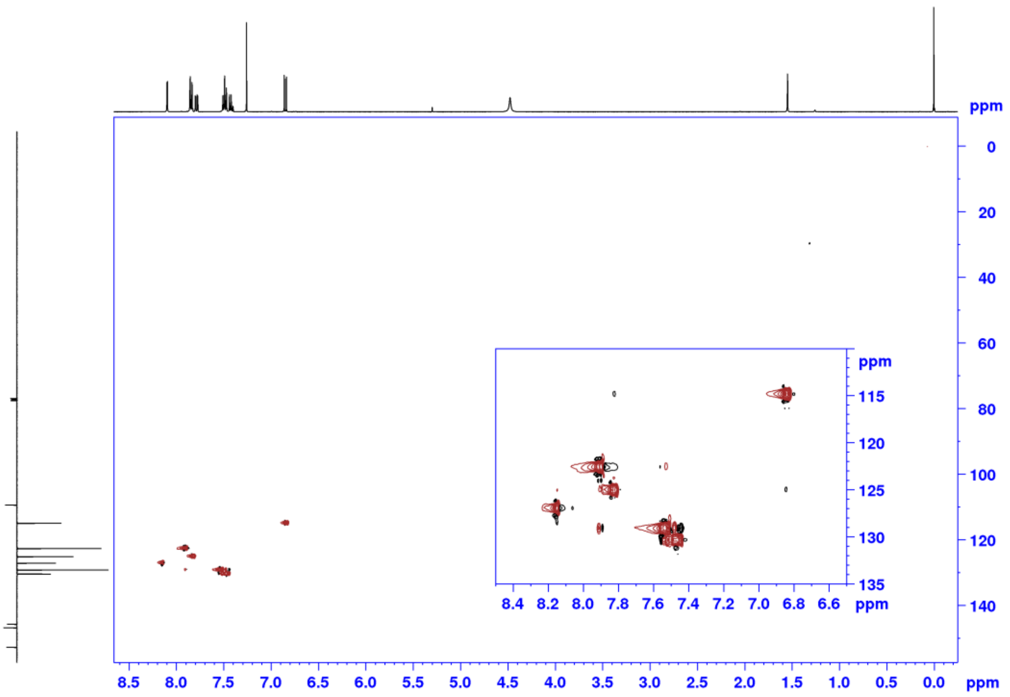

Figure S65: HSQC spectrum of **L3Br-I** in $CDCl_3$ (400 MHz).

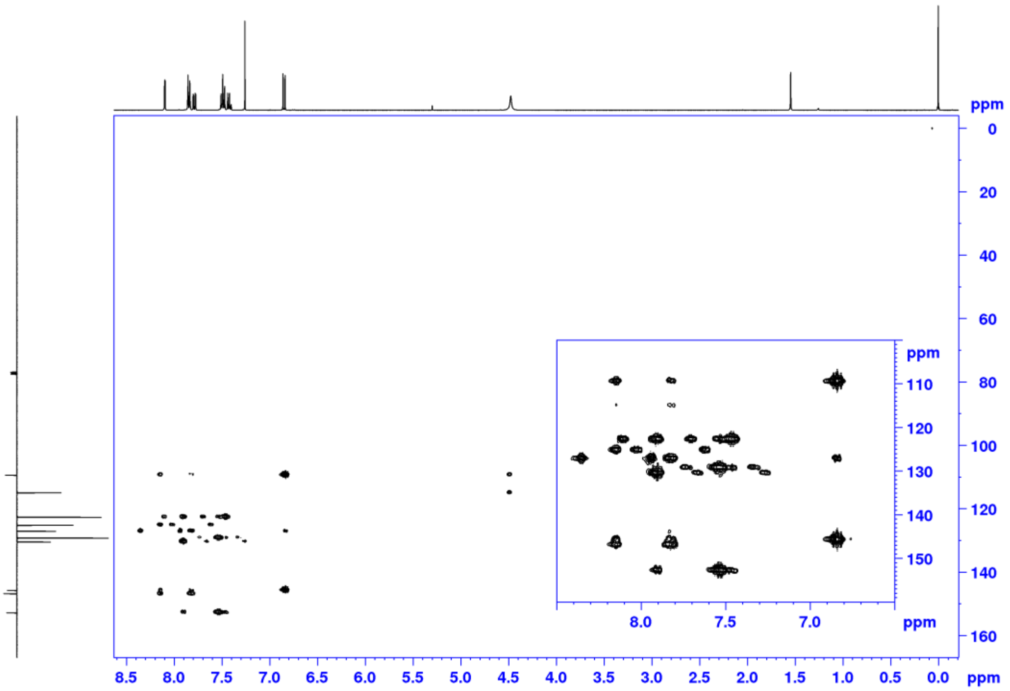

Figure S66: HMBC spectrum of **L3Br-I** in $CDCl_3$ (400 MHz).

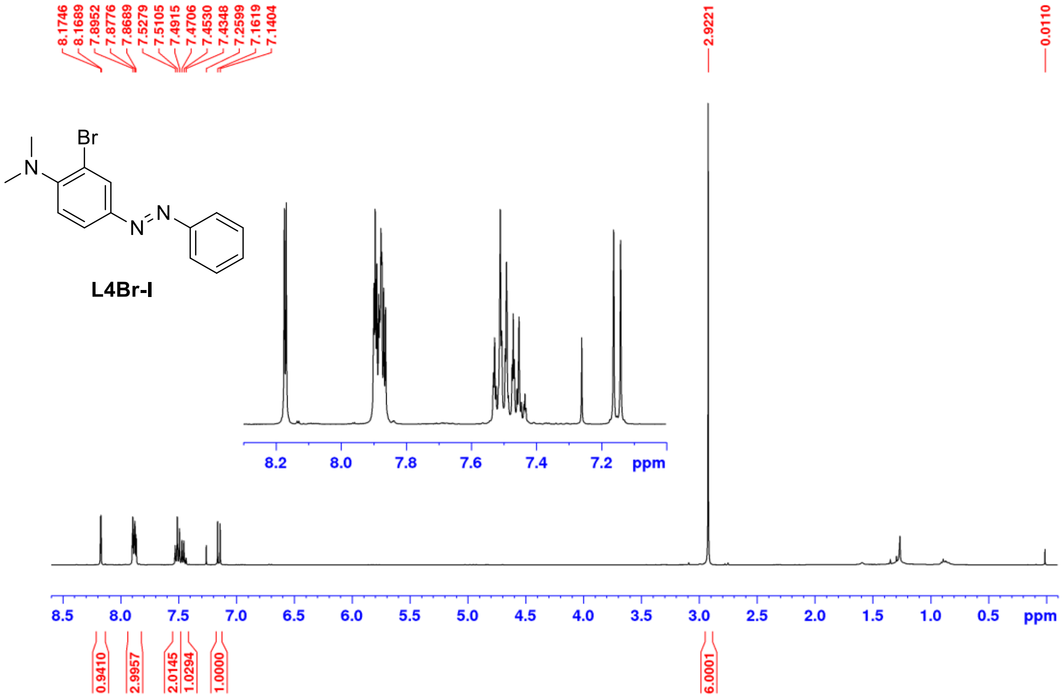

Figure S67: ^{1}H NMR spectrum of **L4Br-I** in $CDCl_3$ (400 MHz).

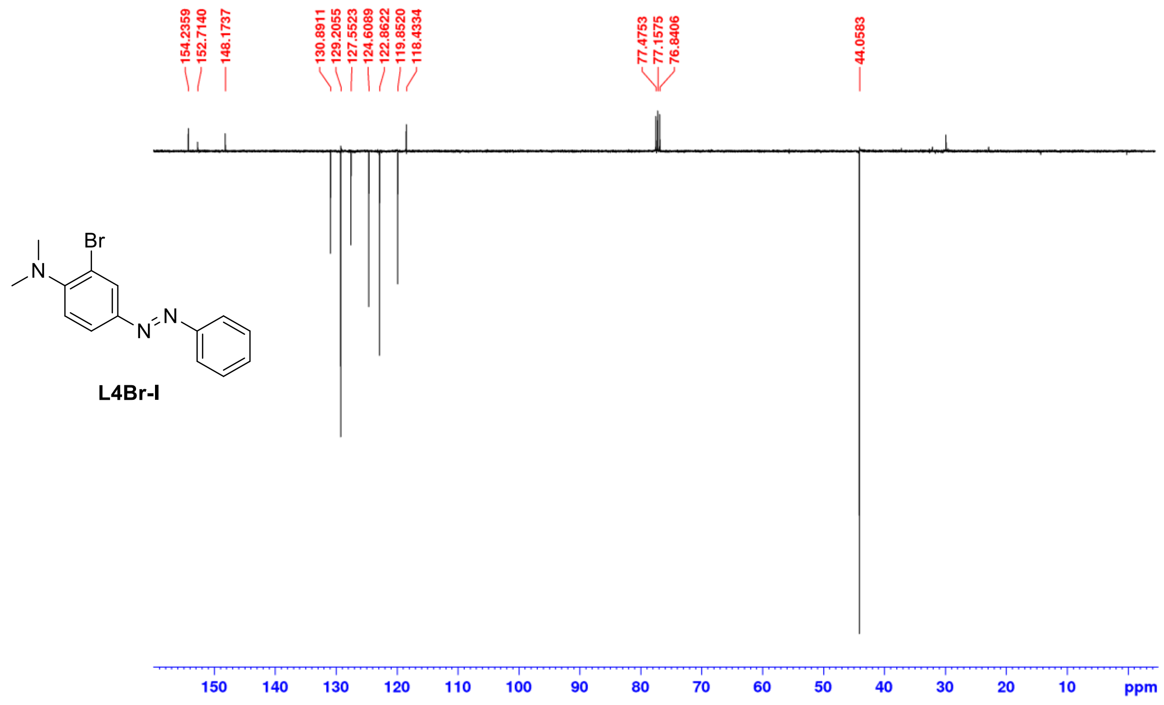

Figure S68: DEPTQ NMR spectrum of **L4Br-I** in $CDCl_3$ (101 MHz).

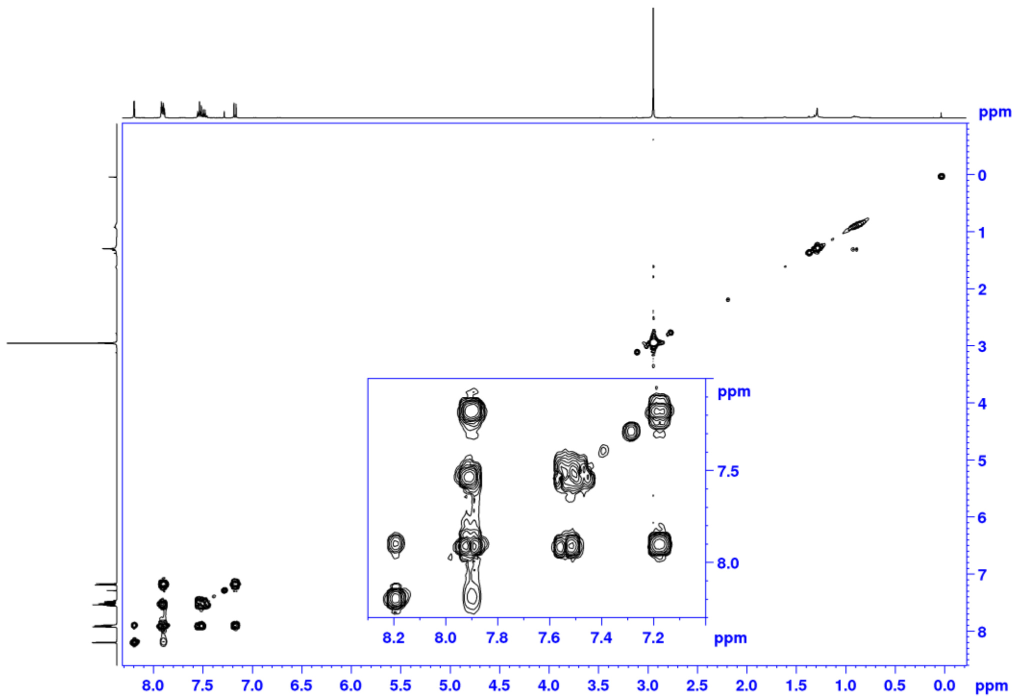

Figure S69: COSY spectrum of **L4Br-I** in $CDCl_3$ (400 MHz).

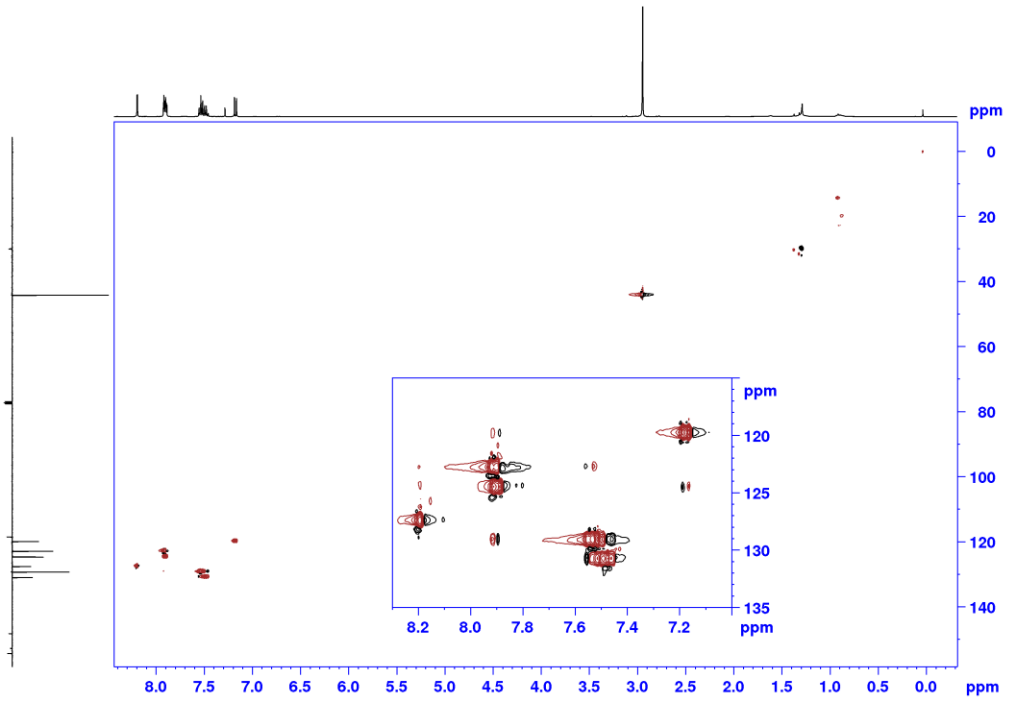

Figure S70: HSQC spectrum of **L4Br-I** in $CDCl_3$ (400 MHz).

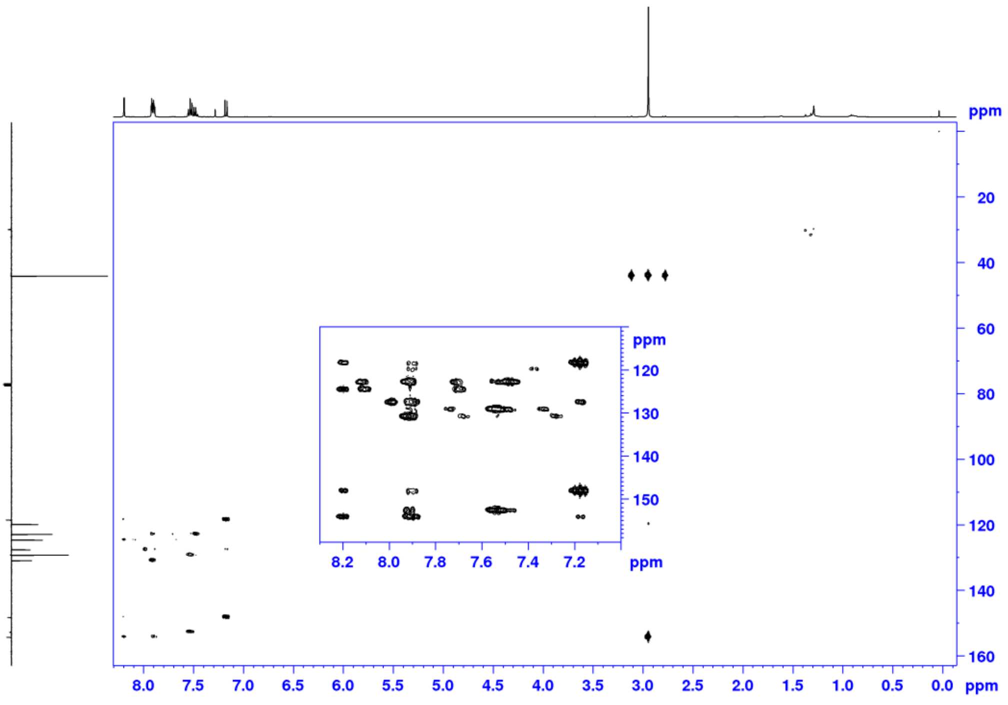

Figure S71: HMBC spectrum of **L4Br-I** in $CDCl_3$ (400 MHz).

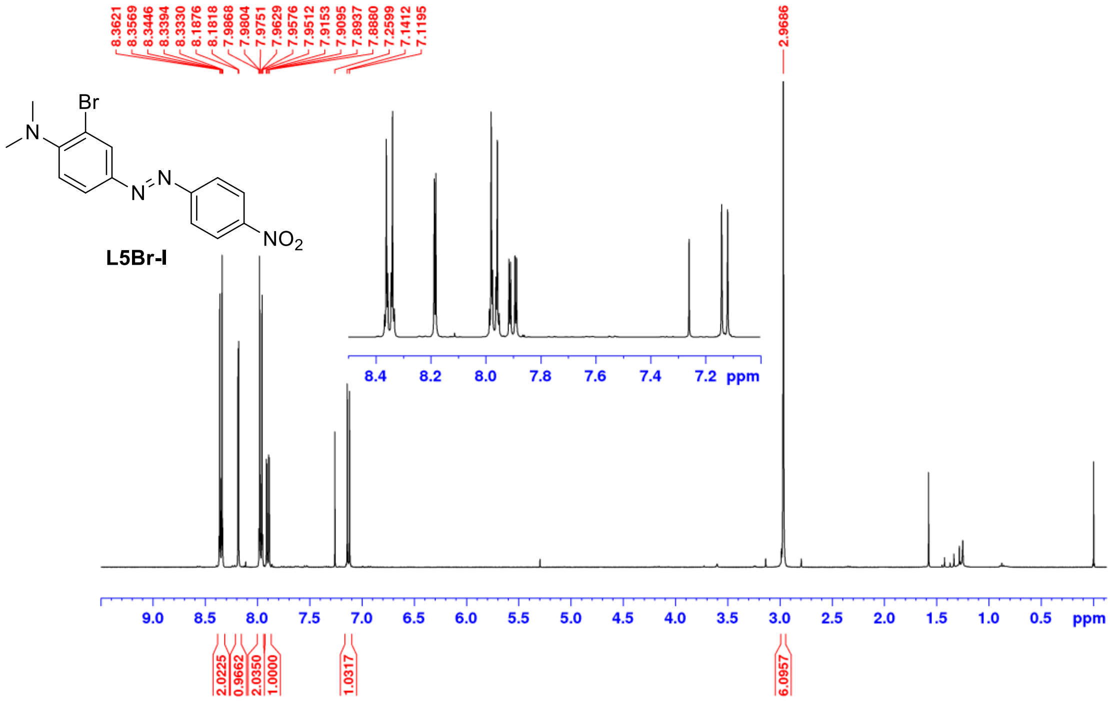

Figure S72: ^{1}H NMR spectrum of **L5Br-I** in $CDCl_3$ (400 MHz).

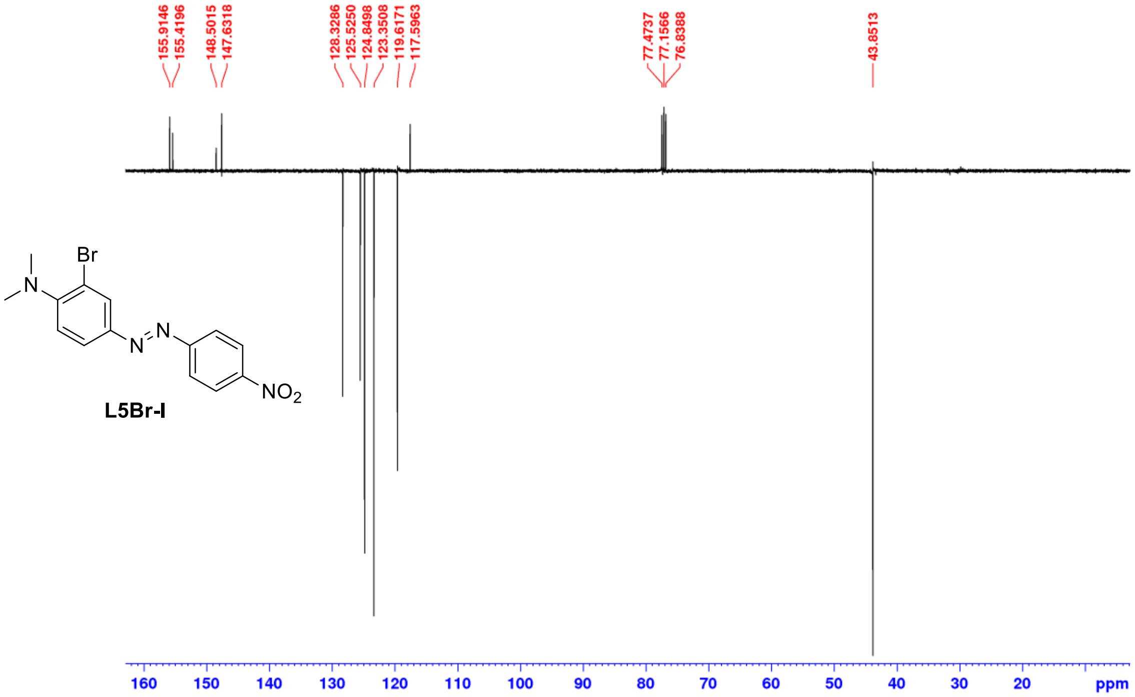

Figure S73: DEPTQ NMR spectrum of **L5Br-I** in $CDCl_3$ (101 MHz).

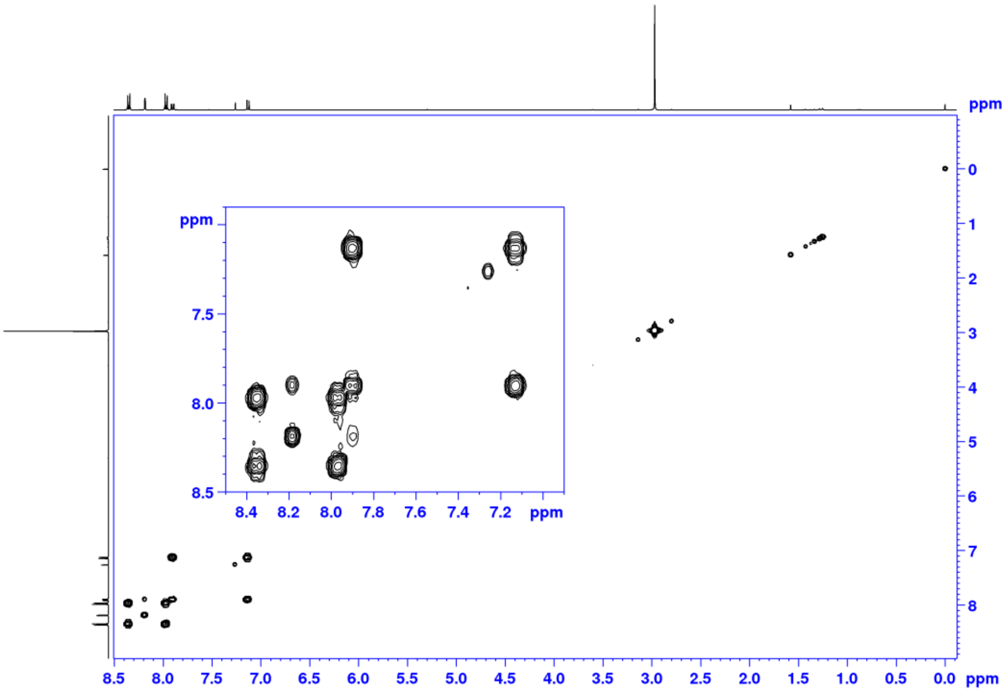

Figure S74: COSY spectrum of **L5Br-I** in $CDCl_3$ (400 MHz).

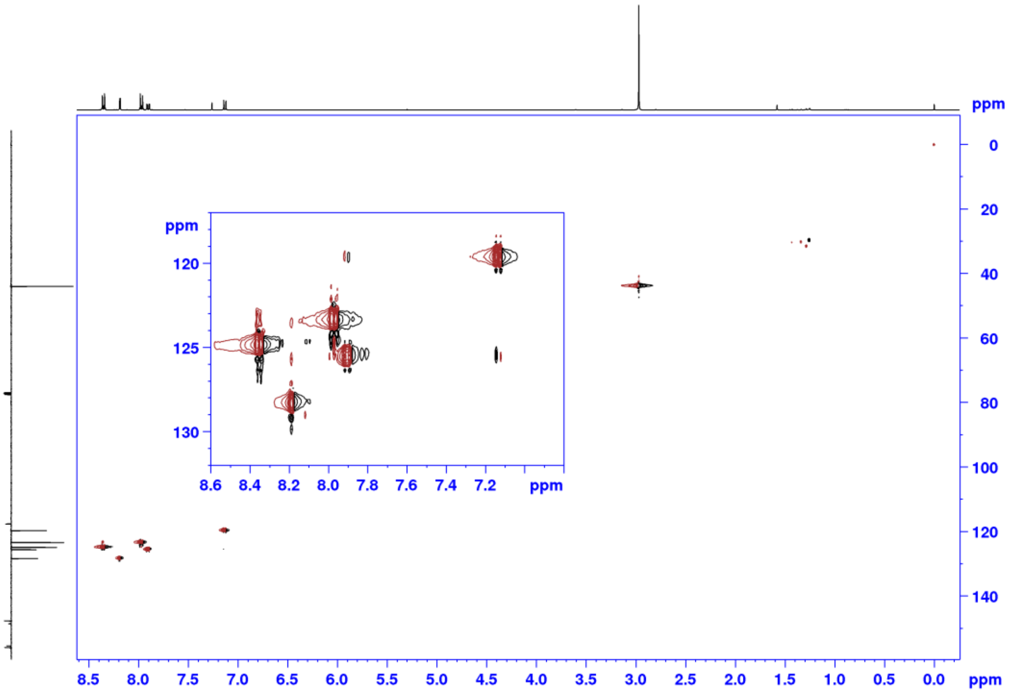

Figure S75: HSQC spectrum of **L5Br-I** in $CDCl_3$ (400 MHz).

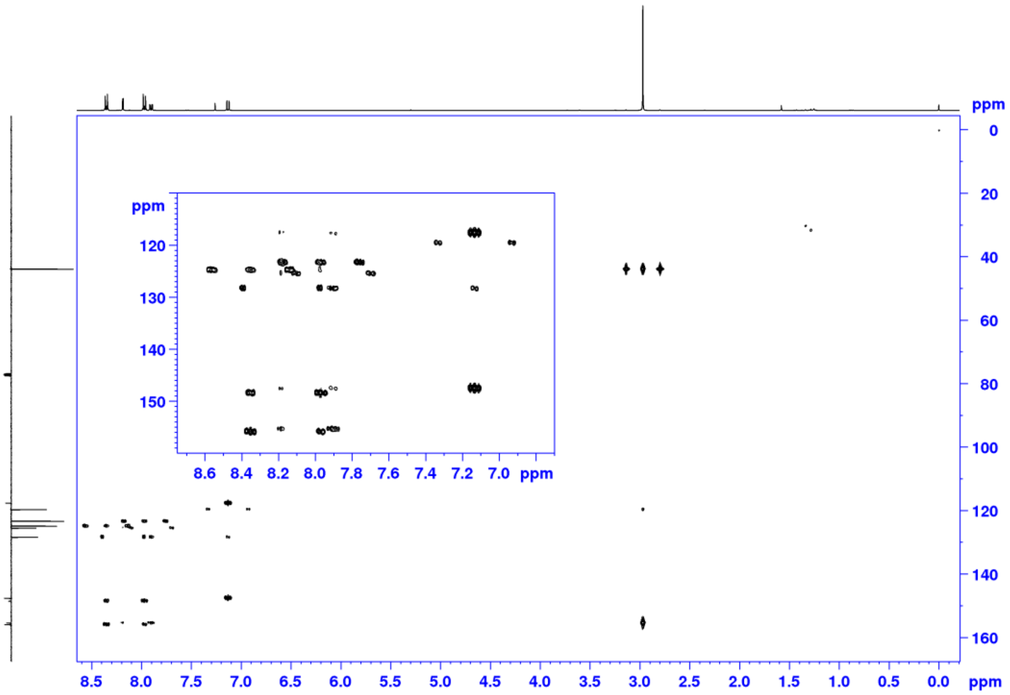

Figure S76: HMBC spectrum of **L5Br-I** in $CDCl_3$ (400 MHz).

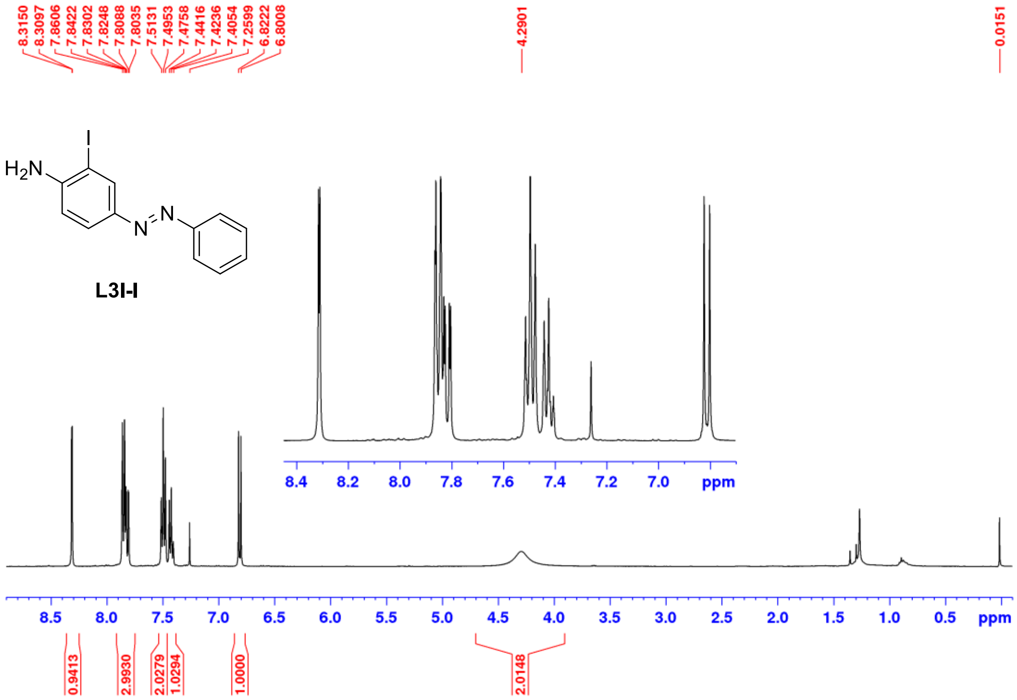

Figure S77: ^{1}H NMR spectrum of **L3I-I** in $CDCl_3$ (400 MHz).

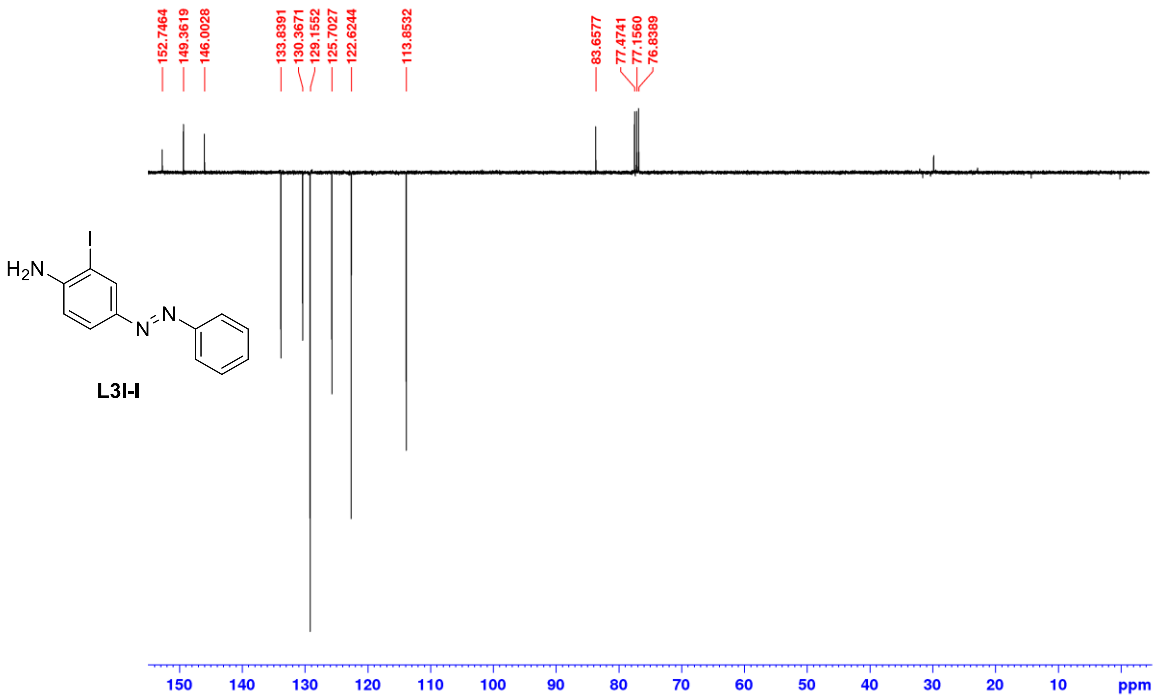

Figure S78: DEPTQ NMR spectrum of **L3I-I** in $CDCl_3$ (101 MHz).

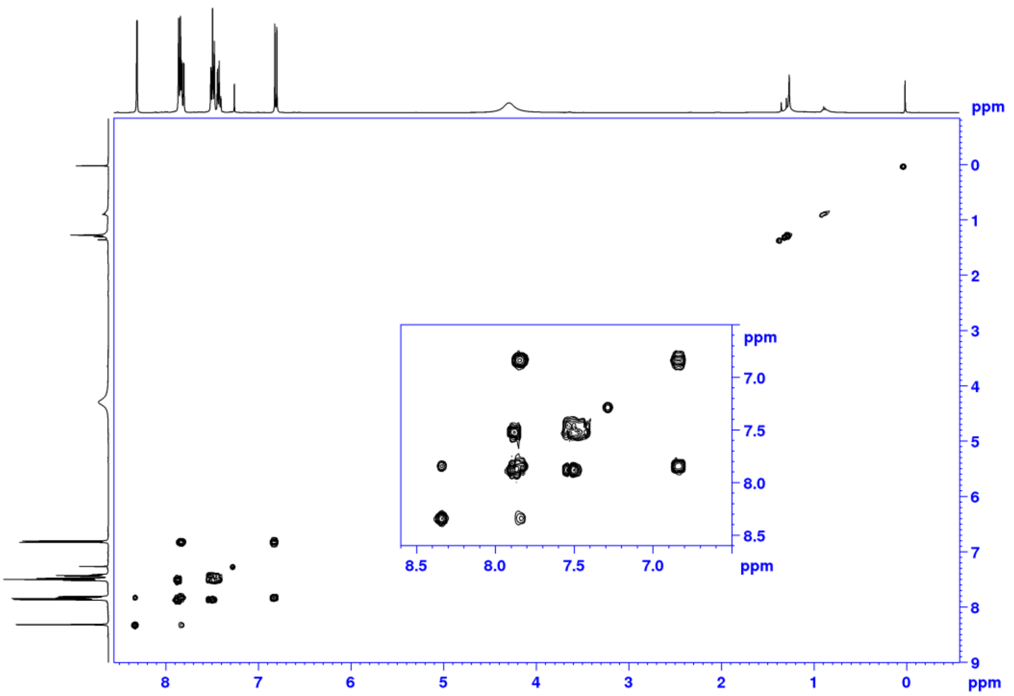

Figure S79: COSY spectrum of **L3I-I** in $CDCl_3$ (400 MHz).

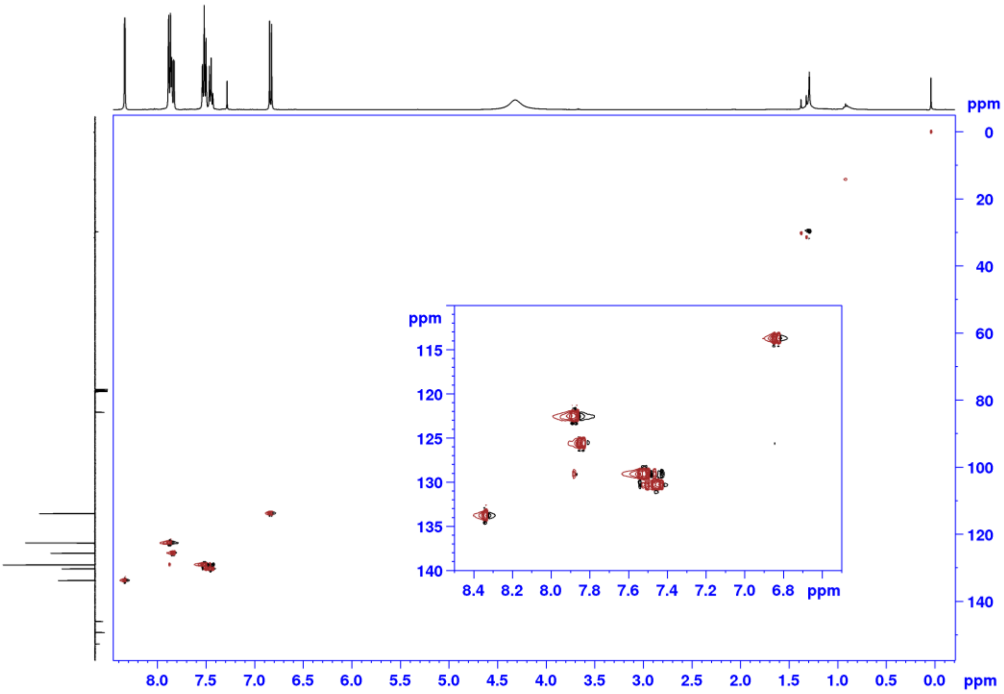

Figure S80: HSQC spectrum of **L3I-I** in $CDCl_3$ (400 MHz).

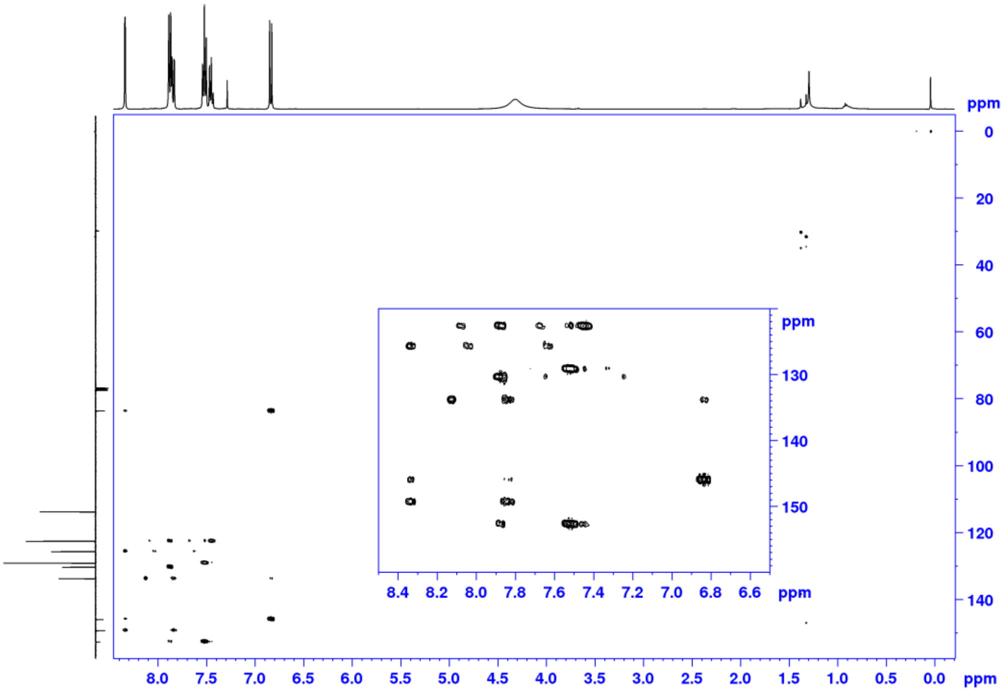

Figure S81: HMBC spectrum of **L3I-I** in $CDCl_3$ (400 MHz).

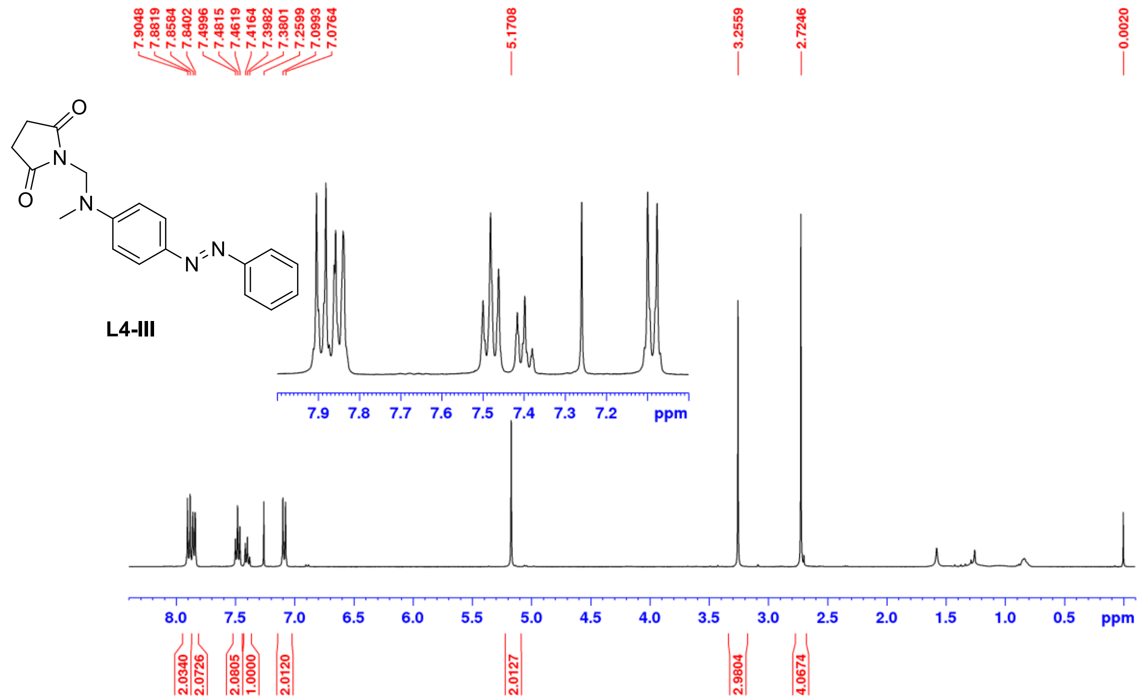

Figure S82: ^{1}H NMR spectrum of **L4-III** in $CDCl_3$ (400 MHz).

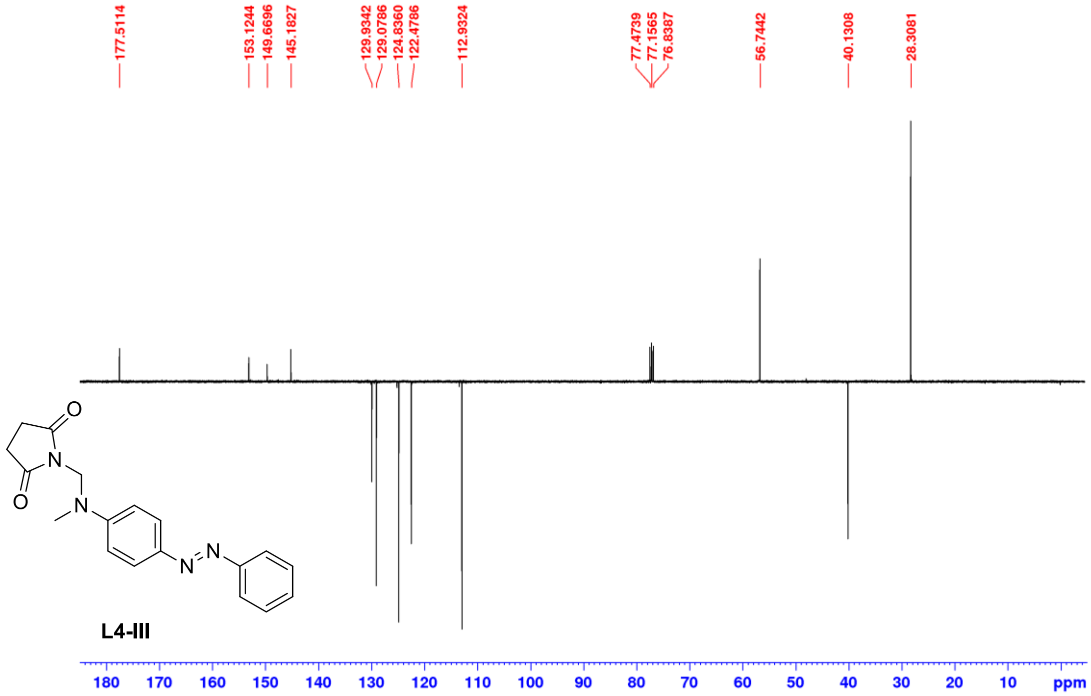

Figure S83: DEPTQ spectrum of **L4-III** in $CDCl_3$ (400 MHz).

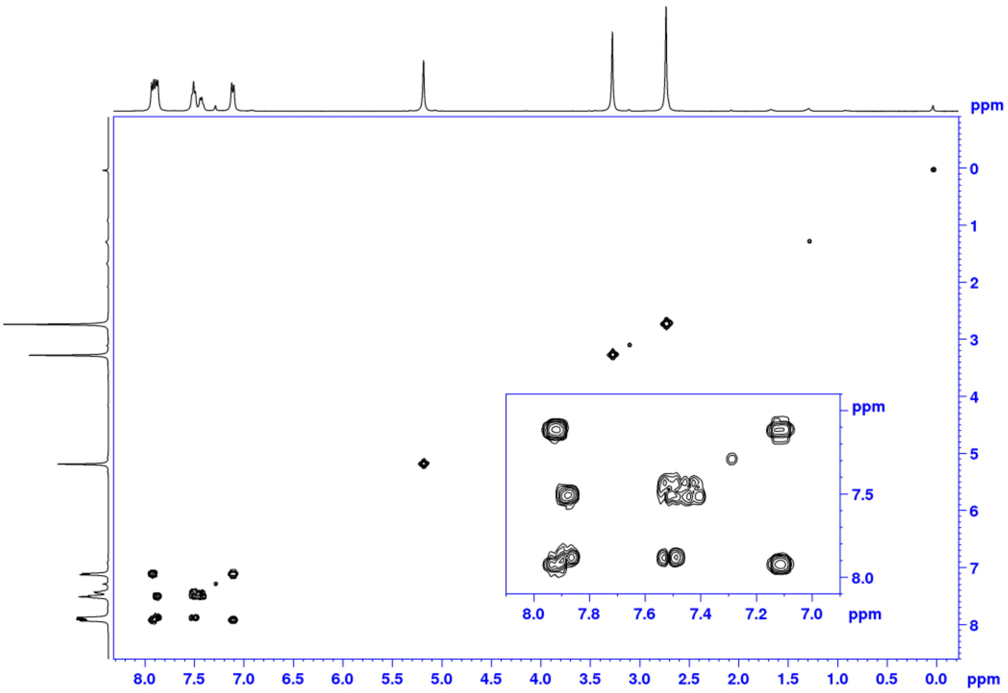

Figure S84: COSY spectrum of **L4-III** in $CDCl_3$ (400 MHz).

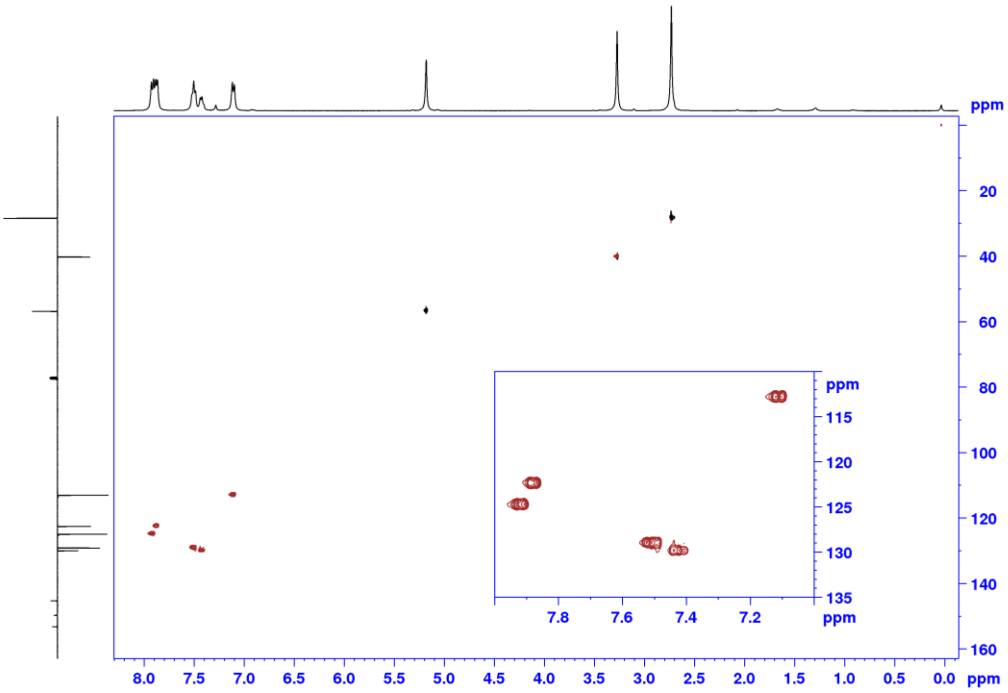

Figure S85: HSQC spectrum of **L4-III** in $CDCl_3$ (400 MHz).

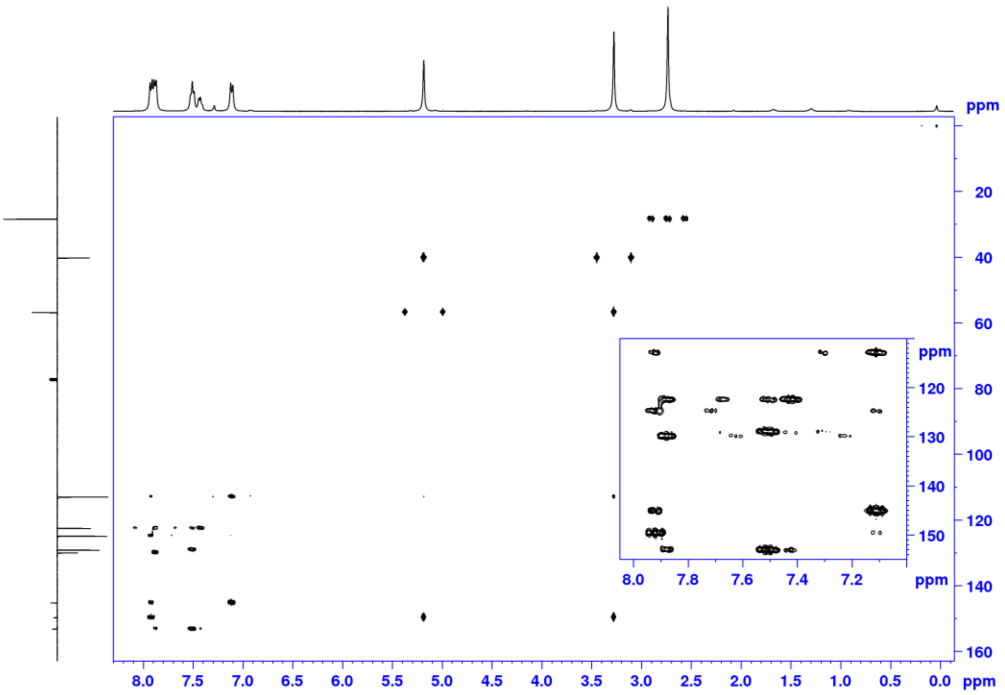

Figure S86: HMBC spectrum of **L4-III** in $CDCl_3$ (400 MHz).

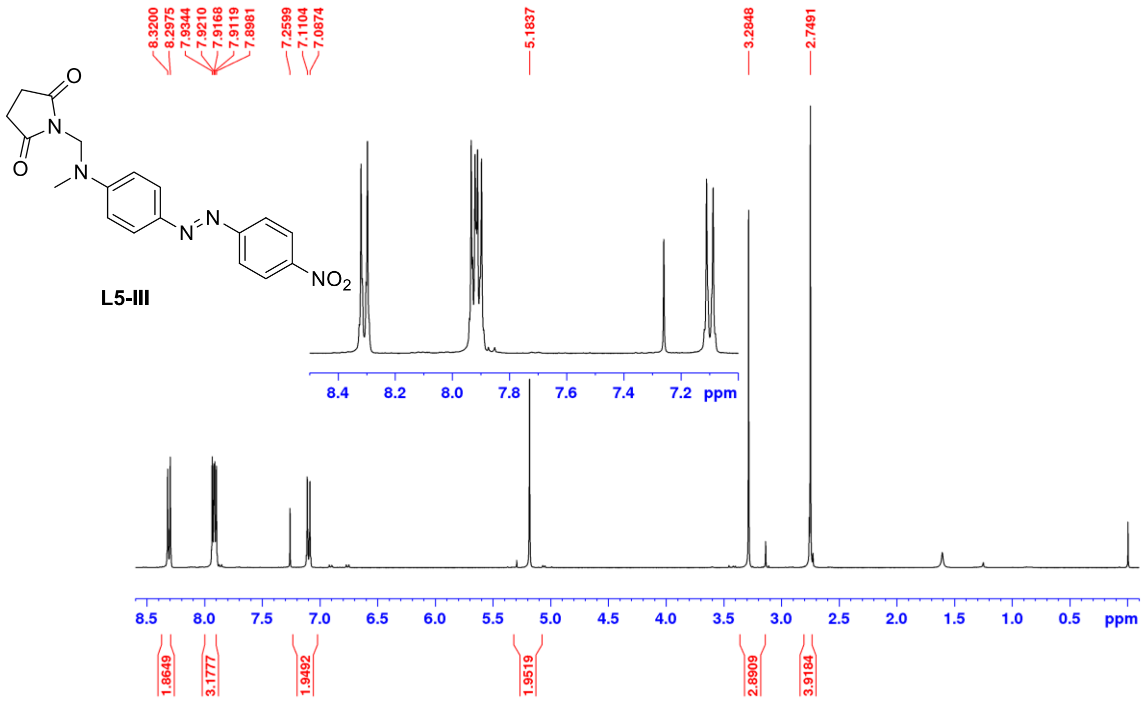

Figure S87: ^{1}H NMR spectrum of **L5-III** in $CDCl_3$ (400 MHz).

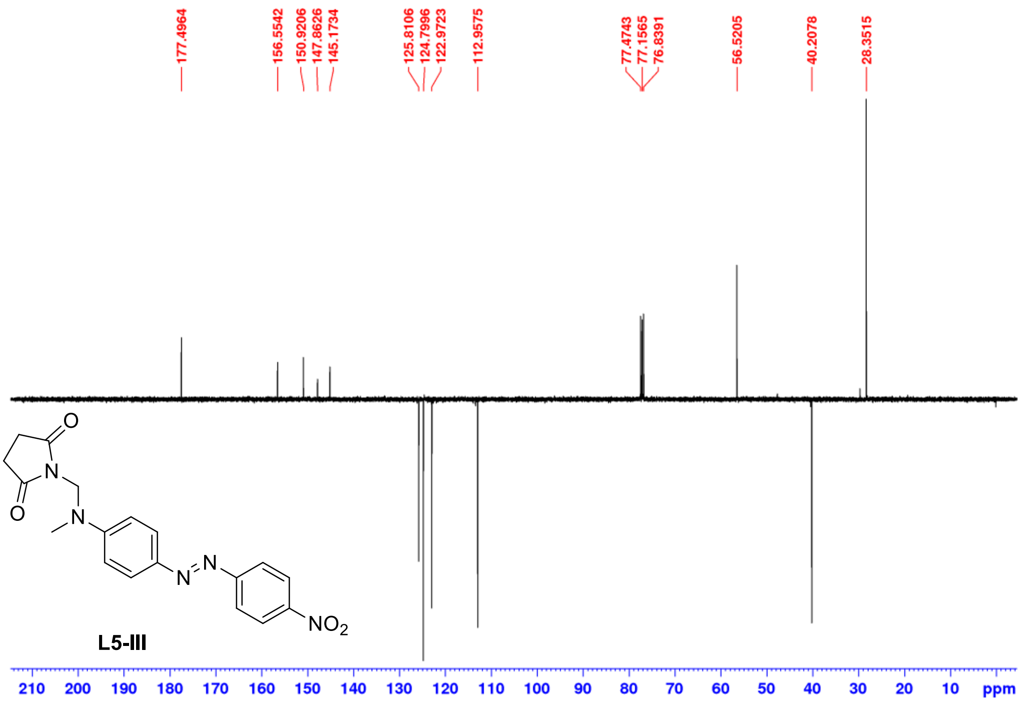

Figure S88: DEPTQ NMR spectrum of **L5-III** in $CDCl_3$ (101 MHz).

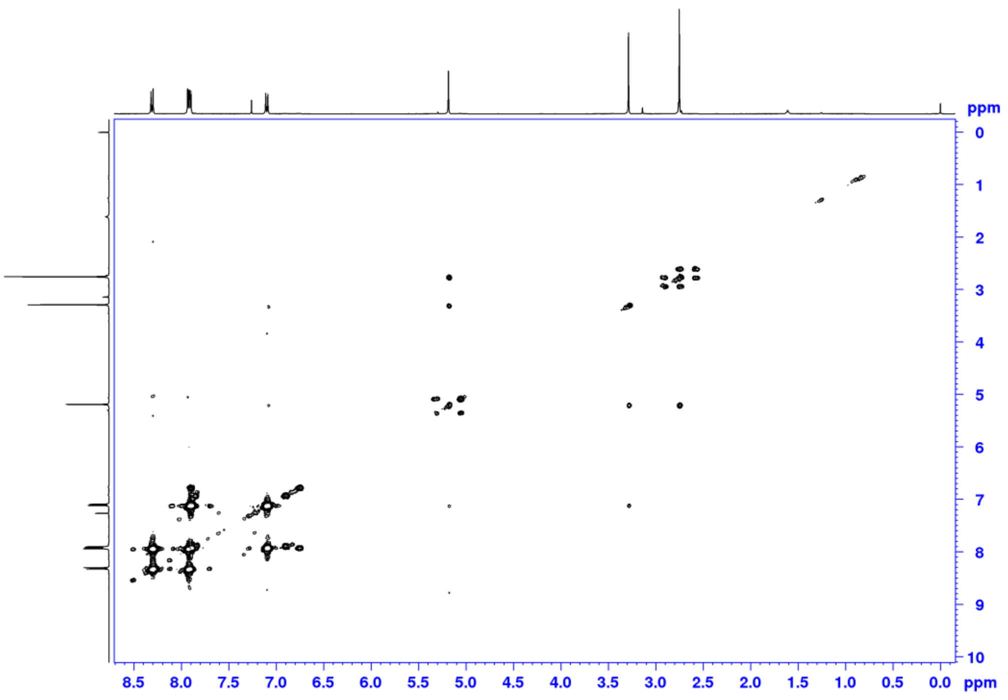

Figure S89: COSY spectrum of **L5-III** in $CDCl_3$ (400 MHz).

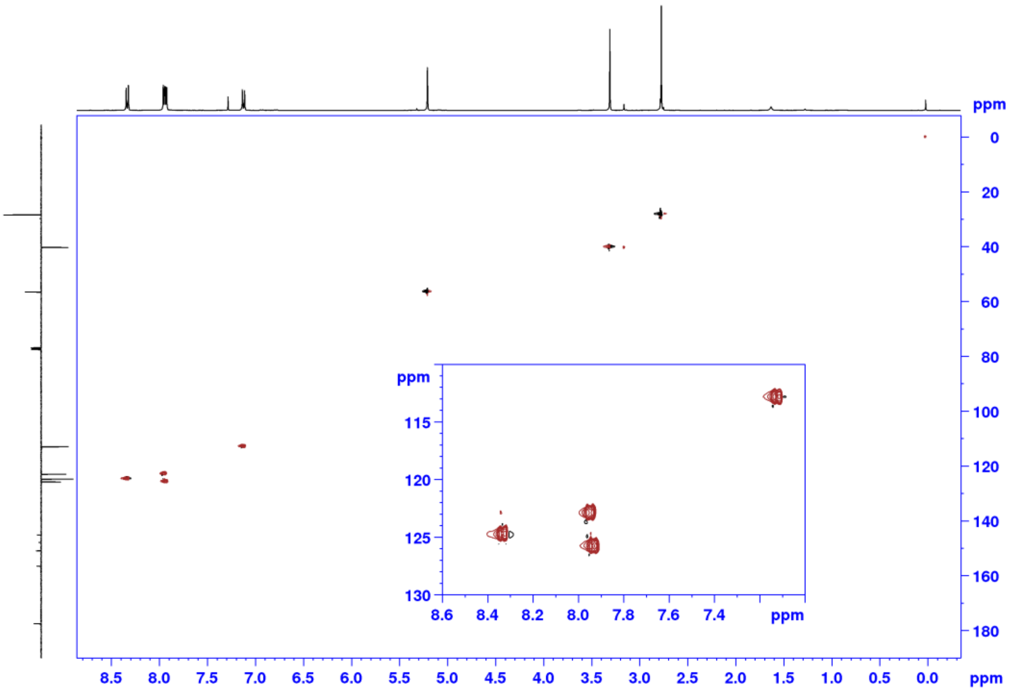

Figure S90: HSQC spectrum of **L5-III** in $CDCl_3$ (400 MHz).

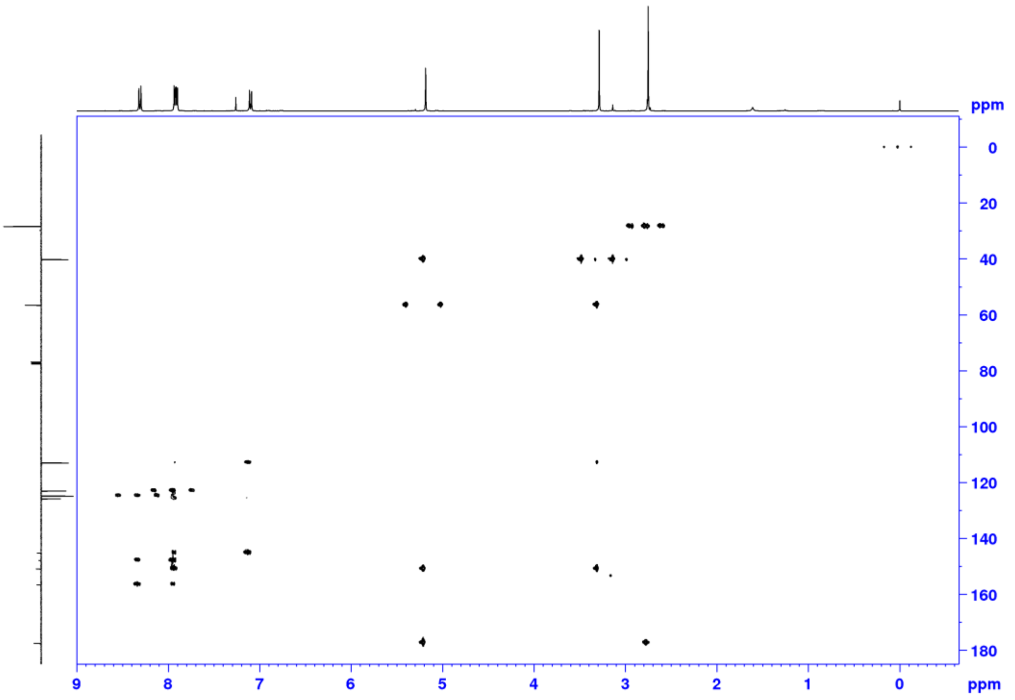

Figure S91: HMBC spectrum of **L5-III** in $CDCl_3$ (400 MHz).

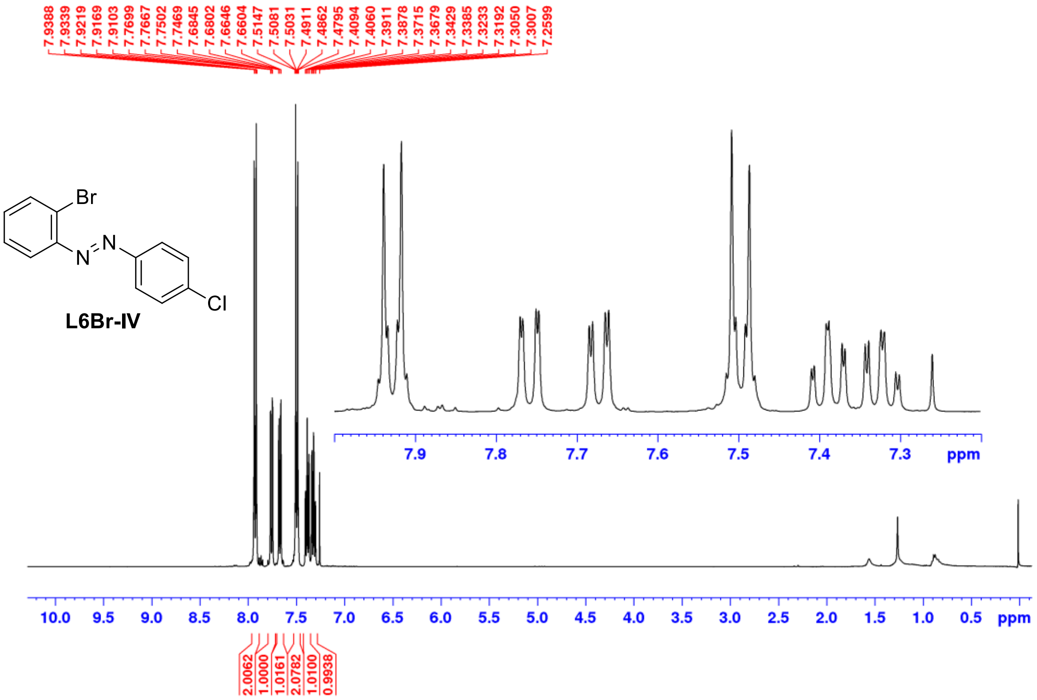

Figure S92: ^{1}H NMR spectrum of **L6Br-IV** in $CDCl_3$ (400 MHz).

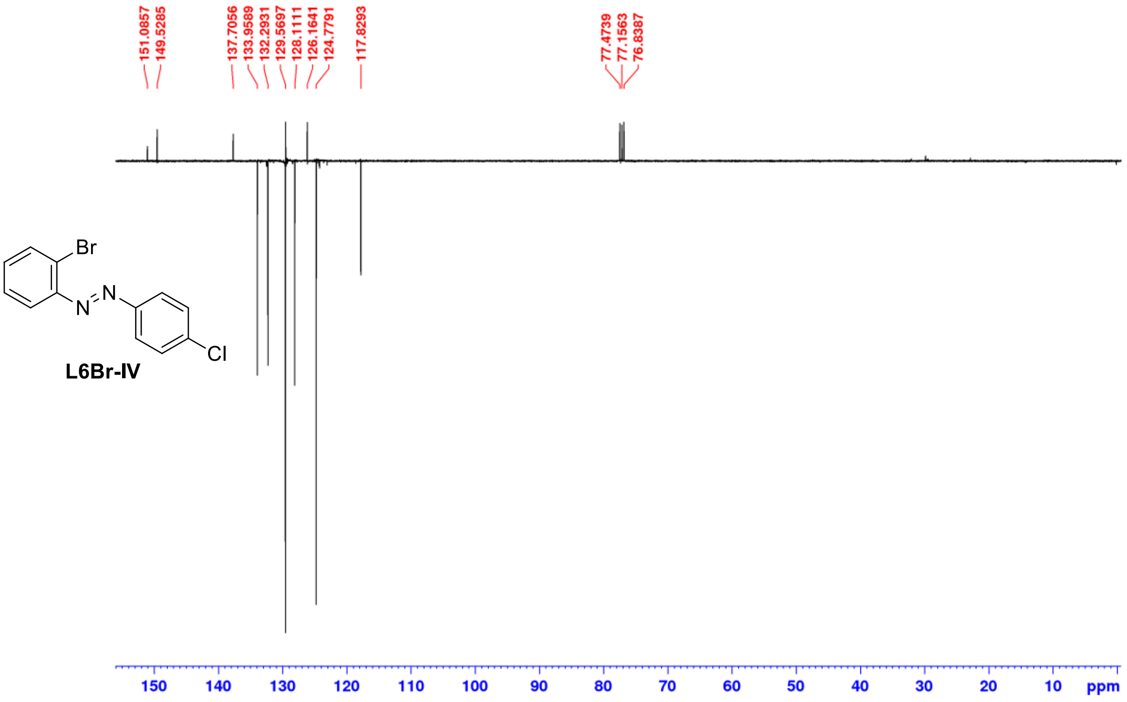

Figure S93: DEPTQ NMR spectrum of **L6Br-IV** in $CDCl_3$ (101 MHz).

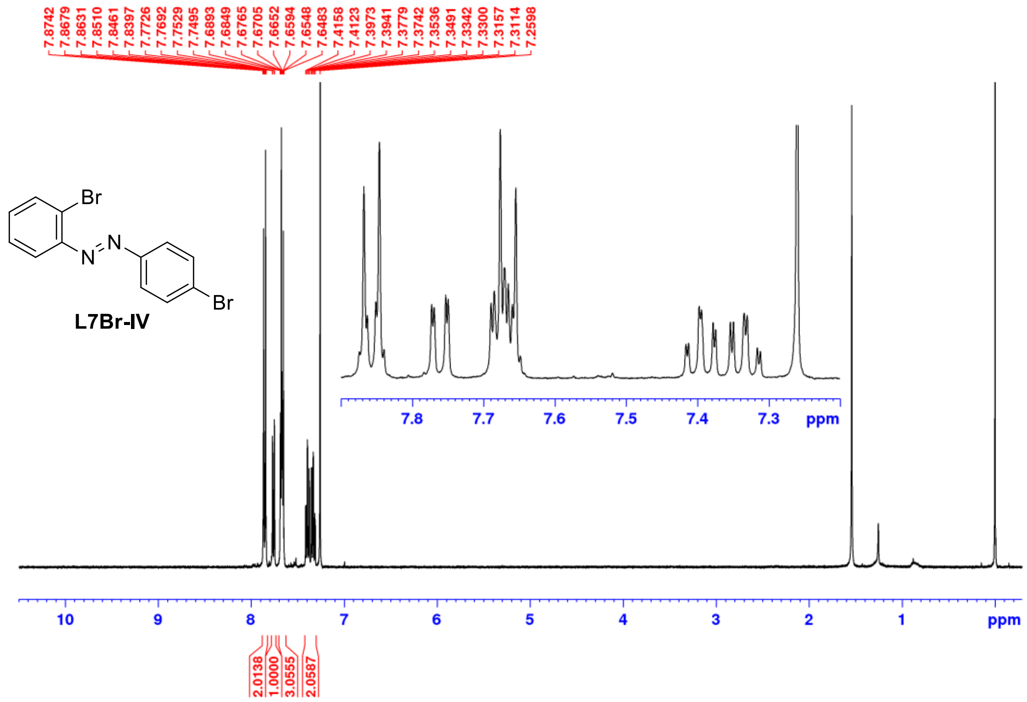

Figure S94: ^{1}H NMR spectrum of **L7Br-IV** in $CDCl_3$ (400 MHz).

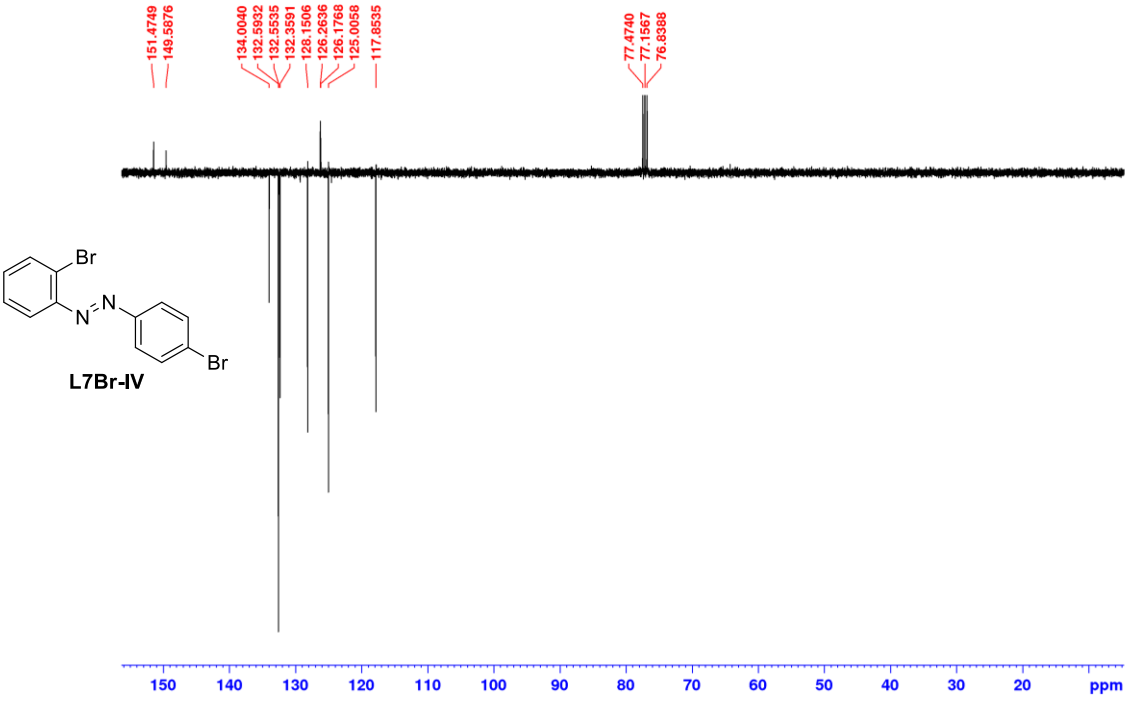

Figure S95: DEPTQ NMR spectrum of **L7Br-IV** in $CDCl_3$ (101 MHz).

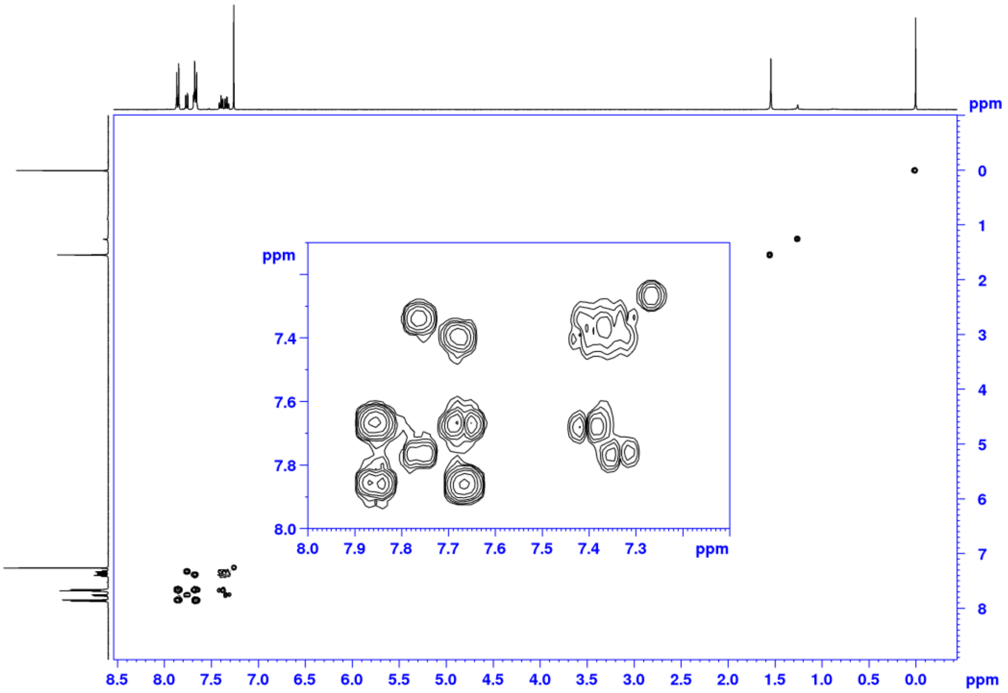

Figure S96: COSY spectrum of **L7Br-IV** in $CDCl_3$ (400 MHz).

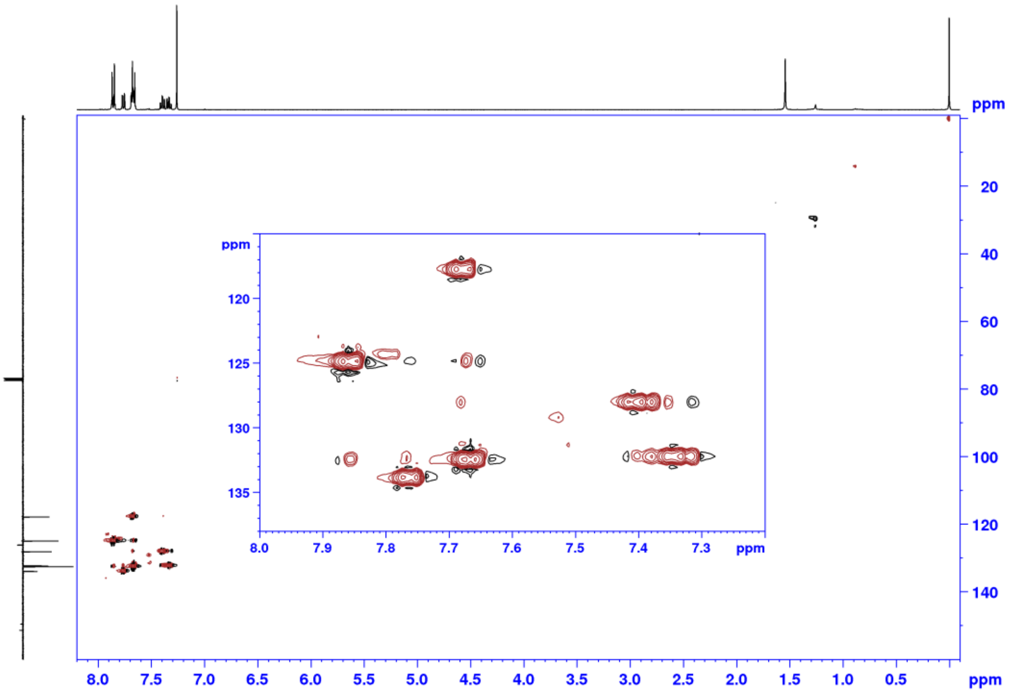

Figure S97: HSQC spectrum of **L7Br-IV** in $CDCl_3$ (400 MHz).

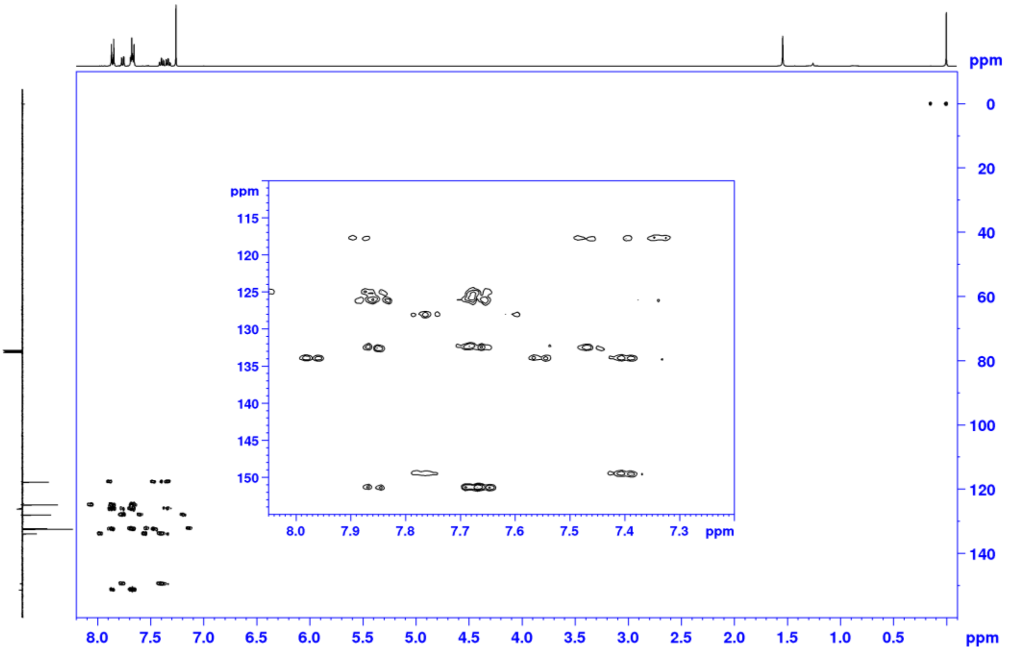

Figure S98: HMBC spectrum of **L7Br-IV** in $CDCl_3$ (400 MHz).

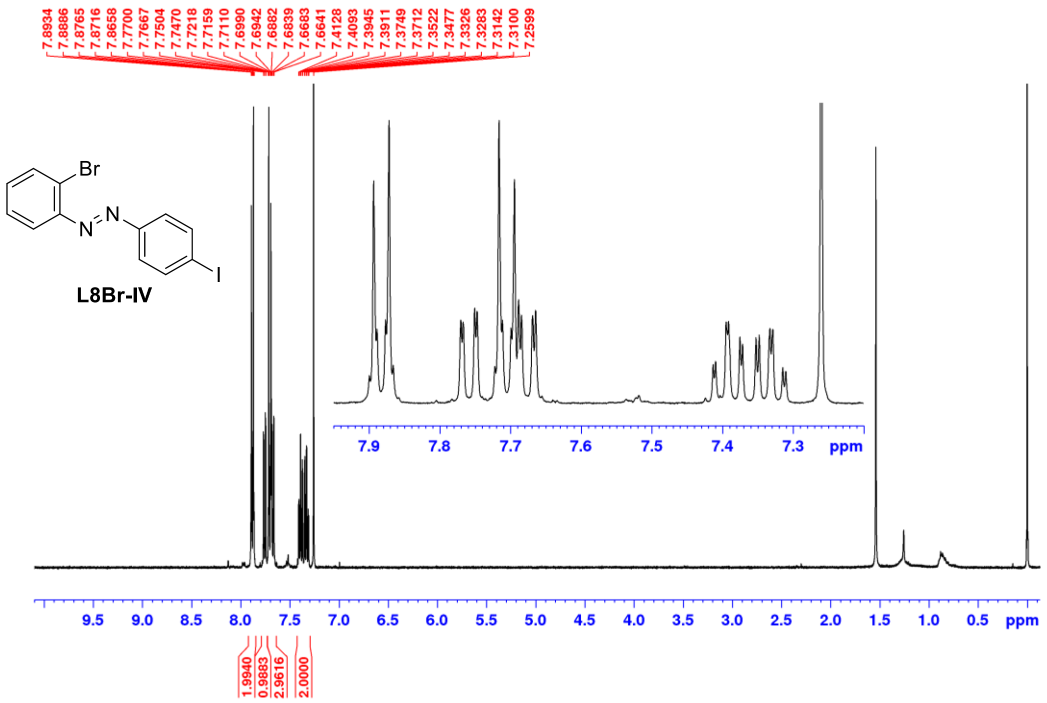

Figure S99: ^{1}H NMR spectrum of **L8Br-IV** in $CDCl_3$ (400 MHz).

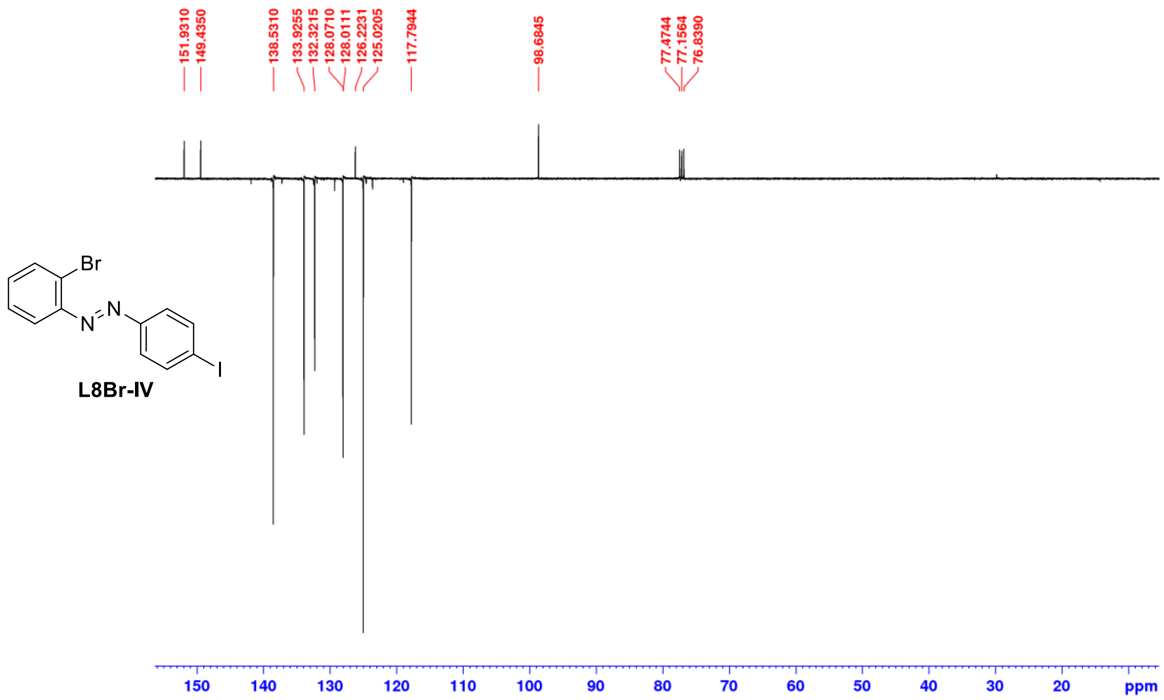

Figure S100: DEPTQ NMR spectrum of **L8Br-IV** in $CDCl_3$ (101 MHz).

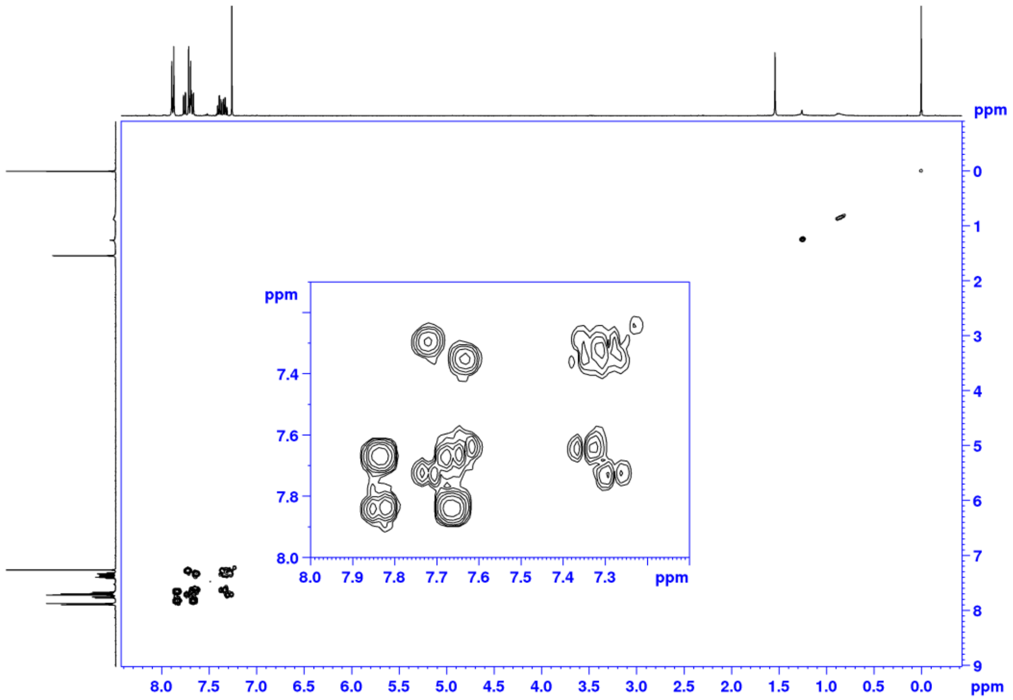

Figure S101: COSY spectrum of **L8Br-IV** in $CDCl_3$ (400 MHz).

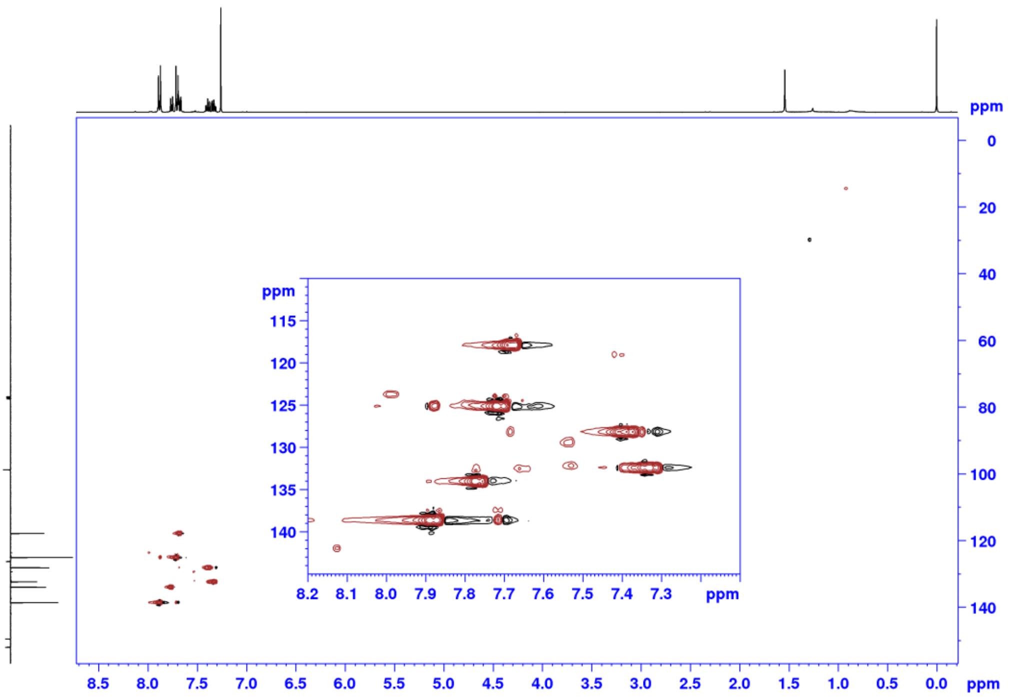

Figure S102: HSQC spectrum of **L8Br-IV** in $CDCl_3$ (400 MHz).

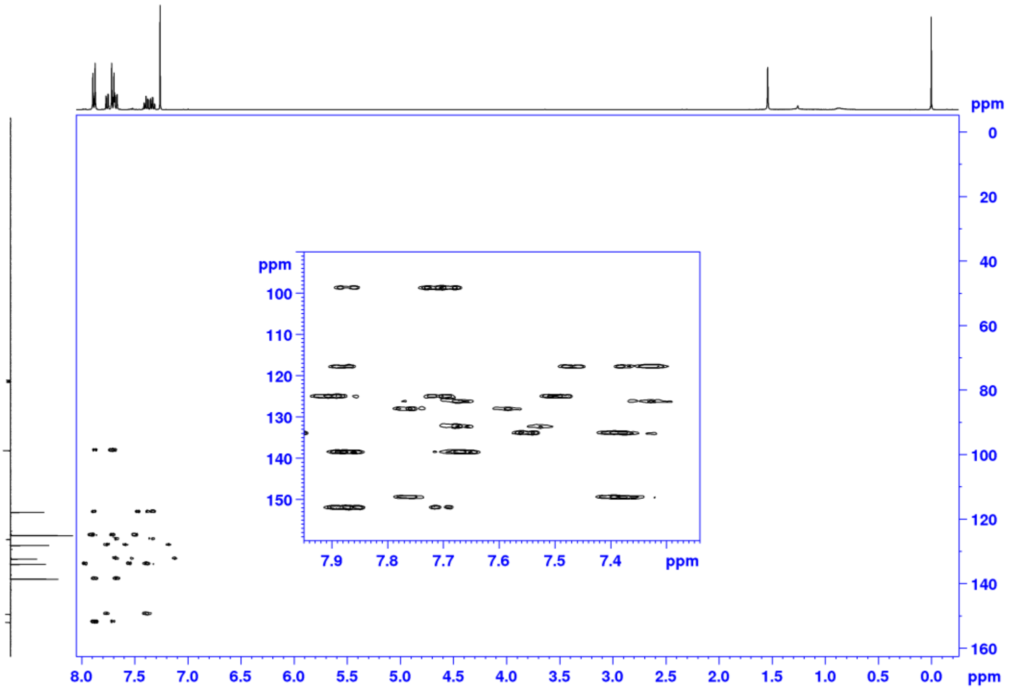

Figure S103: HMBC spectrum of **L8Br-IV** in $CDCl_3$ (400 MHz).

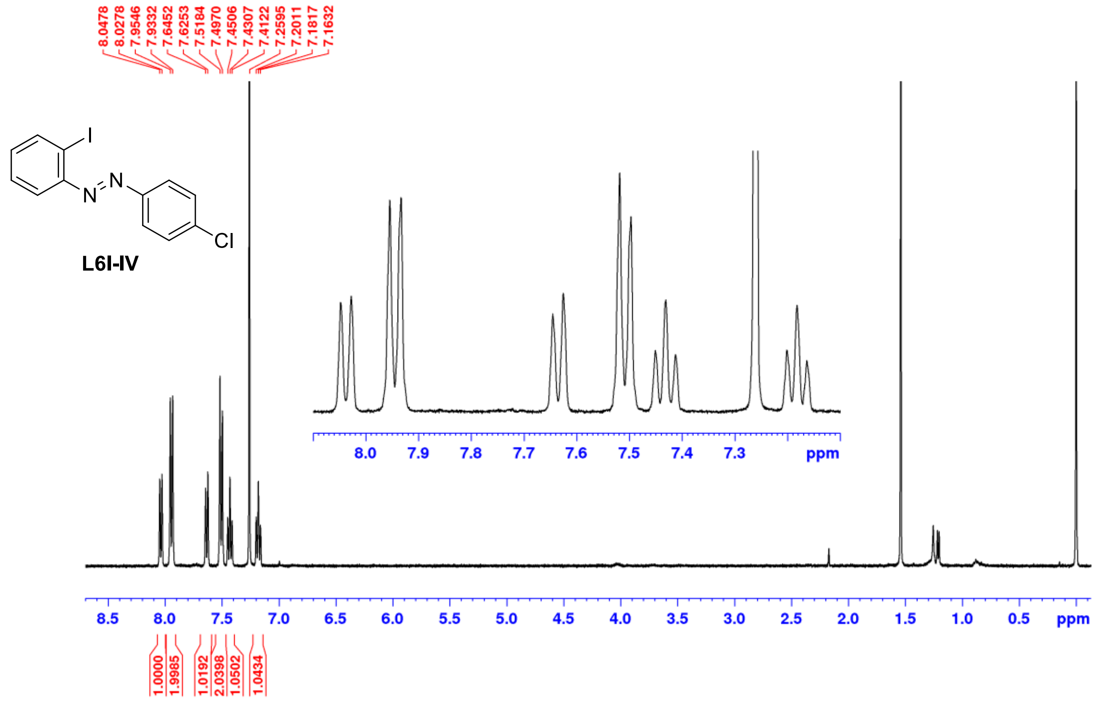

Figure S104: ^{1}H NMR spectrum of **L6I-IV** in $CDCl_3$ (400 MHz).

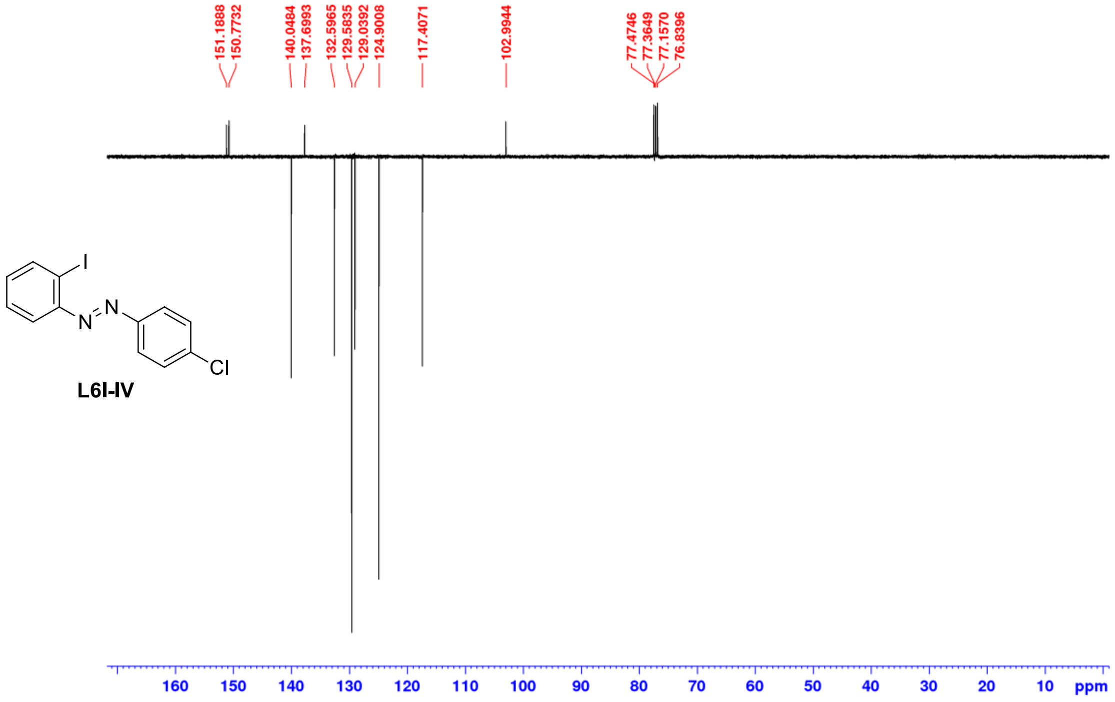

Figure S105: DEPTQ NMR spectrum of **L6I-IV** in $CDCl_3$ (101 MHz).

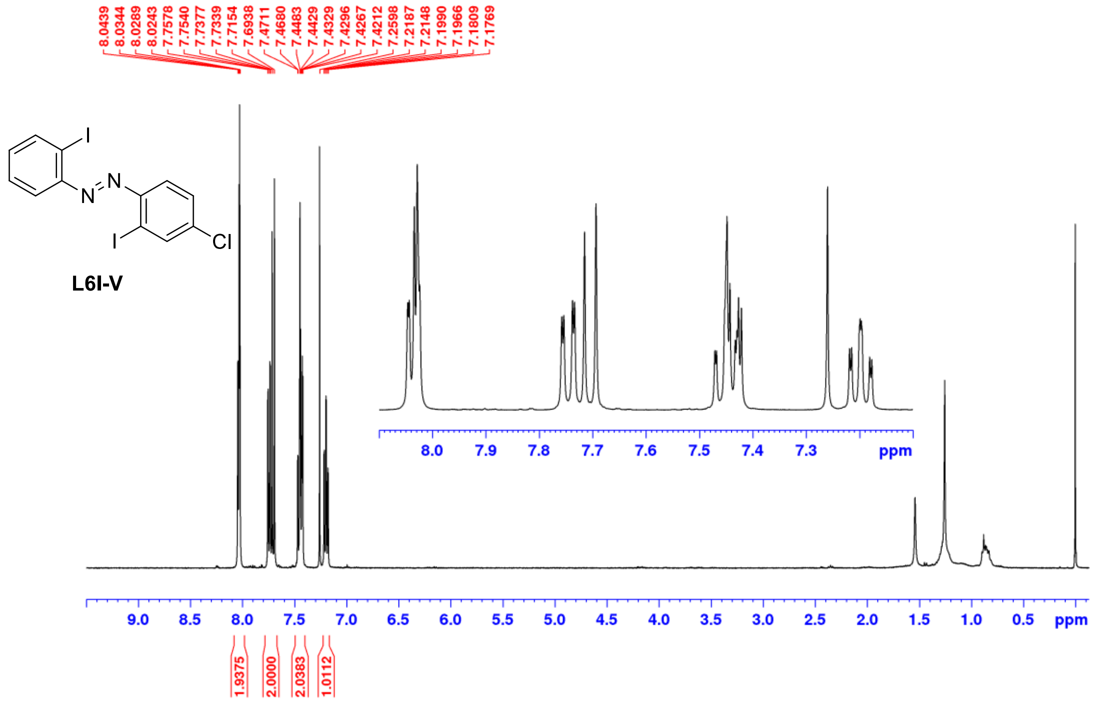

Figure S106: ^{1}H NMR spectrum of **L6I-V** in $CDCl_3$ (400 MHz).

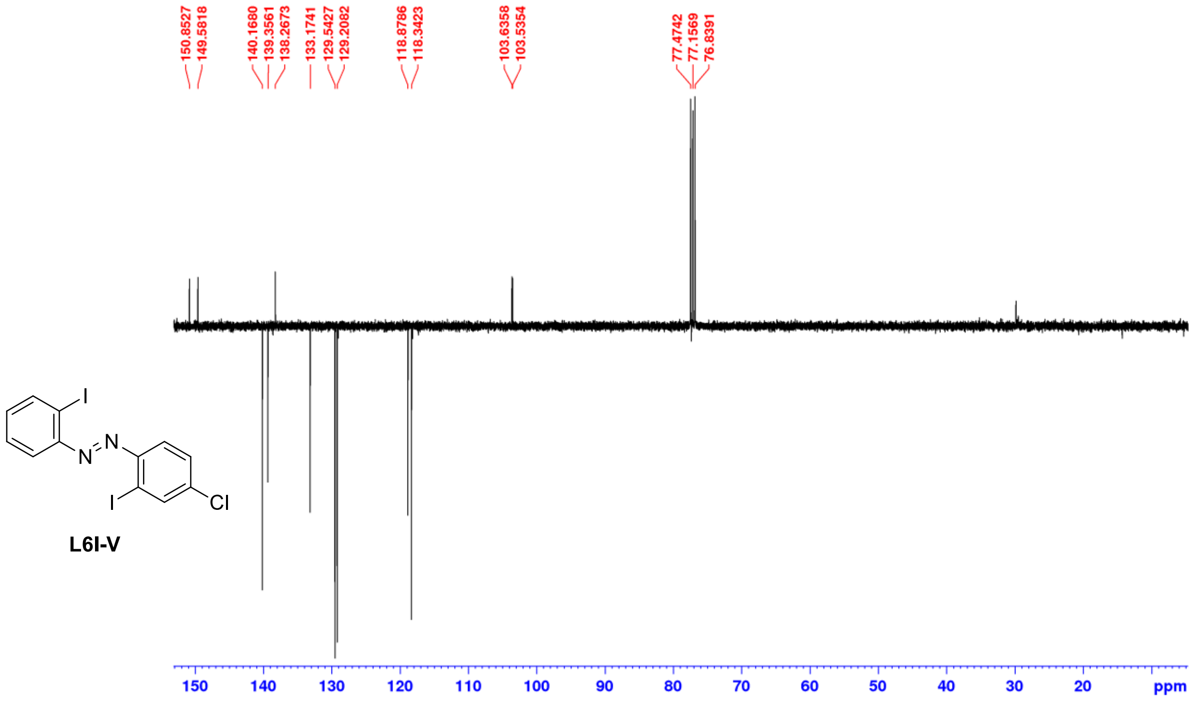

Figure S107: DEPTQ NMR spectrum of **L6I-V** in $CDCl_3$ (101 MHz).

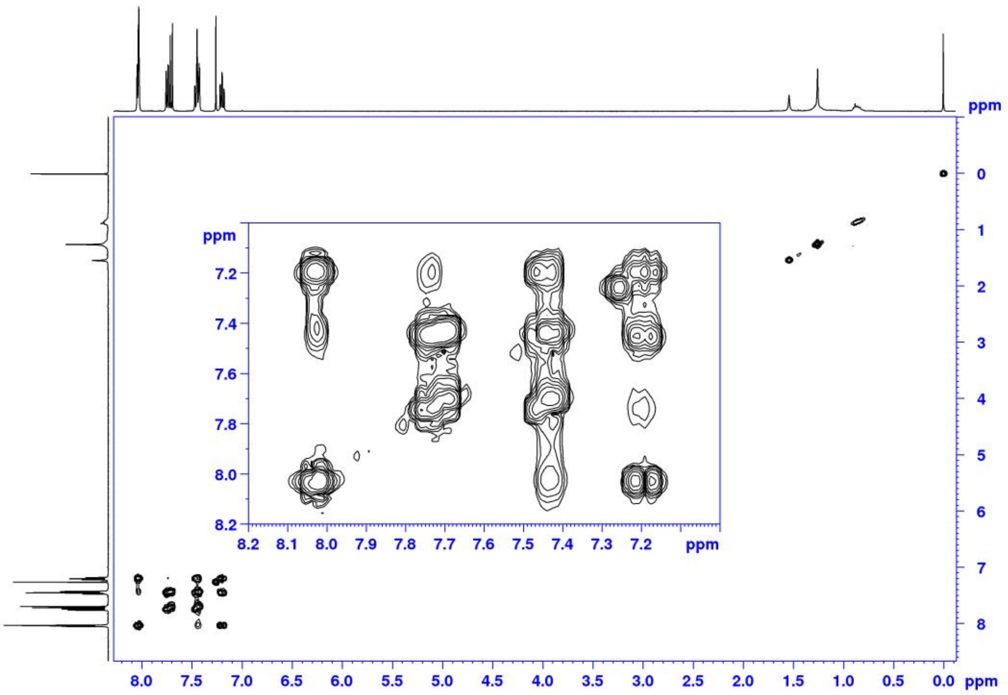

Figure S108: COSY spectrum of **L6I-V** in $CDCl_3$ (400 MHz).

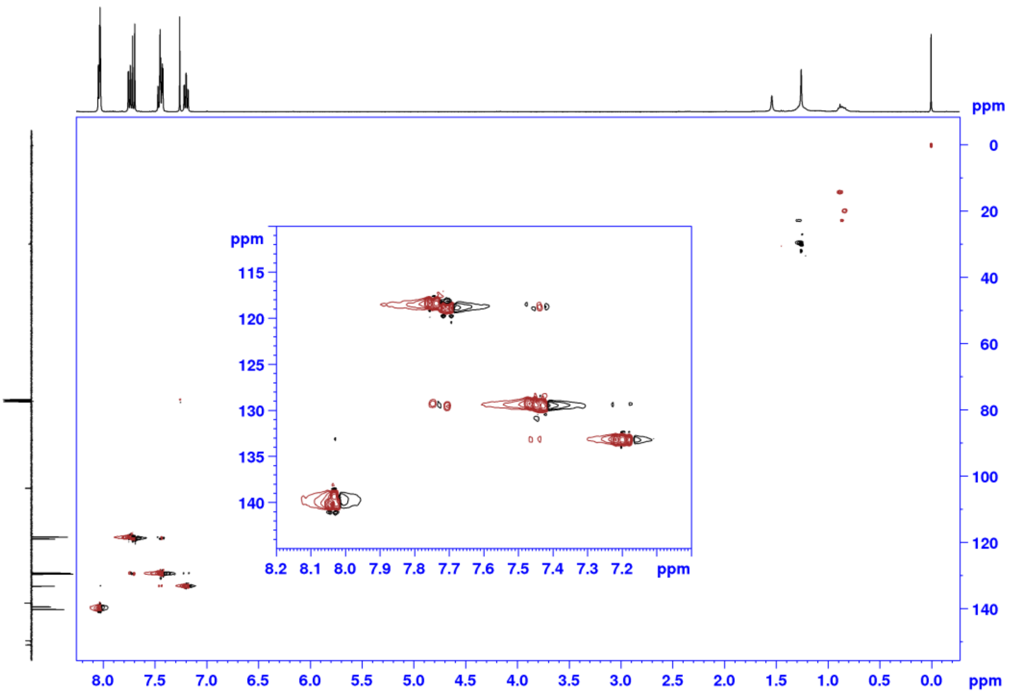

Figure S109: HSQC spectrum of **L6I-V** in $CDCl_3$ (400 MHz).

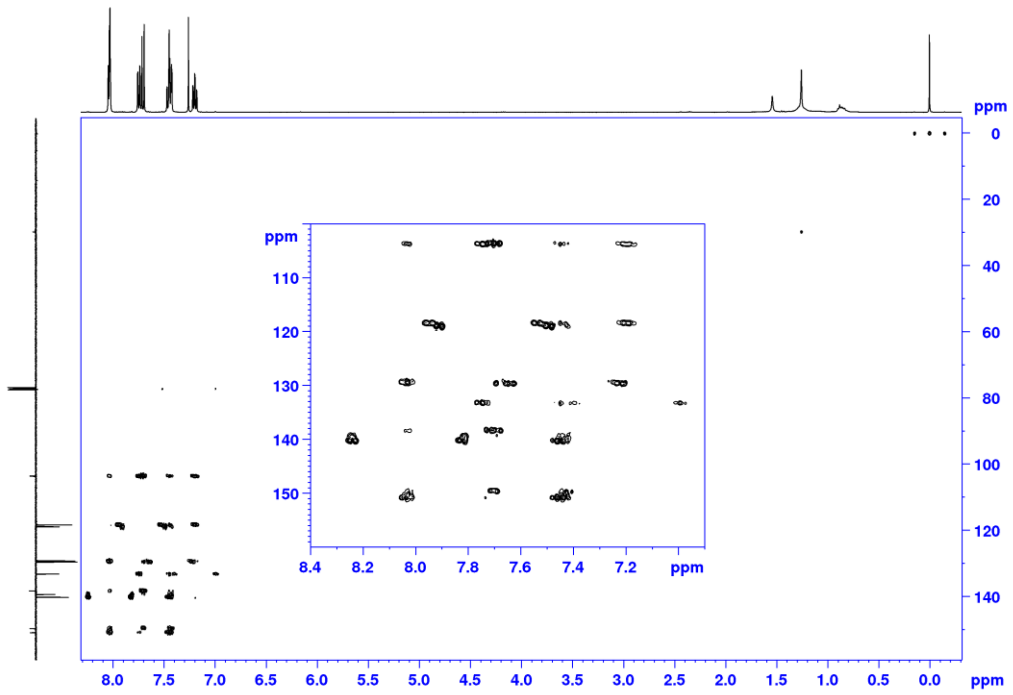

Figure S110: HMBC spectrum of **L6I-V** in $CDCl_3$ (400 MHz).

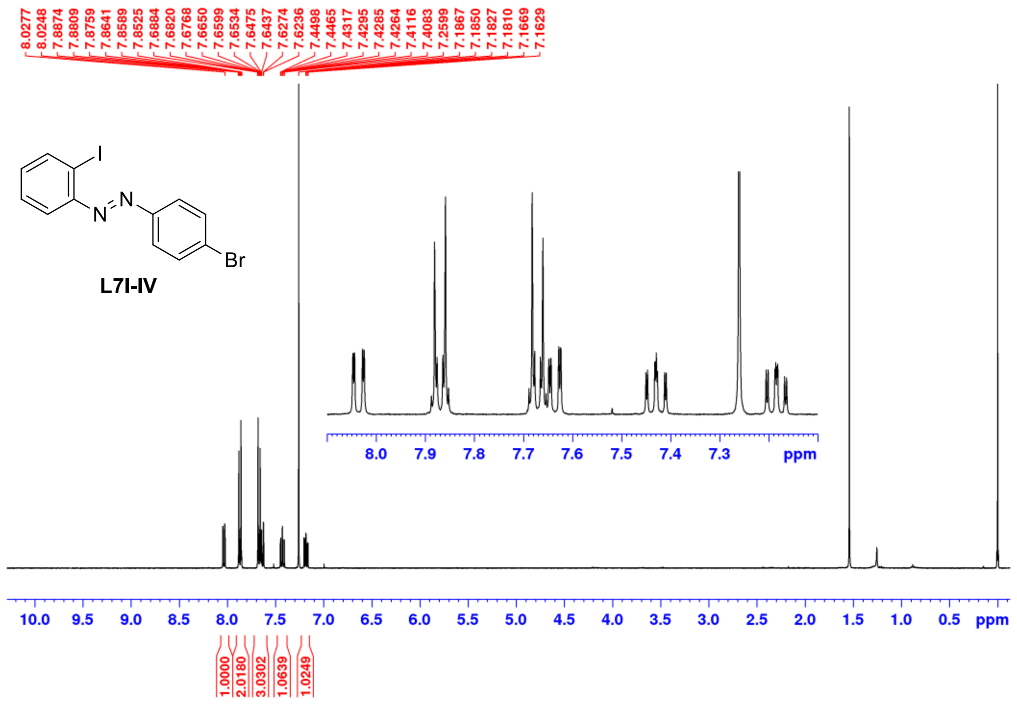

Figure S111: ^{1}H NMR spectrum of **L7I-IV** in $CDCl_3$ (400 MHz).

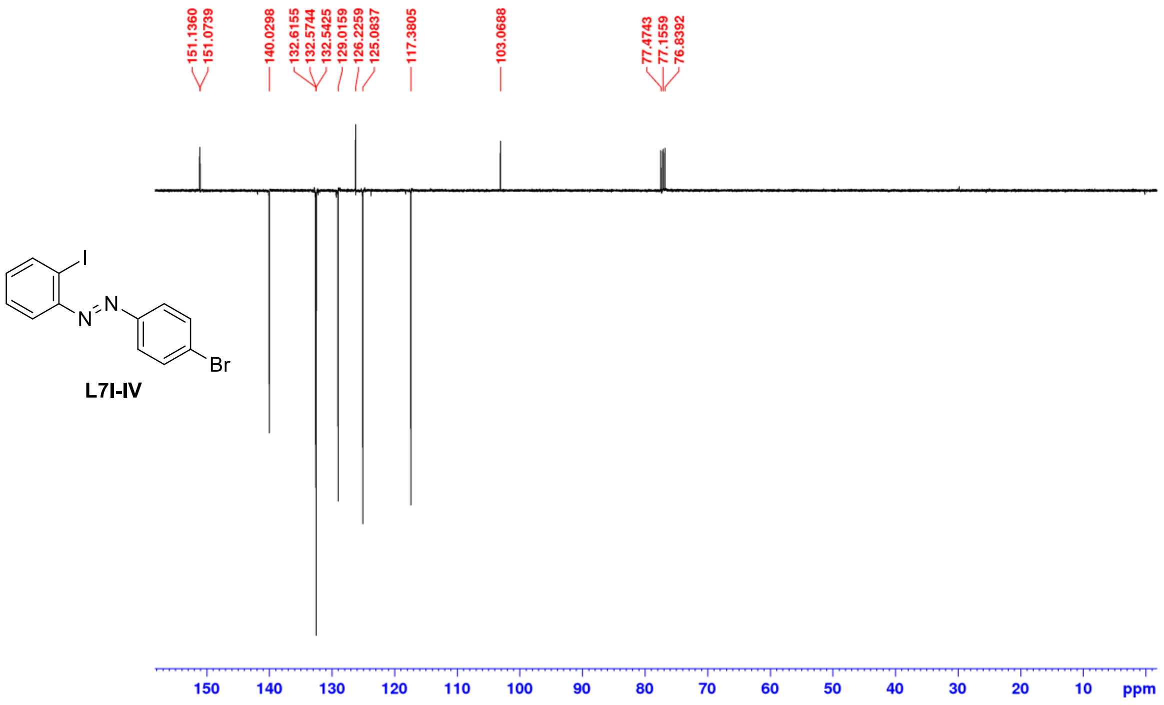

Figure S112: DEPTQ NMR spectrum of **L7I-IV** in $CDCl_3$ (101 MHz).

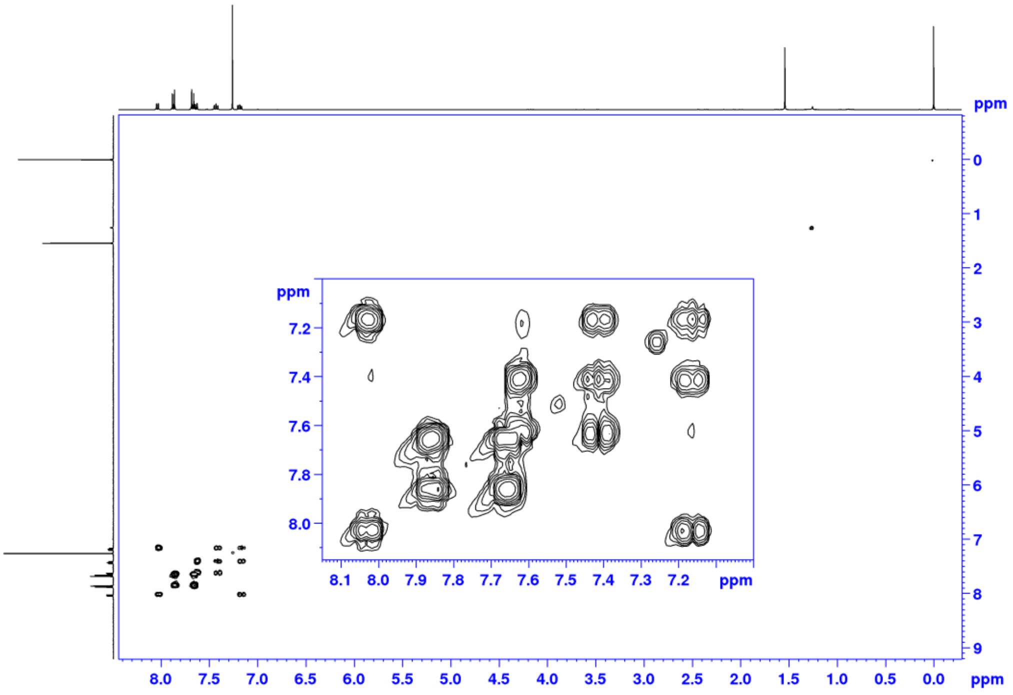

Figure S113: COSY spectrum of **L7I-IV** in $CDCl_3$ (400 MHz).

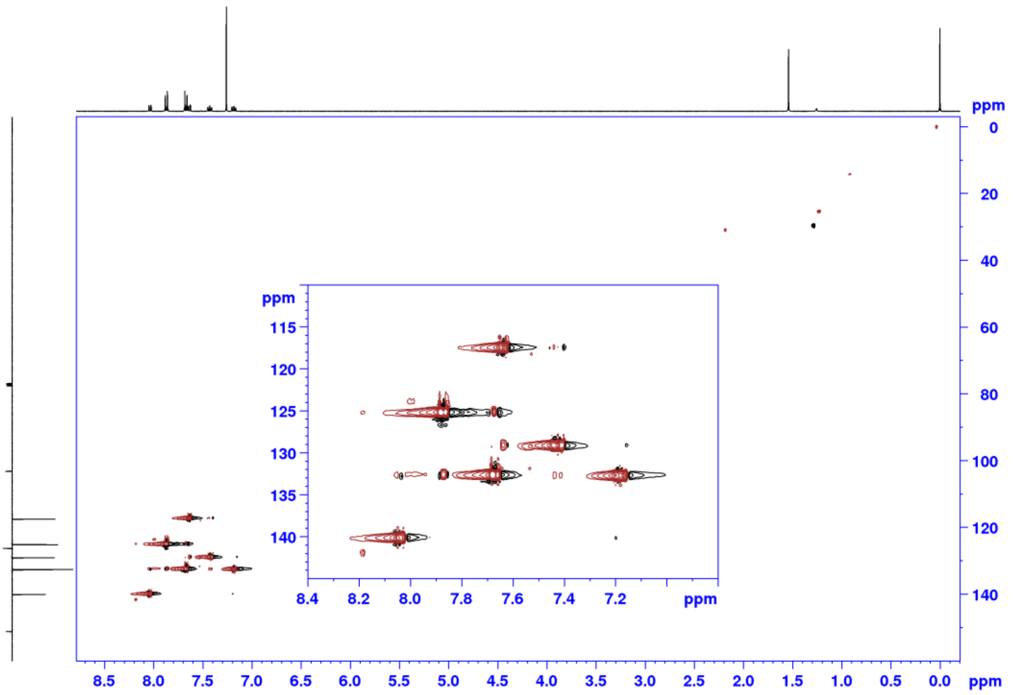

Figure S114: HSQC spectrum of **L7I-IV** in $CDCl_3$ (400 MHz).

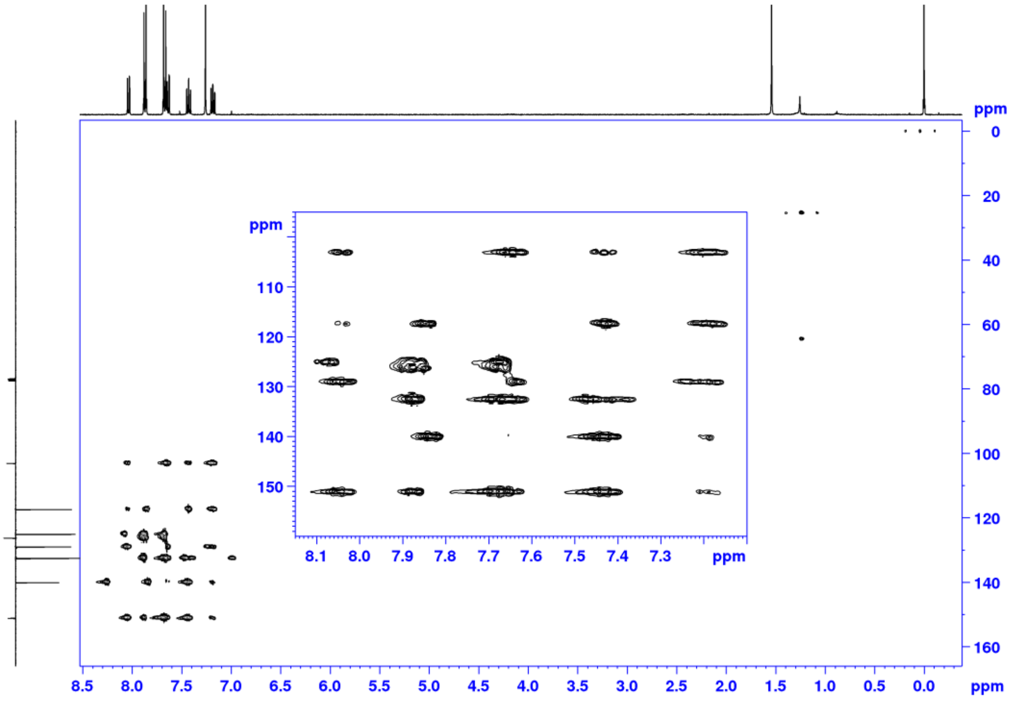

Figure S115: HMBC spectrum of **L7I-IV** in $CDCl_3$ (400 MHz).

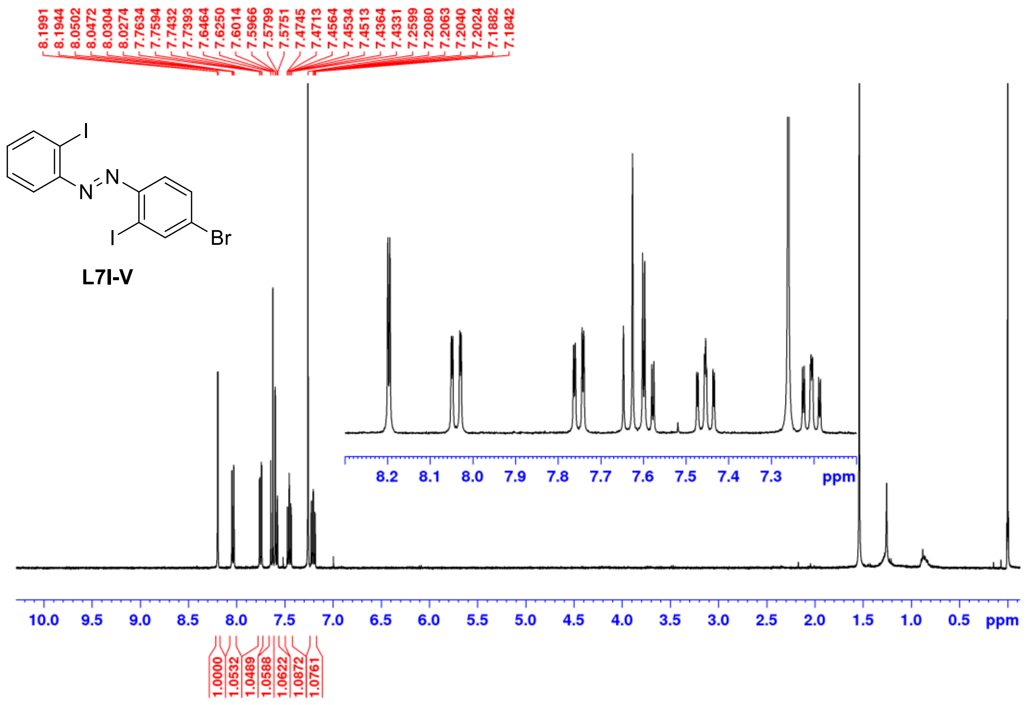

Figure S116: ^{1}H NMR spectrum of **L7I-V** in $CDCl_3$ (400 MHz).

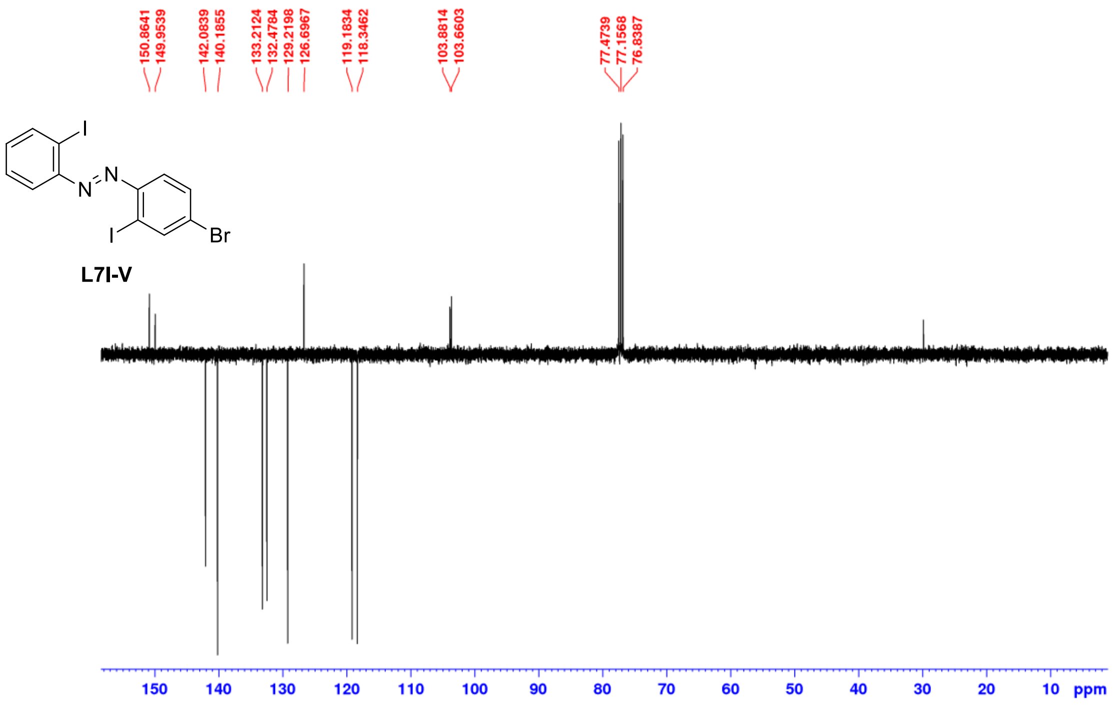

Figure S117: DEPTQ NMR spectrum of **L7I-V** in $CDCl_3$ (101 MHz).

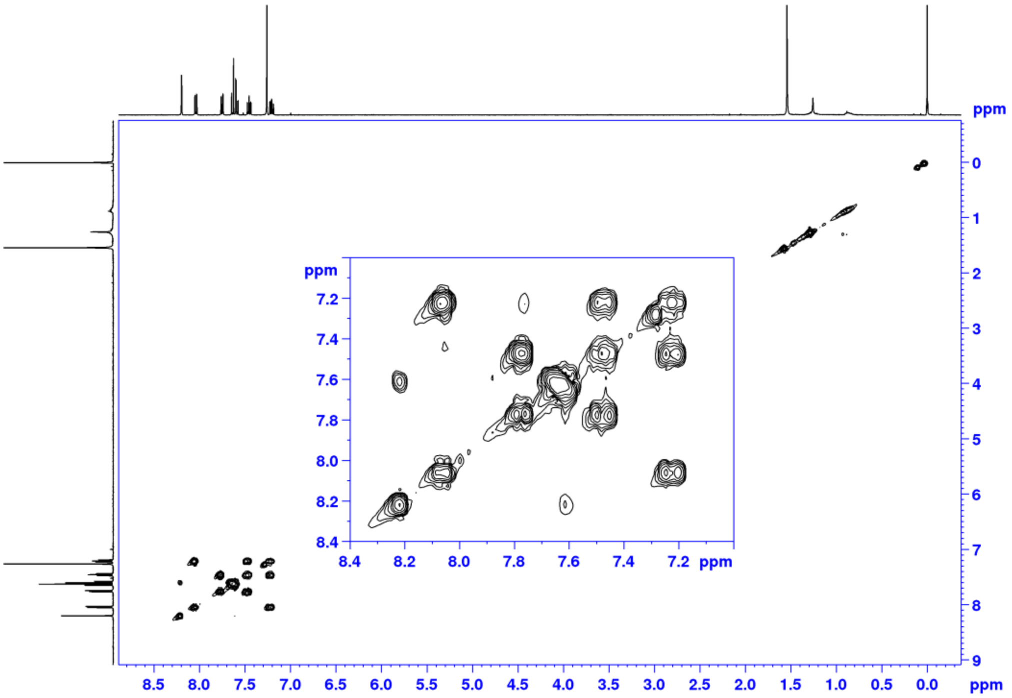

Figure S118: COSY spectrum of **L7I-V** in $CDCl_3$ (400 MHz).

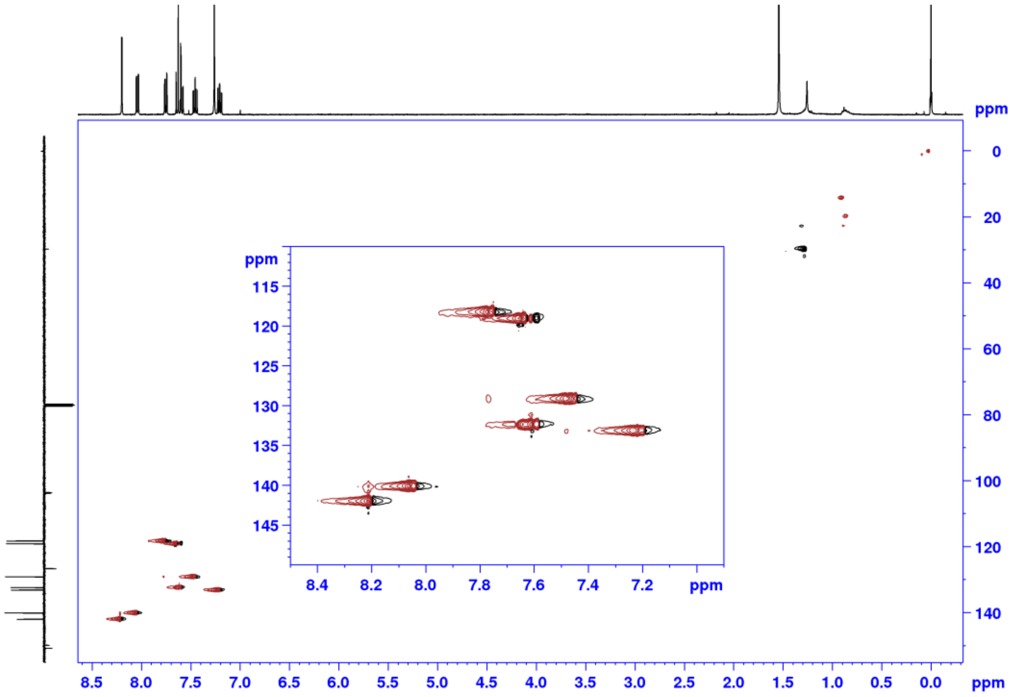

Figure S119: HSQC spectrum of **L7I-V** in $CDCl_3$ (400 MHz).

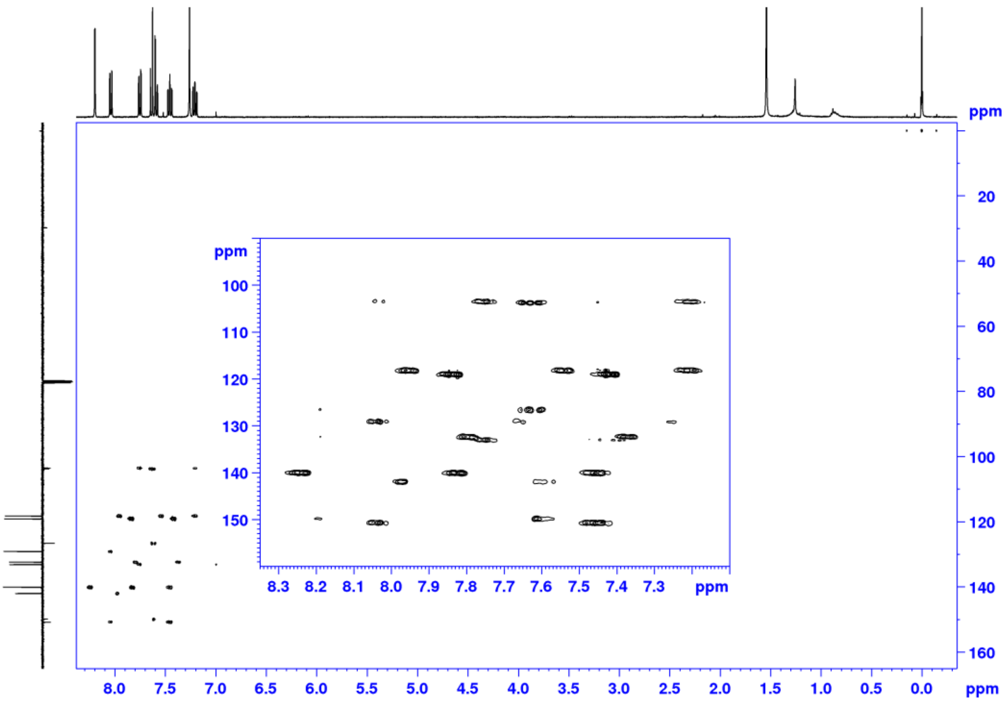

Figure S120: HMBC spectrum of **L7I-V** in $CDCl_3$ (400 MHz).

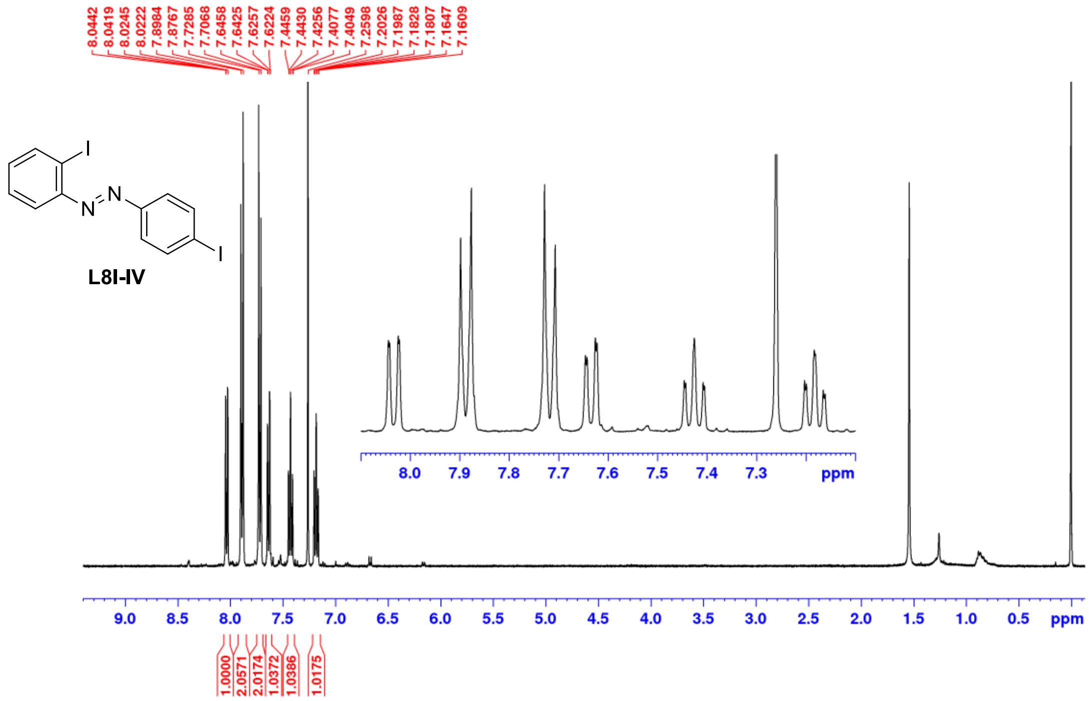

Figure S121: ^{1}H NMR spectrum of **L8I-IV** in $CDCl_3$ (400 MHz).

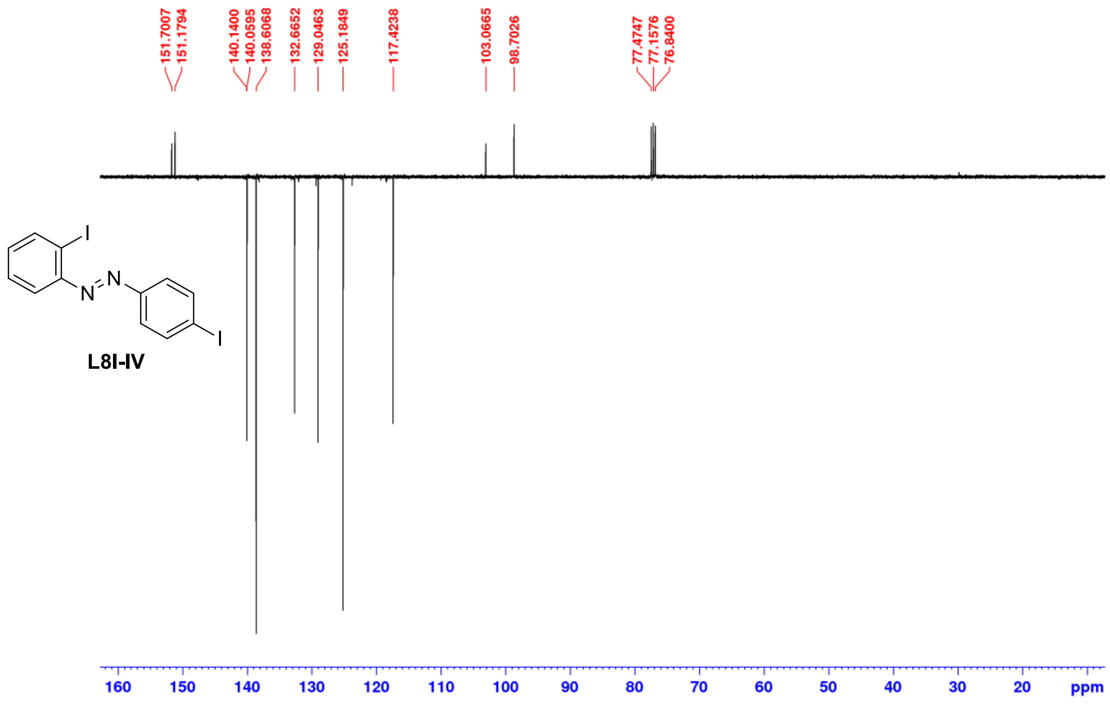

Figure S122: DEPTQ NMR spectrum of **L8I-IV** in $CDCl_3$ (101 MHz).

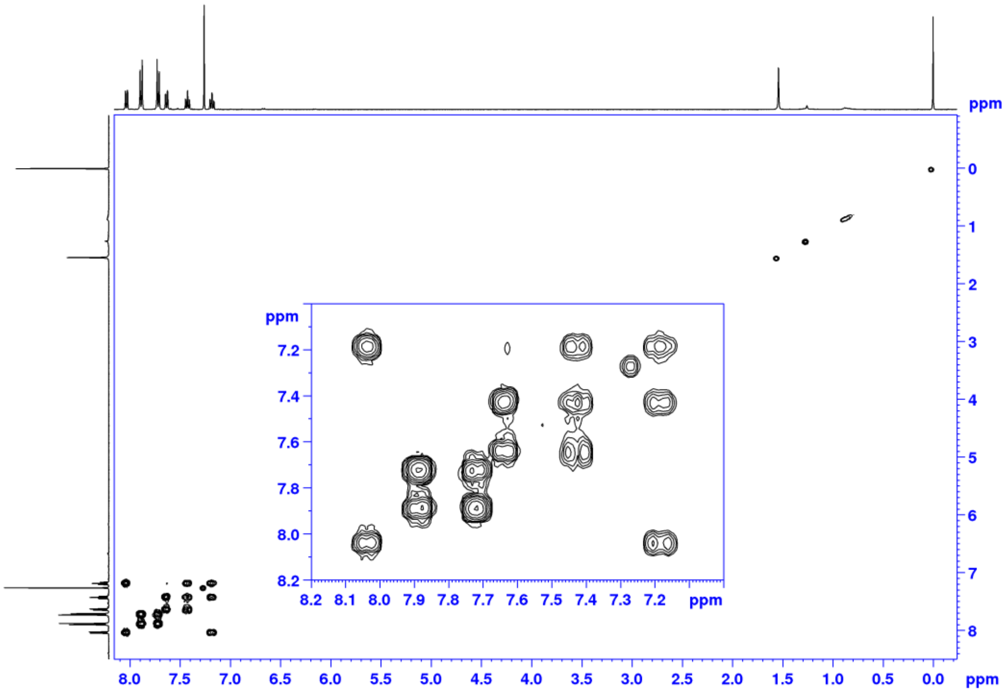

Figure S123: COSY spectrum of **L8I-IV** in $CDCl_3$ (400 MHz).

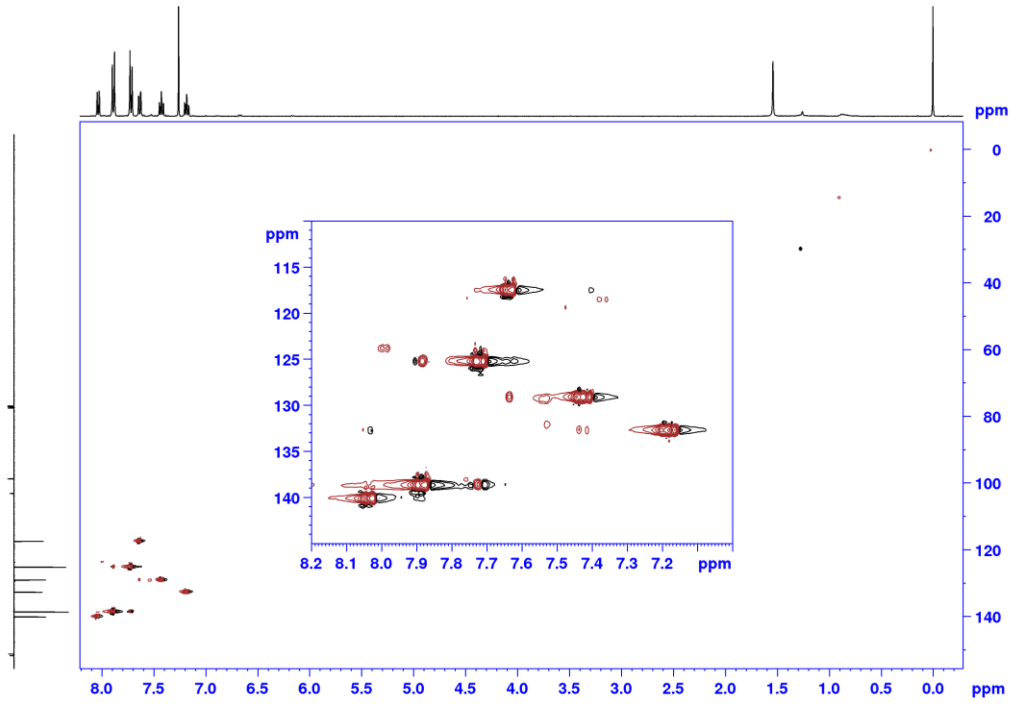

Figure S124: HSQC spectrum of **L8I-IV** in $CDCl_3$ (400 MHz).

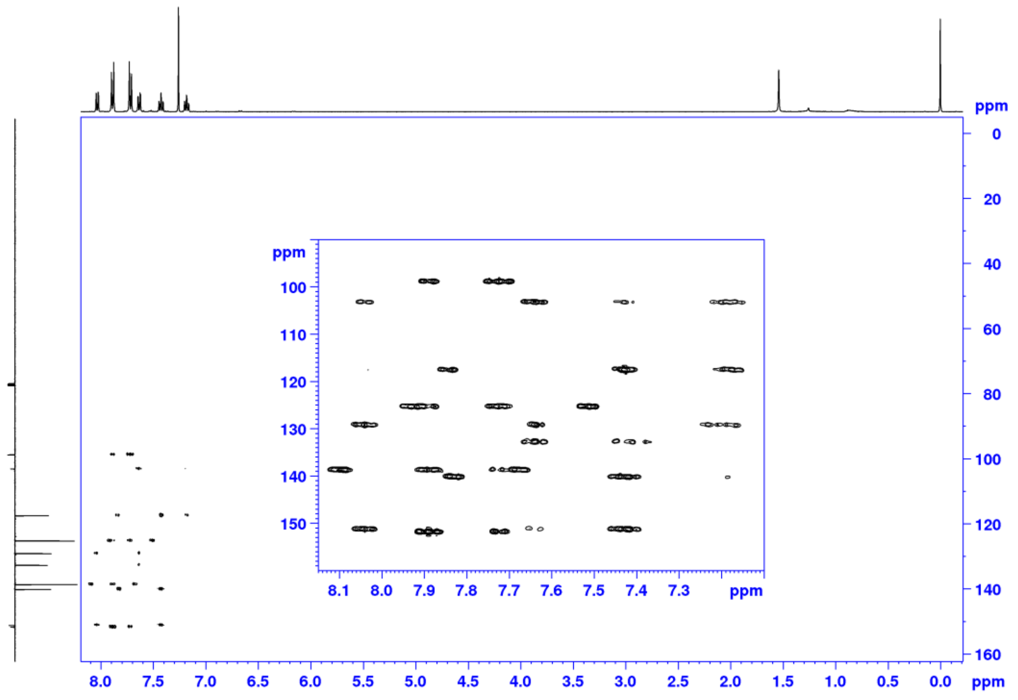

Figure S125: HMBC spectrum of **L8I-IV** in $CDCl_3$ (400 MHz).

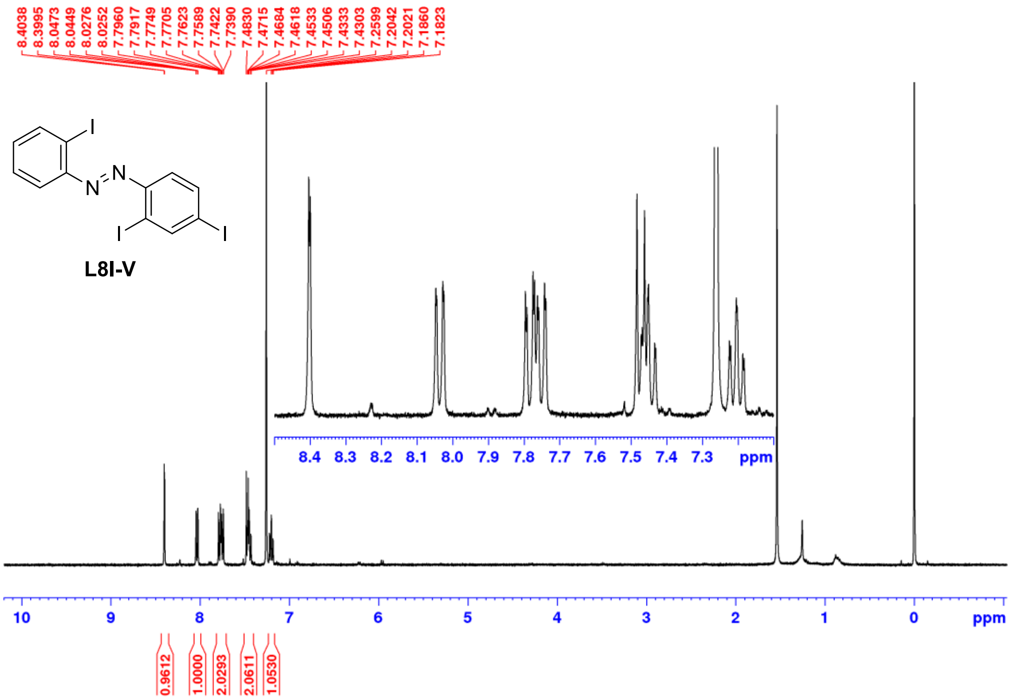

Figure S126: ^{1}H NMR spectrum of **L8I-V** in $CDCl_3$ (400 MHz).

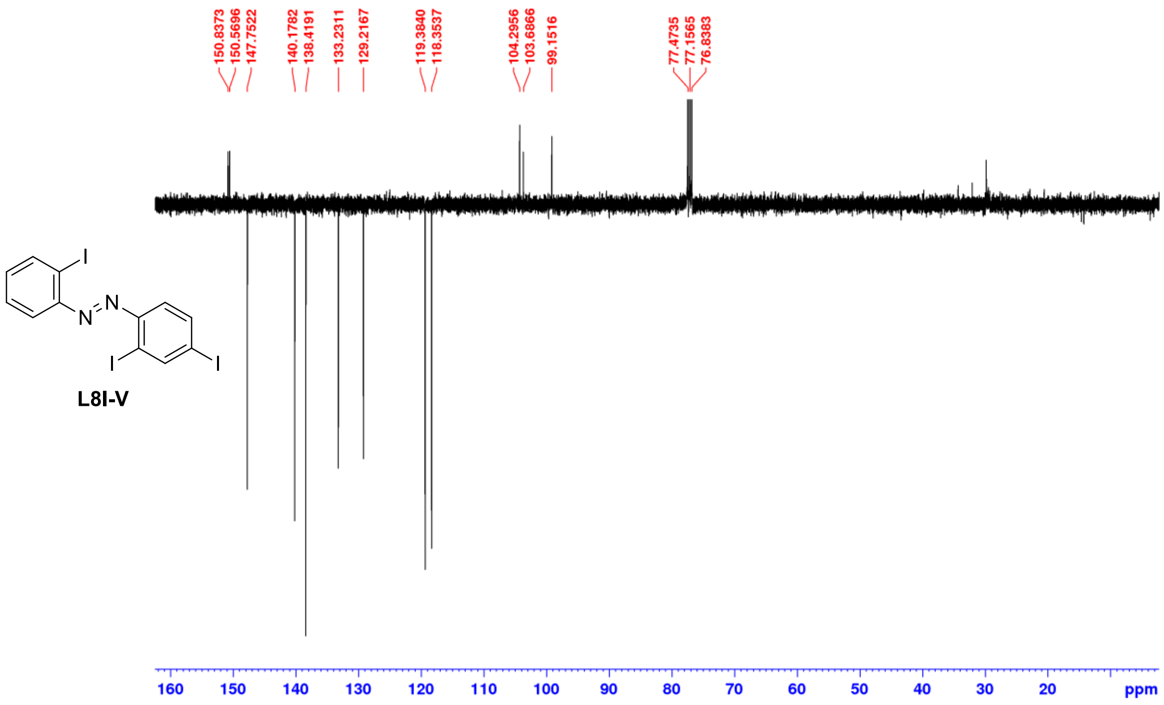

Figure S127: DEPTQ NMR spectrum of **L8I-V** in $CDCl_3$ (101 MHz).

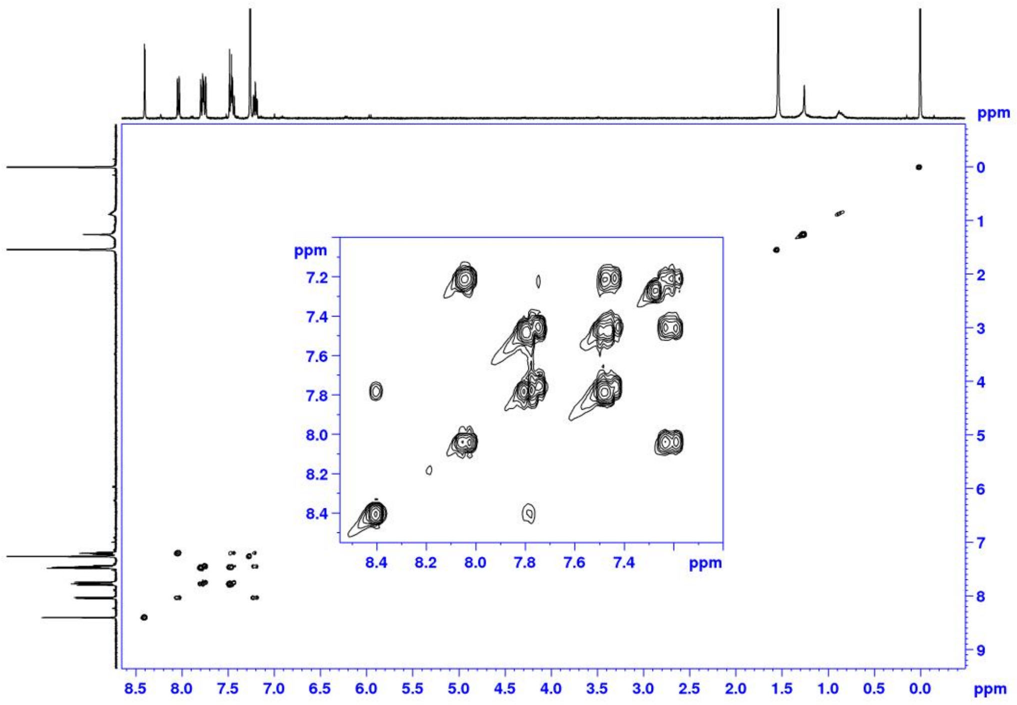

Figure S128: COSY spectrum of **L8I-V** in $CDCl_3$ (400 MHz).

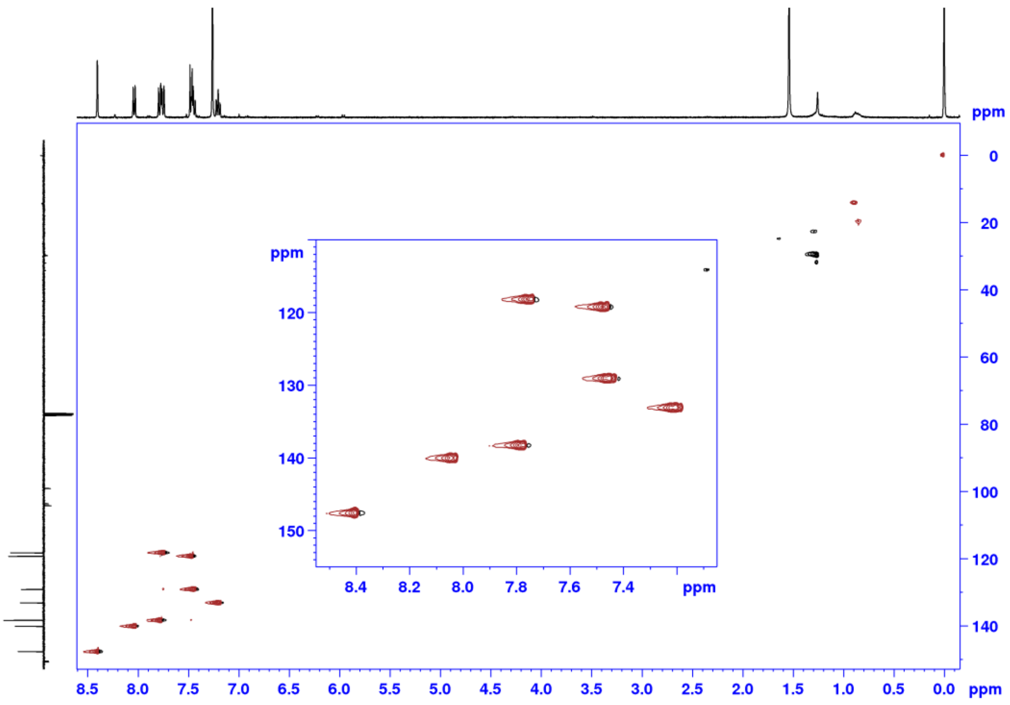

Figure S129: HSQC spectrum of **L8I-V** in $CDCl_3$ (400 MHz).

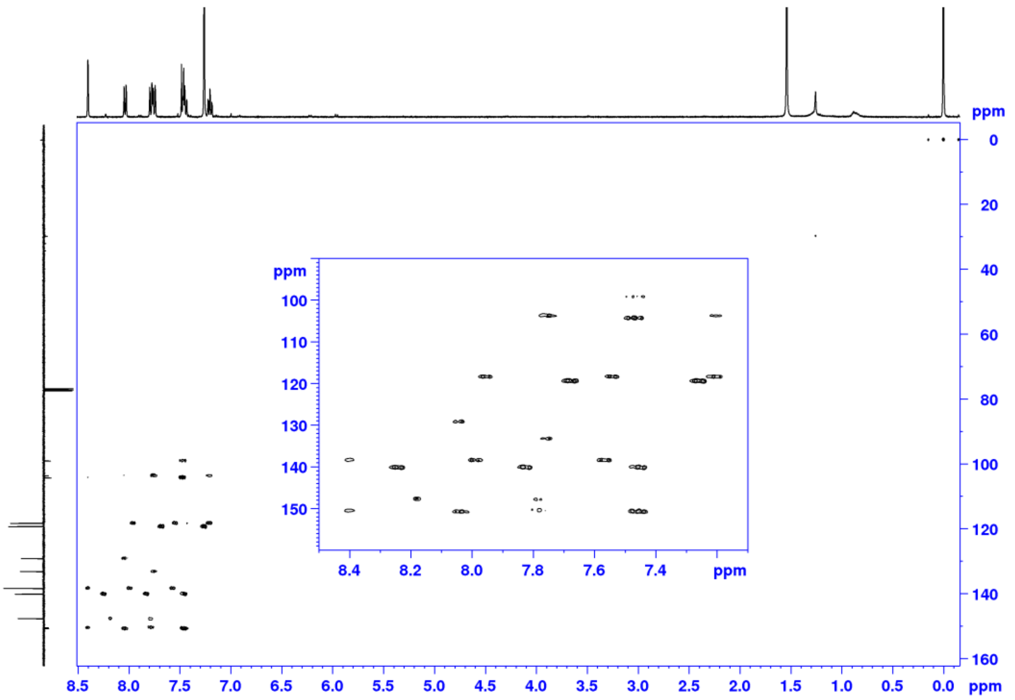

Figure S130: HMBC spectrum of **L8I-V** in $CDCl_3$ (400 MHz).

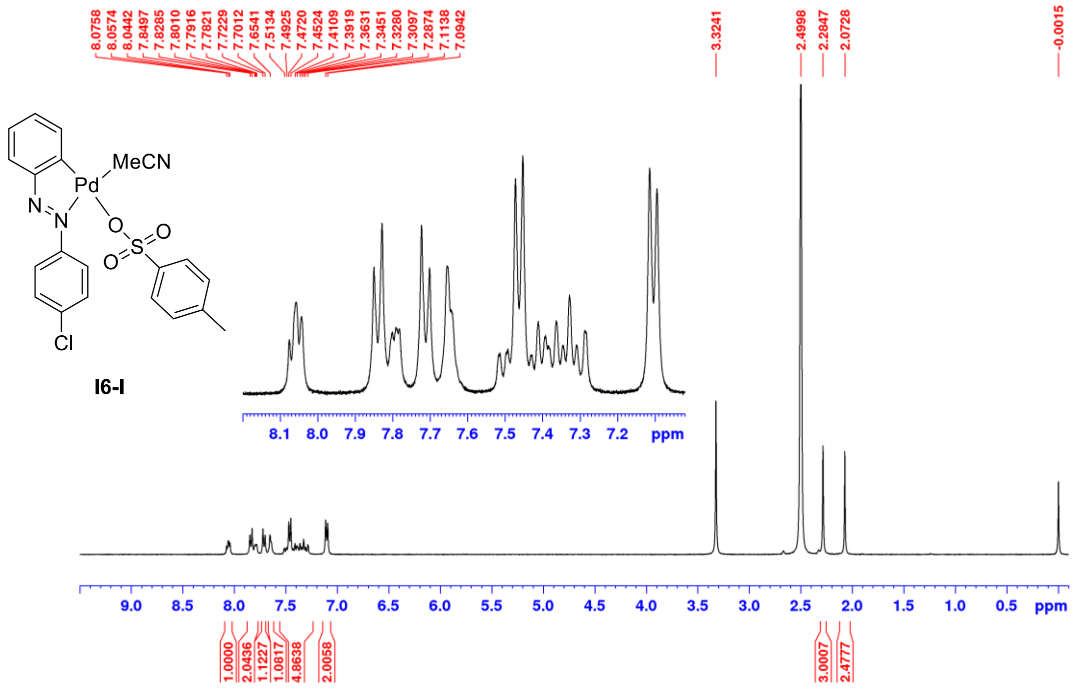

Figure S131: ^{1}H NMR spectrum of **I6-I** in DMSO-d_6 (400 MHz). DMSO-d_6 as a strong donor solvent replaces the MeCN ligand in **I6-I** complex upon disolution of **I6-I** in DMSO-d_6.

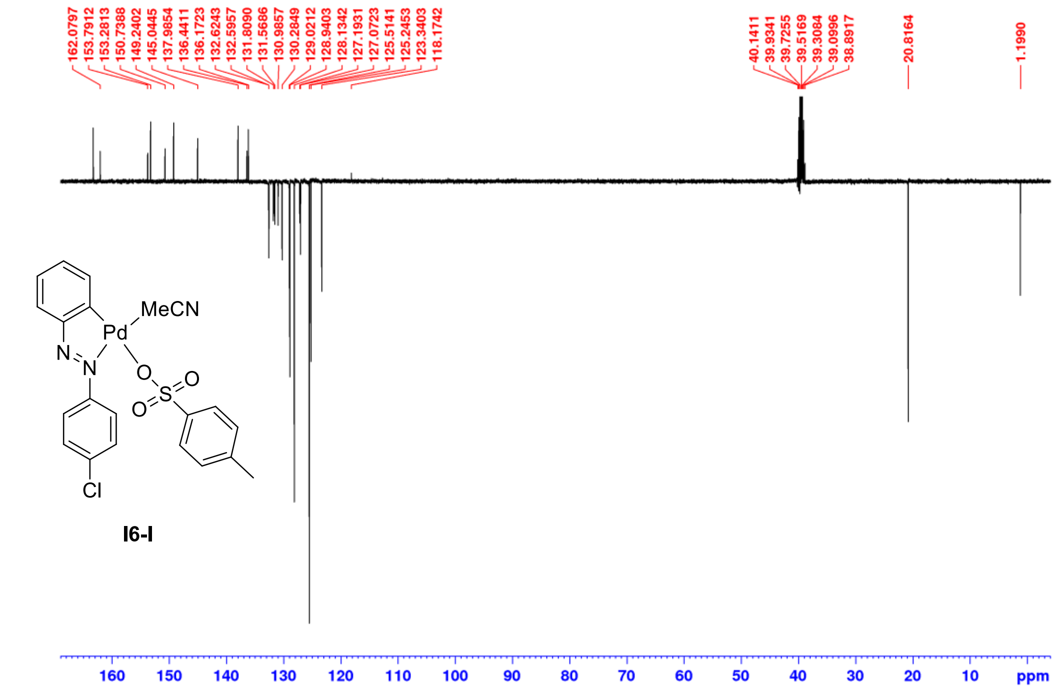

Figure S132: DEPTQ NMR spectrum of **I6-I** in DMSO-d_6 (101 MHz).

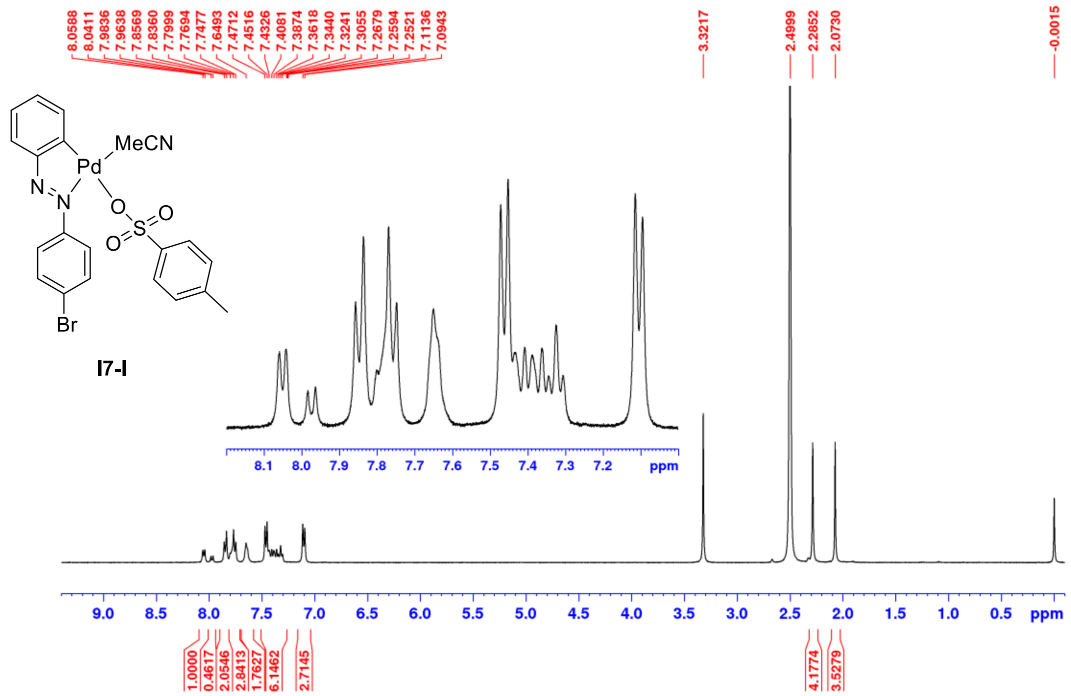

Figure S133: ^{1}H NMR spectrum of **I7-I** in DMSO-d_6 (400 MHz). DMSO-d_6 as a strong donor solvent replaces the MeCN ligand in **I7-I** complex upon disolution of **I7-I** in DMSO-d_6.

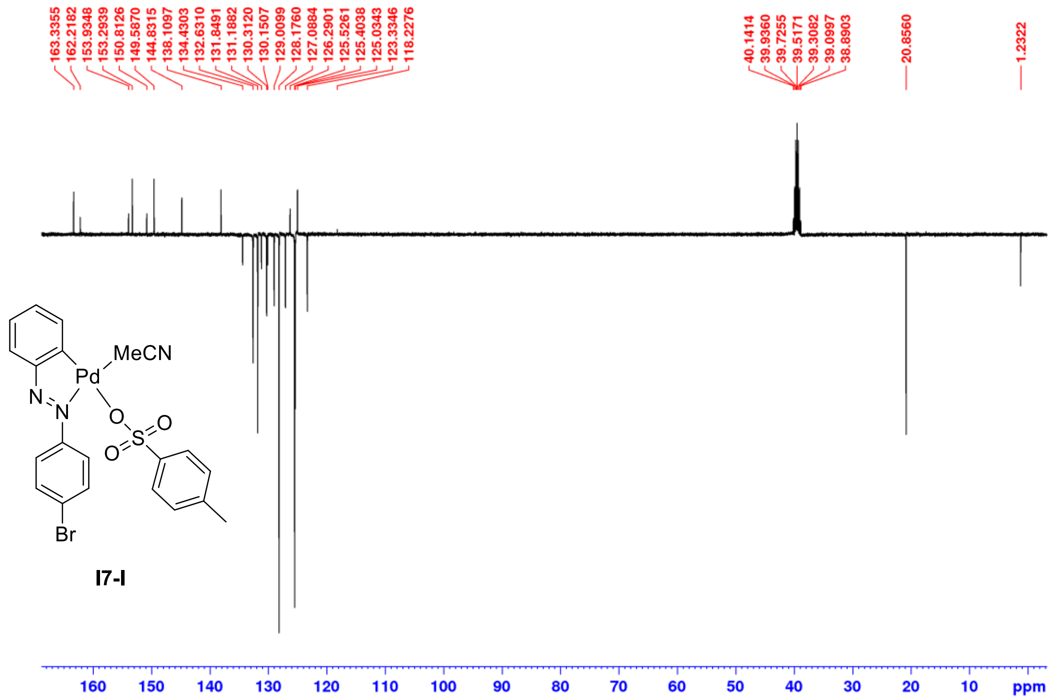

Figure S134: DEPTQ NMR spectrum of **I7-I** in DMSO-d_6 (101 MHz).

8. References

(1) Drent, E.; Van Broekhoven, J. A. M.; Doyle, M. J. *J. Organomet. Chem.* **1991**, *417* (1–2), 235–251. doi:10.1016/0022-328X(91)80176-K

(2) Agilent, CrystAlis PRO. Agilent Technologies Ltd: Yamton, Oxfordshire, England 2014

(3) Oxford Diffraction. Xcalibur CCD system, CrysAlis CCD and CrysAlis RED software 2003

(4) Dolomanov, O. V.; Bourhis, L. J.; Gildea, R. J.; Howard, J. A. K.; Puschmann, H. *J. Appl. Crystallogr.* **2009**, *42* (2), 339–341. doi:10.1107/S0021889808042726

(5) Sheldrick, G. M. *Acta Crystallogr. Sect. A Found. Crystallogr.* **2008**, *64* (1), 112–122. doi:10.1107/S0108767307043930

(6) Sheldrick, G. M. *Acta Crystallogr. Sect. C Struct. Chem.* **2015**, *71* (Md), 3–8. doi:10.1107/S2053229614024218

(7) Macrae, C. F.; Edgington, P. R.; McCabe, P.; Pidcock, E.; Shields, G. P.; Taylor, R.; Towler, M.; Van De Streek, J. *J. Appl. Crystallogr.* **2006**, *39* (3), 453–457. doi:10.1107/S002188980600731X

(8) Barišić, D.; Halasz, I.; Bjelopetrović, A.; Babić, D.; Ćurić, M., *Organometallics*, **2022**. doi:10.1021/acs.organomet.1c00698

(9) Cai, S.; Rong, H.; Yu, X.; Liu, X.; Wang, D.; He, W.; Li, Y. *ACS Catal.* **2013**, *3* (4), 478–486. doi:10.1021/cs300707y

(10) Schultzke, S.; Walther, M.; Staubitz, A. *Molecules* **2021**, *26* (13), 3916. doi:10.3390/molecules26133916

(11) Ma, X. T.; Tian, S. K. *Adv. Synth. Catal.* **2013**, *355* (2–3), 337–340. doi:10.1002/adsc.201200902
